# Selection between Competing Self-Reproducing Lipids: Succession and Dynamic Activation

**DOI:** 10.1021/jacsau.1c00138

**Published:** 2021-08-16

**Authors:** Michael G. Howlett, Robert J. H. Scanes, Stephen P. Fletcher

**Affiliations:** Department of Chemistry, Chemistry Research Laboratory, University of Oxford, Oxford OX1 3TA, United Kingdom

**Keywords:** origins of life, autocatalysis, selection, self-reproducing lipids, self-replication, molecular evolution, dynamic covalent, reaction networks

## Abstract

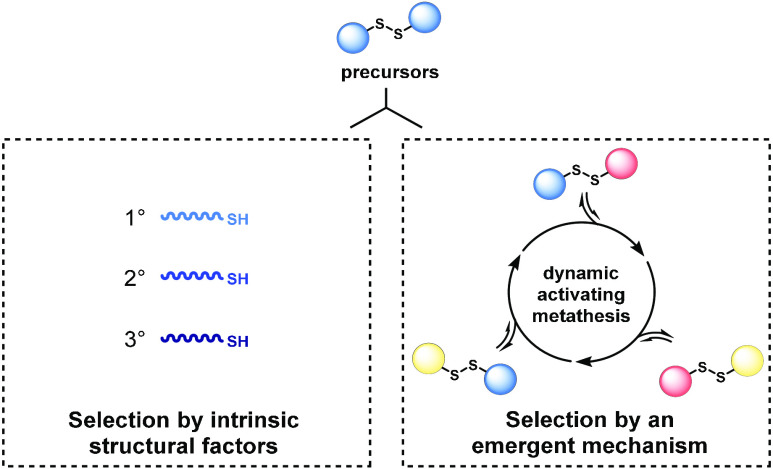

Models of chemical evolution are central to advancing origins of life research. To design more lifelike systems, we must expand our understanding of molecular selection mechanisms. Here, we show two selection modes that produce evolving populations of self-reproducing species, formed through thiol–disulfide exchange. Competition between thiol precursors can give clear succession patterns based on steric factors, an intrinsic property. A separate, emergent selection mechanism—dynamic activating metathesis—was found when exploring competing disulfide precursors. These experiments reveal that additional species generated in the mixture open up alternative reaction pathways to form self-reproducing products. Thus, increased compositional complexity provides certain species with a unique competitive advantage at the expense of others.

## Introduction

Our prebiotic origins and chemical evolution are at the heart of much systems chemistry research. It is expected that a diverse pool of molecules underwent various chemical adaptation, replication, and selection processes before reaching familiar biological assemblies.^1^ Understanding how selection may arise is key to ongoing research in dynamic systems, synthetic protocells, and replicator networks.^2−4^ Even so, little is known about what selection mechanisms are available to simple replicating species competing for resources. Progress has been made regarding plausible routes to building blocks for living cells,^2,5−7^ but questions remain about how dominant self-replicating motifs can emerge from prebiotic beginnings.^8,9^

Species that can replicate possess clear advantages over those which cannot and are relevant to theories of chemical evolution. Inspired by nature’s functional biopolymers and the “RNA world” hypothesis,^6,10,11^ template-based molecular replication has long been a major feature of prebiotic chemistry research. For example, template replicators based upon nucleotides,^12^ fiber stacking,^13^ Diels–Alder,^14^ and dipolar cycloaddition chemistry^15^ have all been reported. In contrast, self-reproducing surfactants possess their own unique abilities and selection rules, in line with the “lipid world” theory.^16,17^ Surfactants can accelerate their own formation by forming aggregates which aid in the reaction between their phase-separated precursors, in a process termed physical autocatalysis.^18−22^ Importantly, where multiple self-reproducing species are present, surfactant production can result in a global cross-catalytic effect. Complex systems of this type can lead to ill-defined scenarios where the replication effects are tied to the system as a whole, rather than individual self-serving species.

**Scheme 1:**
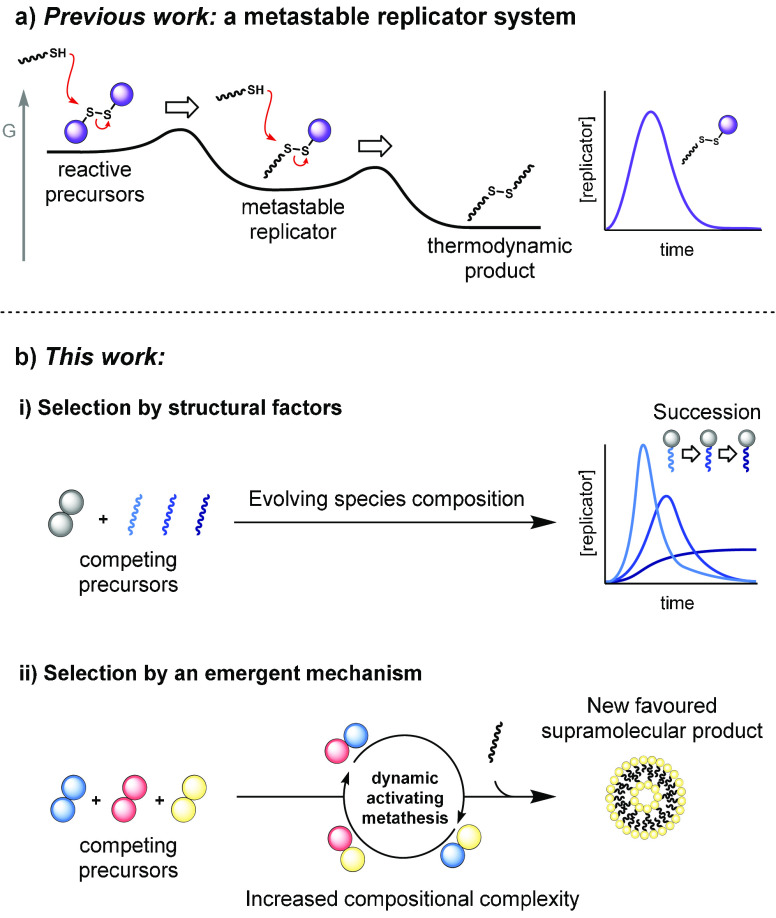
Selection of Metastable Self-Reproducing Surfactants Formation and destruction steps cause population growth and decline for a kinetically stable replicator. New selection modes in a competing surfactant system generate succession patterns and competitive advantages through intrinsic structural factors (i) and dynamic activation (ii).

Selection between species is important in models of the development of life. Past studies into template replicator selection have typically relied upon competing auto- and cross-catalytic processes, providing valuable insight into the interplay between species.^13,23−26^ However, by virtue of their stability, these replicators primarily exist close to thermodynamic equilibrium—the domain of inanimate materials.^27,28^ Recognizing life as an inherently dissipative state has prompted the development of metastable systems to uncover more lifelike phenomena (e.g., Scheme 1a).^29−33^

Selection in metastable systems has been reported in a few cases and can arise from factors such as precursor solubility under heterogeneous conditions,^34^ selective degradation,^35−37^ rate of assembly,^38^ and product compartmentalization.^39^ These cases highlight the breadth of potential selection mechanisms available to lifelike systems. Further selection modes await discovery and could shed light on how chemical selection may serve as a basis for early biological evolution. For example, it seems plausible that certain mixtures may have functions not available to their isolated components.^40−42^

In previous work, we described a metastable self-replicator formed through thiol–disulfide exchange.^32^ This established a surfactant-forming reaction between thiol **1a** and disulfide **2a** to give self-reproducing species **4aa**. Here, we identify new selection mechanisms that arise from using multiple competing self-reproducing surfactants. We obtain increased levels of compositional complexity which vary in time and clearly observe evolving product populations, i.e., chemical succession (Scheme 1b).

## Results and Discussion

**Scheme 2:**
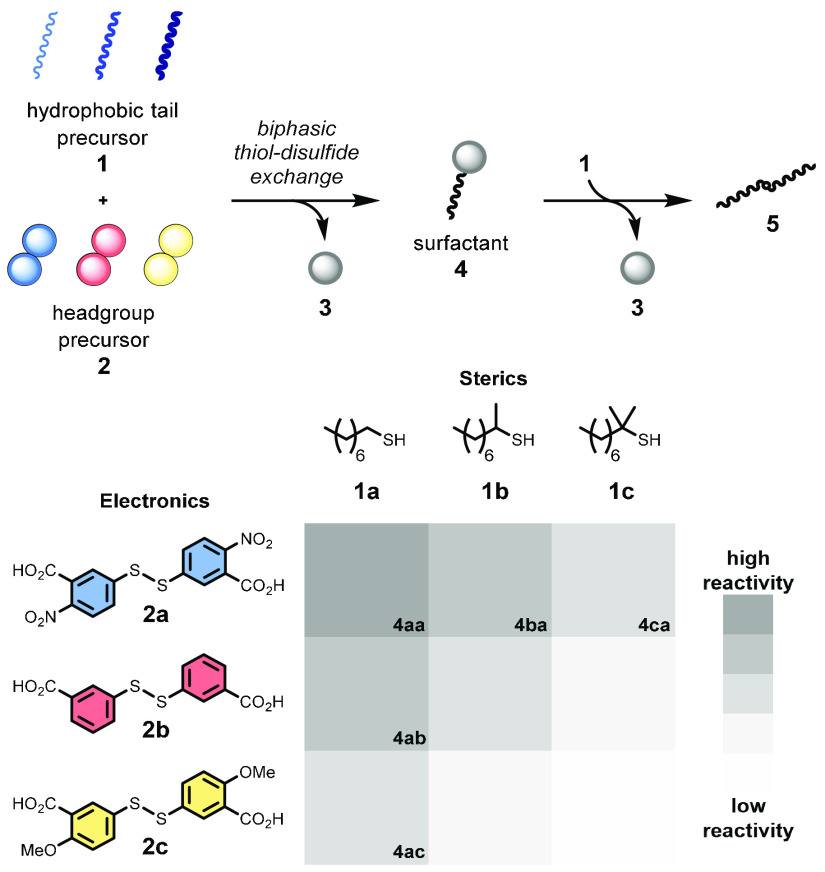
Surfactant Precursors and Their Observed Relative Reactivity in the Studied Thiol–Disulfide Exchange Three surfactant series with various headgroups (**a**, **b**, and **c**) and hydrophobic tail are accessible. The surfactant products are labelled analogously to the following example: **4ac** = tail **a** + headgroup **c**. Two irreversible thiol–disulfide exchange reactions in turn form and then break down metastable species **4**. **5** represents the generic corresponding dialkyl disulfide product formed from the parent thiol(s). Reactivity is proportional to the relative **4** formation and destruction rates from independent controls. Full kinetic data may be found in the SI.

A system of competing products was envisaged, formed from a reaction between three hydrophobic thiol (**1**) and three hydrophilic disulfide (**2**) building blocks (see Scheme 2). When reacted, these precursors give a total of nine heterodisulfide surfactants (**4**) which can accelerate their own formation. The different precursors vary in nucleophilicity of the thiol (sterics) and electrophilicity of the disulfide (electronics). Thiol–disulfide exchange reactions give rise to the formation and destruction of each surfactant, determining their lifetime. At the system level, it is the network of interactions between these self-reproducing species and their precursors which is responsible for the phenomena observed. When phase-separated alkyl thiol **1** attacks electrophilic disulfide **2** it gives surfactant **4** and product **3**. Next, a second equivalent of **1** reacts with **4**, depleting the lipid population and giving products **3** and **5**. Consistent with our prior work, this surfactant-forming process shows autocatalytic reaction kinetics by virtue of the interfacial reaction steps involved (see Figures S26 and S27).^32^ Due to the constant turnover of the metastable products (**4**), we believed we would observe succession analogous to that seen in ecological systems of the natural world.

### Reactivity Trends Established in Non-competition Reactions

We performed nine independent reactions, combining each nucleophilic thiol **1a**–**1c** with each disulfide **2a**–**2c** in turn. Increasing the steric hindrance of the alkyl thiol (and the product surfactant) by varying the methyl substitution resulted in lower formation and destruction rates, i.e., decreased reactivity. Lower reactivity was also seen when using more electron-rich aromatic disulfides **2b**–**2c**. The reactivity patterns from these combinations are summarized in Scheme 2 (see Figure S12 for details).

Remarkably, dynamic light scattering (DLS) of the surfactants highlighted a further trend associated with their electronic properties. While electron-poor headgroup **a** gives rise to micellar structures, surfactants in series **b** or **c** possess size distributions consistent with vesicles (see Figures S34–S39). This difference is possibly due to the relative acidity of the carboxylic acid groups found on aggregated monomers, where altering the charge density and headgroup repulsion (i.e., electronics) influences the packing of monomers.^43^ This phenomenon is observed with oleic acid, where at pH >10.2 micelles form, whereas below this value vesicles are found.^44^

### Species Succession by Intrinsic Structural Factors

**Scheme 3:**
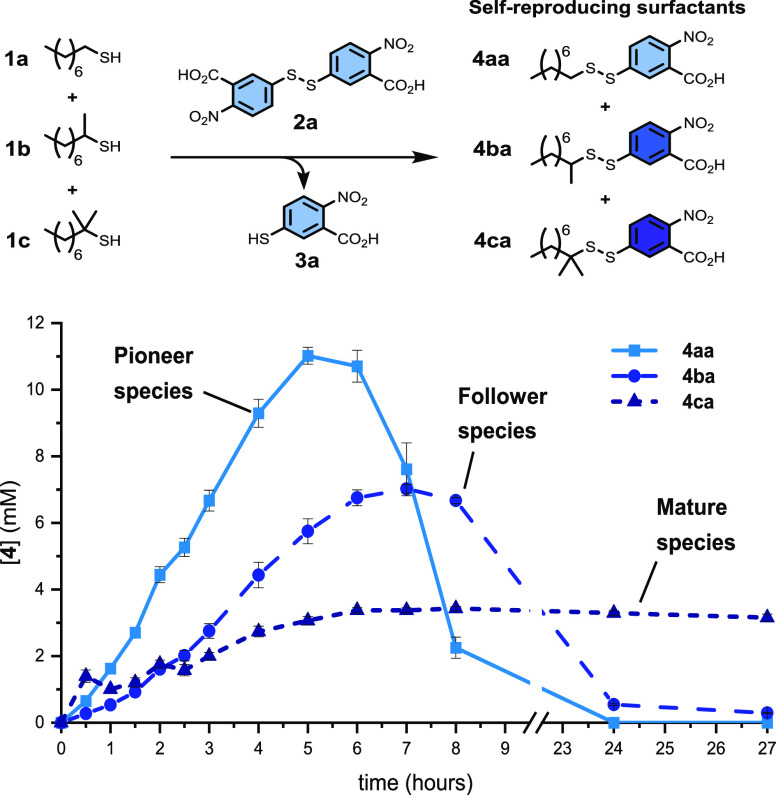
Competition between Sterically Differentiated Precursors Leads to Species Succession in Time Destruction step with concomitant formation of product **5** omitted for clarity. *n* = 3; error bars (where visible) represent the standard deviation.

We then examined systems producing competing surfactants, beginning with the influence of steric effects. When reacting a mixture of alkyl thiols **1a**–**1c** with **2a**, a clear succession pattern of the dominant product is seen (Scheme 3). Crucially, the system transitions from one dominant species to another by way of independent formation and destruction steps, rather than direct product interconversion. **4aa** is the fastest to form and shortest lived, followed by **4ba** and then **4ca**. This can be understood based on thiol nucleophilicity and the relative steric hindrance of **4aa**, **4ba**, and **4ca**. As the amount of surfactant **4aa** increases in the system, **4ba** and **4ca** benefit from a cross-catalytic, environmental effect (increased interfacial area) and experience population growth. Hindered species **4ca** has a sufficiently slow destruction step to enable persistence under these conditions and is the dominant species at long reaction times. Ultimately, all metastable surfactants tend toward degrading into products **3** and **5**. The chemical succession shown in Scheme 3 is directly derived from the intrinsic structural properties of precursors **1a**–**1c** and therefore those of **4aa**, **4ba**, and **4ca**.

### Surfactant Selection with Increased Compositional Complexity

**Scheme 4:**
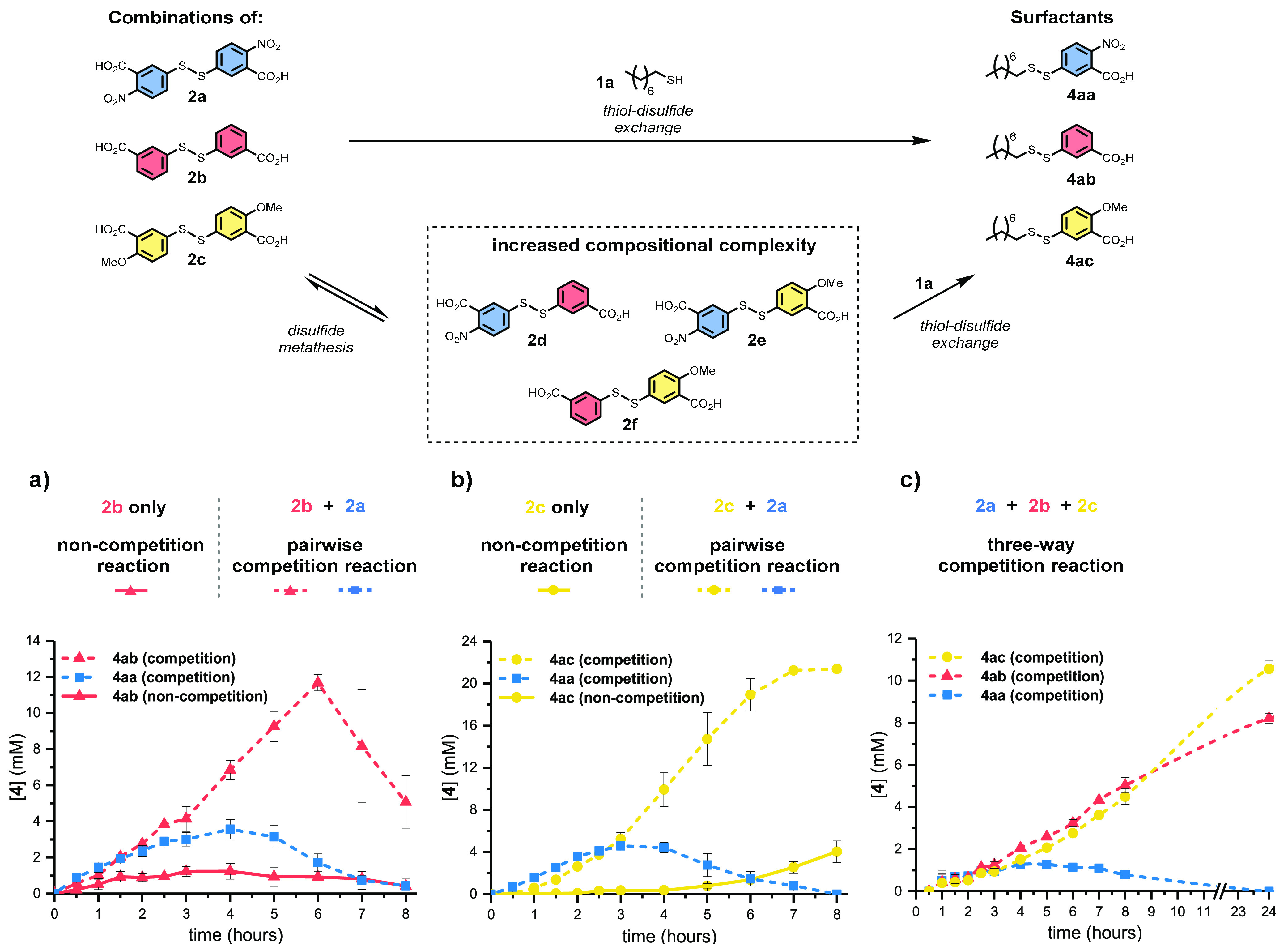
Competition between Electronically Differentiated Precursors Leads to Surfactant Selection over Time by an Emergent Mechanism Overlaid reactions of thiol **1a** with precursor **2b** alone (non-competition) and with a mixture of **2a** and **2b** (competition). Overlaid reactions of thiol **1a** with precursor **2c** alone (non-competition) and with a mixture of **2a** and **2c** (competition). Three-way competition experiment between thiol **1a** and precursors **2a**–**2c**. *n* = 3; error bars (where visible) represent the standard deviation.

Next, we examined combinations of disulfide electrophiles **2a**–**2c**, which have different electronic properties. As shown in Scheme 4, thiol **1a** was reacted with mixtures of disulfides **2a**–**2c**, to give multiple products (**4aa**–**4ac**) in each reaction. First, pairs of disulfides were tested to examine competition between two electrophiles (**2a** + **2b**, Scheme 4a; and **2a** + **2c**, Scheme 4b). The relative proportion of each surfactant was found to change over time. Interestingly, competing products initially formed at similar rates, despite large rate differences observed when formed in isolation (see Schemes 2 and 4).

When disulfide **2a** and thiol **1a** react in isolation, surfactant **4aa** reaches concentrations up to 20 mM (see Figure S13). When competing with **4ab**, [**4aa**] peaks at only ∼4 mM, while **4ab** forms faster than in non-competition experiments (dashed vs solid lines, Scheme 4a). In the competition experiment, **4ab** achieves ∼10× the maximum concentration seen in the absence of disulfide **2a**. Competition between surfactants **4aa** and **4ac** also shows lower [**4aa**] than in isolation (ca. 5 mM), and **4ac** achieves near complete conversion within 8 h, despite being the product of the least electrophilic precursor **2c** (Scheme 4b). When all three surfactants **4aa**, **4ab**, and **4ac** compete, the system displays selection over time (Scheme 4c). As before, **4aa** degrades fastest, decreasing in concentration after peaking at ∼5 h. **4ab** and **4ac** initially perform comparably to one another with **4ac** eventually becoming slightly dominant at long reaction times. These competition experiments show that the formation of **4ab** and **4ac** is enhanced in the presence of additional species. This indicates the emergence of secondary selection factors and the benefits of increased mixture complexity for certain self-reproducing species.

### Dynamic Selection Mechanism through Metathesis

Analysis of reaction UPLC data suggested the presence of new species and led to the discovery of an underlying metathesis between homodisulfides **2a**–**2c**. This fast exchange was shown to give three new heterodisulfides **2d**–**2f** (Scheme 4 and Figure S25).

**Scheme 5:**
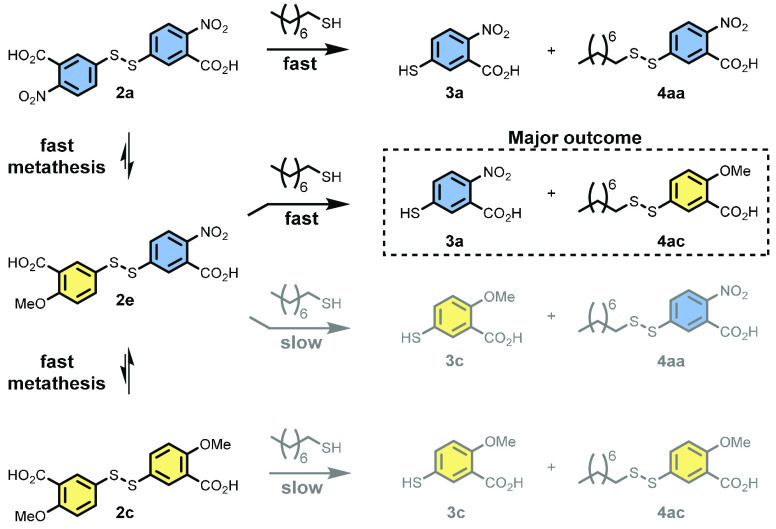
Reaction Pathways in a Competition Reaction: Fast Disulfide Metathesis Producing **2e** Gives Access to an Efficient Route to **4ac**

**Scheme 6:**
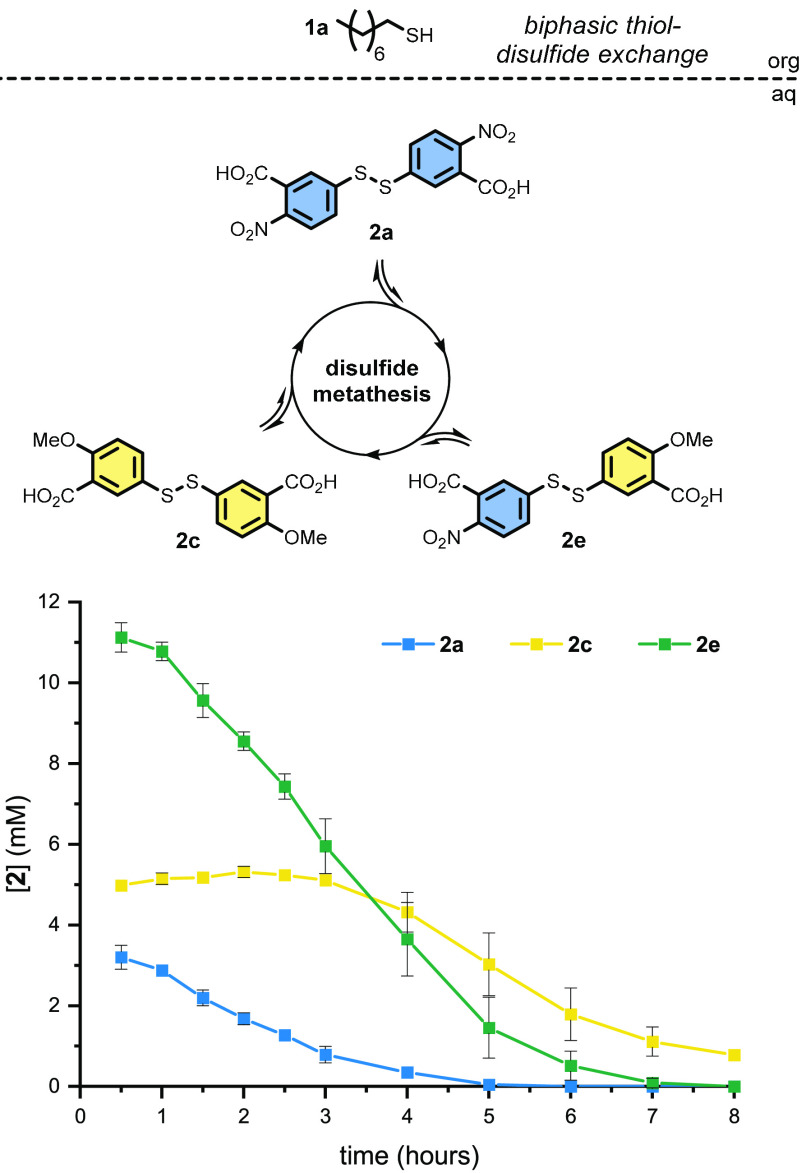
Disulfide Consumption in a Competition Reaction between **2a** and **2c** for thiol **1a** [**4aa**] and [**4ac**] data may be found in Scheme 4b. *n* = 3; error bars (where visible) represent the standard deviation.

Scheme 5 shows the reaction pathways available to disulfides **2a** and **2c** and their metathesis product **2e**. All three disulfides (**2a**, **2c**, and **2e**) can react with thiol **1a** directly to give either surfactant **4aa**, **4ac**, or both. The relative leaving group ability of the headgroups (**3**) causes any disulfides bearing electron-deficient headgroup **a** to be consumed the fastest, regardless of the product formed. In practice, this causes the production of an electron-rich surfactant (e.g., **4ac**) via heterodisulfides (e.g., **2e**) to be the dominant pathway in a competition setting. The disulfide consumption kinetics (Scheme 6) show that heterodisulfide **2e** is initially present at a high concentration and undergoes fast consumption. When **2a** is depleted, the less reactive **2c** then directly contributes to product formation.

Heterodisulfides **2d** and **2e** may be viewed as activated forms of parent disulfides **2b** and **2c**, and their presence allows rationalization of the kinetics and selectivity seen in competition experiments. For example, the initial, almost uniform, rates of **4aa**–**4ac** formation are due to a common leaving group (**3a**) in the most reactive disulfides **2a**, **2d**, and **2e** (see Scheme 4c). At longer times, the succession patterns in Scheme 4 show that **4ac** is the most kinetically stable surfactant as destruction is still governed by intrinsic electronic trends.

### Dynamic Covalent Activation

Dynamic covalent reactions have been widely studied in drug discovery and sensing,^45−48^ self-healing materials,^49,50^ catalyst discovery,^51,52^ and synthesis from dynamic mixtures.^53^ Exchangeable precursors have been studied in a replication setting, but with selectivity guided by auto- and cross-catalytic effects, rather than activation from dynamic exchange.^54,55^ The intermediate heterodisulfides, formed here via activating metathesis, promote the formation of products which are otherwise difficult to form. Through dynamic covalent substrate interactions, a particular reaction pathway is enabled, which to the best of our knowledge, is a unique selection mechanism for chemical replicators.

**Scheme 7:**
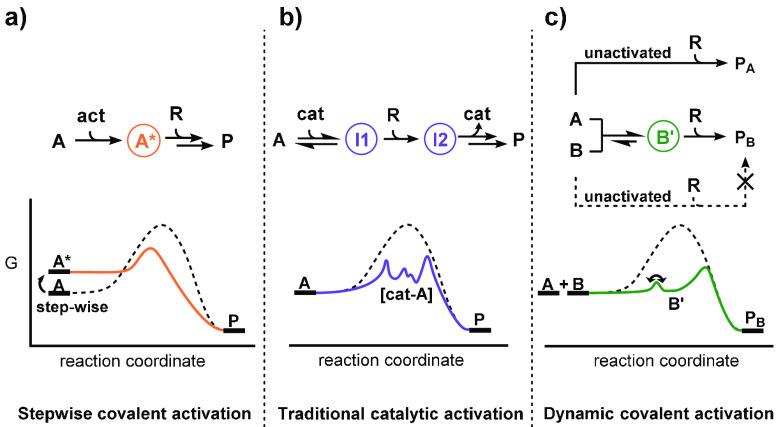
Comparison of Synthetic Activation Strategies and Illustrative Energy Landscapes Stepwise covalent activation, e.g., leaving group enhancement, relay ring-closing metathesis (RRCM). Traditional catalytic activation, e.g., Lewis acid catalysis, transition metal coordination. Dynamic covalent activation, e.g., disulfide metathesis. **act** = activating reagent, **R** = generic reagent, **cat** = catalyst, **P** = product.

More broadly, *dynamic covalent activation* may facilitate the formation of otherwise inaccessible small molecules and supramolecular assemblies. Activation by stepwise covalent modification, such as the transformation of a hydroxyl to a leaving group or relay ring-closing metathesis,^56^ relies upon discrete, irreversible chemical steps to access the desired product(s) (Scheme 7a). Many catalytic activation strategies, e.g., Lewis acid or transition metal catalysis, produce intermediates which undergo mechanistically different reactions from the unactivated transformation (Scheme 7b). In contrast, dynamic covalent activation elevates leaving group ability *in situ* through dynamic covalent reactions and requires no functional group interconversion or catalyst association. For example, Scheme 7c shows how product **P**_**B**_ may be accessed via intermediate **B′** on a pathway qualitatively similar to the unactivated route. Investigations into the generality of this concept are ongoing.

## Conclusions

We have found two mechanisms that can give rise to selection in systems of competing, metastable self-reproducing lipids. First, selection arises from differing chemical properties of the self-reproducing species themselves and their direct precursors. Here, the more sterically hindered surfactants form slower but are longer lived. A pattern reminiscent of ecological succession involving pioneer, follower, and mature species was observed.^57,58^

A second, emergent mechanism arises from network effects when precursors can inter-react. We demonstrate the preferential formation of products derived from less electrophilic disulfides due to dynamic covalent reactions that activate precursors. These dynamic processes gave rise to new intermediates that alter the reaction outcome, demonstrating that complex reaction mixtures can provide unexpected competitive advantages for specific species. This work suggests that increased species diversity can unlock new pathways in evolving systems and uncovered a dynamic activation mechanism that may be strategically useful in small molecule and supramolecular synthesis.^59,60^

## Supporting Information Available

Experimental procedures, compound synthesis and characterization, and supporting discussion (PDF)
