# Supplementary material for: Selection between Competing Self-Reproducing Lipids: Succession and Dynamic Activation
Source: JACS Au. 2021 Aug 16;1(9):1355–61. doi: 10.1021/jacsau.1c00138 (PMC8479773; doi:10.1021/jacsau.1c00138)
Supplement: Supplementary file 1 — au1c00138_si_001.pdf [file au1c00138_si_001.pdf]

Supporting Information

Selection between Competing Self-Reproducing Lipids: Succession and Dynamic Activation

Michael G. Howlett, Robert J. H. Scanes and Stephen P. Fletcher*

Chemistry Research Laboratory, University of Oxford, Oxford, UK

*Corresponding author: stephen.fletcher@chem.ox.ac.uk

Table of Contents

Materials and Methods 2
- General information 2
- Buffer preparation 2
- Biphasic reaction setup 2
- Reaction sampling 3
- Analytical UPLC gradients 3
- UPLC calibration 7

Supplementary kinetic data 11

Synthetic procedures 21

NMR spectra 26

Additional surfactant characterisation 41
- Ring tensiometry measurements 41
- Dynamic light scattering (DLS) measurements 42

References 46

Materials and Methods

General information

^{1}H NMR and ^{13}C NMR spectra were recorded on a Bruker Avance III HD 400 MHz spectrometer using $CDCl_3$, DMSO-d_6 and acetone-d_6 as solvent. Chemical shifts (δ) are expressed in parts per million (ppm) with respect to residual solvent signals and coupling constants are quoted in hertz (Hz). Multiplets are described as s (singlet), d (doublet), t (triplet), q (quartet), p (pentet), sextet and m (multiplet). Melting points were recorded using purified samples on a Leica Galen III microscope in conjunction with a heated-stage and Testo 720 thermocouple. Infrared spectra were recorded using neat, purified samples on a Bruker Tensor 27 FT-IR spectrometer. High-resolution mass spectra were recorded on a Thermo Exactive Orbitrap mass spectrometer equipped with a Waters Acquity LC system under conditions of electrospray ionization (ESI) by the internal service within the Department of Chemistry. Reagents were obtained from commercial sources where indicated and were used directly as supplied. All aqueous samples, standard solutions and buffers used ultrapure Milli-Q water and/or HPLC grade organic solvents. TLC analyses were performed using Merck Kiesegel 60 F_{254} 0.25 mm precoated silica plates and preparative TLC performed using 200 x 200 mm 60 Å Analtech precoated glass backed plates.

Buffer preparation

Buffer solutions were prepared by dissolving the required mass of 4-(2-hydroxyethyl)-1-piperazineethanesulfonic acid (HEPES) in approximately 90% of the final volume Milli-Q water. The pH and volume were adjusted by addition of a saturated aqueous solution of potassium hydroxide, as measured with a Jenway 3510 benchtop pH meter. Final pH of the buffer was measured at room temperature, after stirring and equilibration of the solution, to an accuracy of ± 0.01 pH units. Buffer solutions were regularly prepared fresh and stored in the dark.

Biphasic reaction setup

Reactions were performed in 10 mL round bottom flasks using Fisherbrand oval 20 x 10 mm PTFE stir bars and were stirred using IKA RCT basic stir plates. At room temperature, once all the aqueous components were dissolved, the *appropriate thiol* (**1**) (1.0 or 8.0 eq.)

was carefully layered onto an aqueous solution of the *appropriate disulfide* **2** (25 mM, 1.00 mmol, 1.0 eq.) in 4-(2-hydroxyethyl)-1-piperazineethanesulfonic acid (HEPES) aqueous buffer (pH 8.00, 4.0 mL) whilst stirring at 100 rpm. For reactions involving multiple aryl disulfides, the total initial concentration $[ArSSAr]_0$ was set as 25.0 mM and the aqueous solution was pre-stirred for 10 minutes prior to **1** addition. For reactions involving disulfide **2c**, experiments were set up under an inert atmosphere: buffer solutions and vessels were degassed by sparging with argon for a minimum of 1 hour and were sealed with a rubber septum and a positive argon pressure maintained, including during the sampling of aliquots.

Reaction sampling

All UV-active reaction species of interest were monitored and quantified by the UPLC analysis of aliquots. Reactions were sampled by withdrawing precisely 25 µL aliquots from the lower aqueous layer by microsyringe (Hamilton 25 µL Gastight) and quenching with precisely 1.0 mL of quench solution (maleimide, 6 mg/mL Milli-Q water) before injection into the UPLC instrument for quantification. Kinetics experiments were run in triplicate under identical conditions and occurred simultaneously, unless otherwise stated.

Analytical UPLC gradients

Kinetic analysis of reactions was performed using a reverse phase Acquity UPLC BEH C18 column (130 Å, 1.7 µm, 2.1 mm × 50 mm) on a Waters Acquity Ultra-Performance liquid chromatography (UPLC) H-Class system with photodiode array (PDA) detector. Data processing was conducted with the Waters Empower 3 software. Peak areas were integrated manually at a wavelength of 240.0 nm, with peak identity being confirmed by both retention time and UV spectra. Concentrations were quantified using the calibration constants found in the calibration plots below. Calibration of the reaction components was achieved by multiple injections of accurate solutions to give a linear fit. Heteroaryl disulfides **2d-f** were calibrated by taking the mean of the absorbances of the respective homoaryl disulfides from which they originate.

Mobile phases used in all methods are as follows: H_2O, MeCN, MeOH and 2% aqueous TFA. A multipurpose 5 minute gradient was used to monitor reactions containing only one aryl disulfide whereas further optimization was required for separating and quantifying more complex mixtures. The solvent gradients used are shown in the following tables, as are the retention times of species relevant to that gradient.

Table S1. Gradient 1.[a]

Time /mins	Flow rate $/mLmin^{-1}$	H_2O /%	MeCN /%	5% aq. TFA /%	Curve
0.00	0.600	93.0	2.0	5.0	Initial
0.25	0.600	93.0	2.0	5.0	6
4.00	0.600	2.0	93.0	5.0	6
4.40	0.600	2.0	93.0	5.0	6
4.50	0.600	93.0	2.0	5.0	10
5.00	0.600	93.0	2.0	5.0	10

[a] General purpose gradient that was used to monitor reactions containing one aryl disulfide.

Table S2. Retention time of relevant peaks for a typical elution using Gradient 1.

Compound	Retention time /mins
Aryl thiols **3**	1.84-1.90
Aryl disulfides **2**	2.63-2.74
Surfactant products (mixed disulfides) **4**	4.01-4.39

Table S3. Gradient 2.[a]

Time /mins	Flow rate /mLmin^{-1}	H_2O /%	MeCN /%	5% aq. TFA /%	Curve
0.00	0.250	88.0	2.0	10.0	Initial
0.25	0.250	88.0	2.0	10.0	6
0.75	0.250	58.0	32.0	10.0	6
8.50	0.250	58.0	32.0	10.0	6
8.60	0.250	2.0	93.0	5.0	6
9.90	0.250	2.0	93.0	5.0	6
10.00	0.250	88.0	2.0	10.0	10
10.50	0.250	88.0	2.0	10.0	10

[a] Specific gradient for monitoring mixtures of aryl disulfides, giving a distinct retention time for each one.

Table S4. Retention time of relevant peaks for a typical elution using Gradient 2.

Compound	Retention time /mins
homoaryl disulfide **2c**	5.45
heteroaryl disulfide **2f**	6.01
homoaryl disulfide **2a**	6.22
heteroaryl disulfide **2e**	6.49
heteroaryl disulfide **2d**	7.12
homoaryl disulfide **2b**	7.97

Table S5. Gradient 3a.[a]

Time /mins	Flow rate /mLmin^{-1}	H_2O /%	MeCN /%	5% aq. TFA /%	Curve
0.00	0.600	93.0	2.0	5.0	Initial
0.25	0.600	93.0	2.0	5.0	6
0.50	0.600	90.0	5.0	5.0	6
3.75	0.600	68.0	27.0	5.0	6
4.00	0.600	2.0	93.0	5.0	6
4.40	0.600	2.0	93.0	5.0	6
4.50	0.600	93.0	2.0	5.0	11
5.00	0.600	93.0	2.0	5.0	11

[a] Specific gradient for monitoring mixtures of aryl thiols, giving a distinct retention time for each one.

Table S6. Retention time of relevant peaks for a typical elution using Gradient 3a.

Compound	Retention time /mins
aryl thiol **3c**	2.65
aryl thiol **3a**	2.83
aryl thiol **3b**	2.89

Table S7. Gradient 3b.[a]

Time /mins	Flow rate /mLmin^{-1}	H_2O /%	MeCN /%	5% aq. TFA /%	Curve
0.00	0.450	93.0	2.0	5.0	Initial
0.25	0.450	93.0	2.0	5.0	6
0.50	0.450	90.0	5.0	5.0	6
3.75	0.450	72.0	23.0	5.0	6
4.00	0.450	2.0	93.0	5.0	6
4.40	0.450	2.0	93.0	5.0	6
4.50	0.450	93.0	2.0	5.0	11
5.00	0.450	93.0	2.0	5.0	11

[a] Alternative gradient for monitoring mixtures of aryl thiols, giving a distinct retention time for each one.

Table S8. Retention time of relevant peaks for a typical elution using Gradient 3b.

Compound	Retention time /mins
aryl thiol **3c**	3.35
aryl thiol **3a**	3.55
aryl thiol **3b**	3.65

Table S9. Gradient 4a.[a]

Time /mins	Flow rate /mLmin^{-1}	H_2O /%	MeCN /%	MeOH /%	5% aq. TFA /%	Curve
0.00	0.600	78.0	2.0	15.0	5.0	Initial
0.25	0.600	78.0	2.0	15.0	5.0	6
0.75	0.600	23.0	57.0	15.0	5.0	6
3.50	0.600	19.0	61.0	15.0	5.0	5
4.00	0.600	19.0	61.0	15.0	5.0	6
4.10	0.600	1.0	93.0	1.0	5.0	6
4.80	0.600	78.0	2.0	15.0	5.0	11
5.00	0.600	78.0	2.0	15.0	5.0	10

[a] Specific gradient for monitoring mixtures of surfactant products (mixed disulfides). When used in combination with Gradient 4b, a distinct, quantifiable peak was obtained for each product.

Table S10. Retention time of relevant peaks for a typical elution using Gradient 4a.

Compound	Retention time /mins[a]
**4ac**	2.58
**4aa**	2.63
**4bc**	2.82
**4ca**	2.91
**4ab**	2.91
**4cc**	3.01
**4ca**	3.14
**4bb**	3.23
**4cb**	3.50

[a] Well resolved, quantifiable peaks for this gradient are underlined. See Gradient 4b for remaining peaks.

Table S11. Gradient 4b.[a]

Time /mins	Flow rate /mLmin^{-1}	H_2O /%	MeCN /%	MeOH /%	5% aq. TFA /%	Curve
0.00	0.450	83.0	2.0	10.0	5.0	Initial
0.25	0.450	83.0	2.0	10.0	5.0	6
0.75	0.450	34.0	51.0	10.0	5.0	6
3.50	0.450	30.0	55.0	10.0	5.0	5
6.40	0.450	24.0	61.0	10.0	5.0	6
6.50	0.450	1.0	93.0	1.0	5.0	6
7.20	0.450	83.0	2.0	10.0	5.0	11
7.50	0.450	83.0	2.0	10.0	5.0	10

[a] Specific gradient for monitoring mixtures of surfactant products (mixed disulfides). When used in combination with Gradient 4a, a distinct, quantifiable peak was obtained for each product.

Table S12. Retention time of relevant peaks for a typical elution using Gradient 4b.

Compound	Retention time /mins[a]
**4ac**	4.93
**4aa**	5.19
**4bc**	5.63
**4ab**	5.80
**4ba**	6.00
**4cc**	6.17
**4ca**	6.40
**4bb**	6.40
**4cb**	6.62

[a] Well resolved, quantifiable peaks for this gradient are underlined. See Gradient 4a for remaining peaks.

UPLC calibration

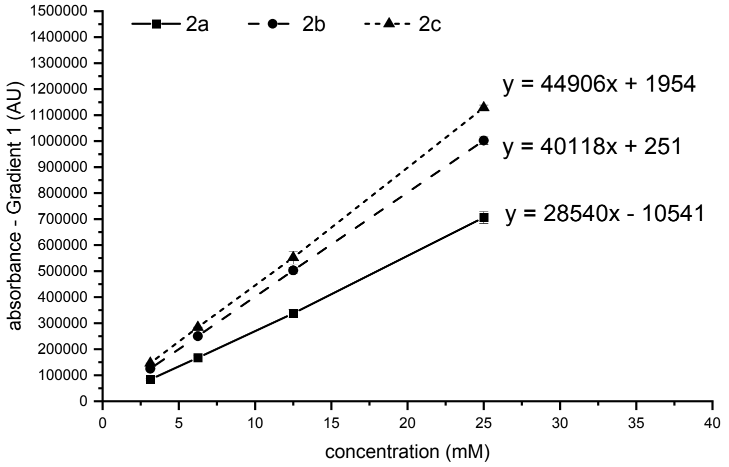

Figure S1 Calibration plot for compounds **2a**-**c** for UPLC analysis by Gradient 1. n = 3, error bars (where visible) represent the standard deviation

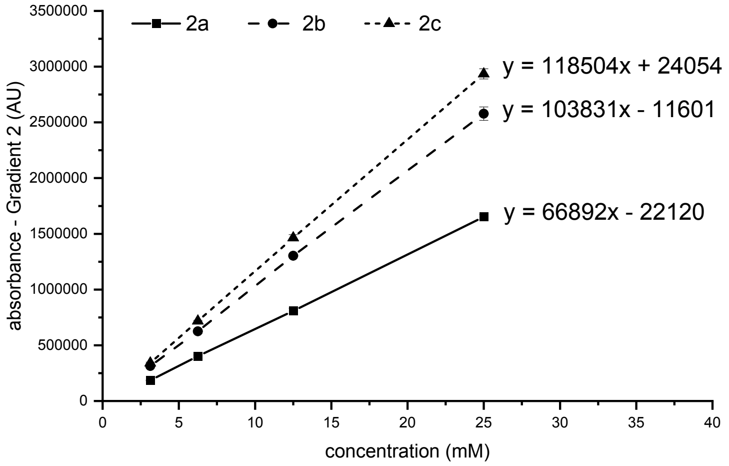

Figure S2 Calibration plot for compounds **2a**-**c** for UPLC analysis by Gradient 2. n = 3, error bars (where visible) represent the standard deviation

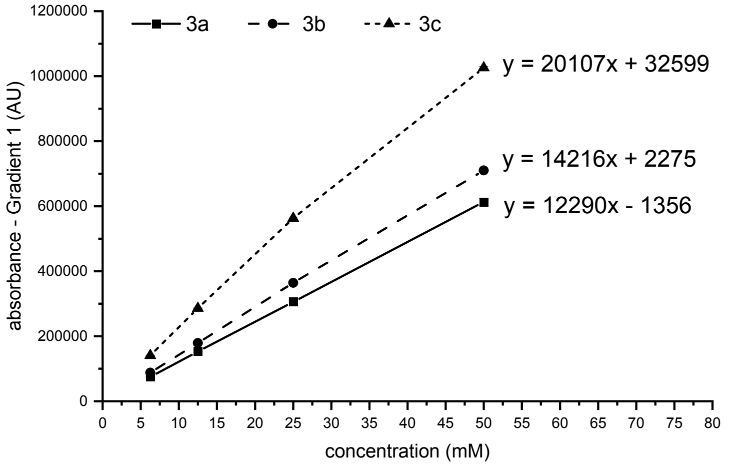

Figure S3 Calibration plot for compounds **3a**-**c** for UPLC analysis by Gradient 1. n = 3, error bars (where visible) represent the standard deviation

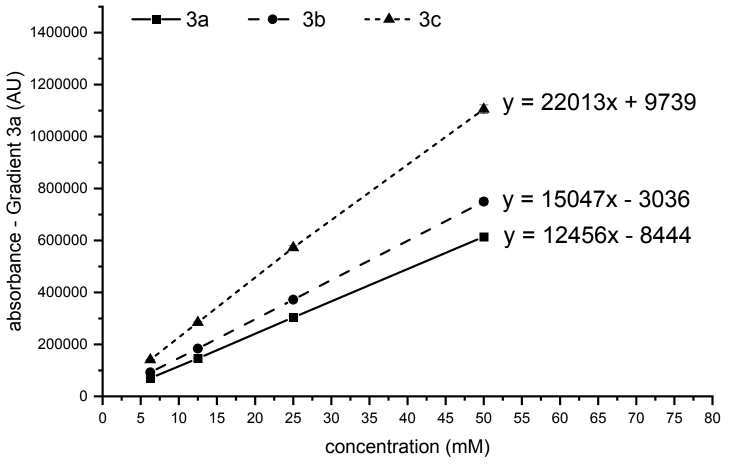

Figure S4 Calibration plot for compounds **3a**-**c** for UPLC analysis by Gradient 3a. n = 3, error bars (where visible) represent the standard deviation

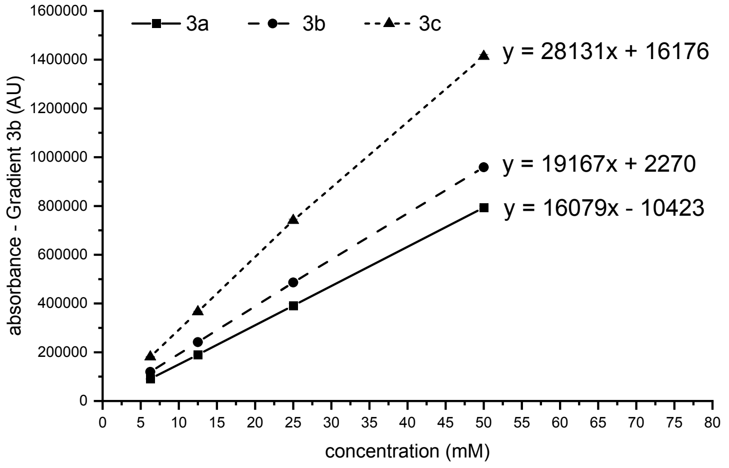

Figure S5 Calibration plot for compounds **3a**-**c** for UPLC analysis by Gradient 3b. n = 3, error bars (where visible) represent the standard deviation

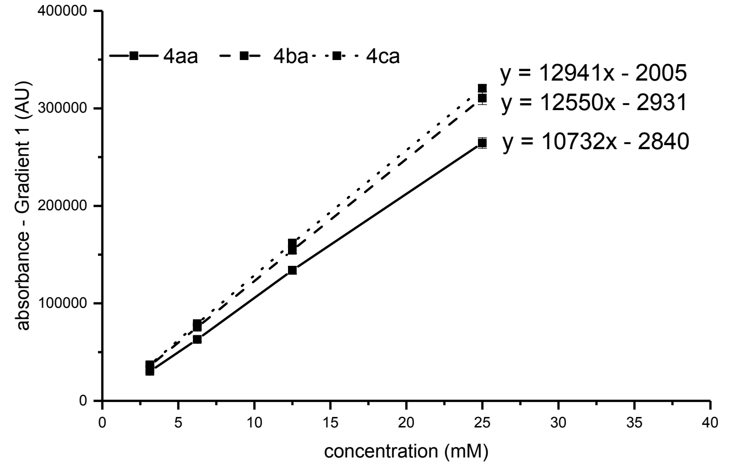

Figure S6 Calibration plot for compounds **4aa**-**ca** for UPLC analysis by Gradient 1. n = 3, error bars (where visible) represent the standard deviation

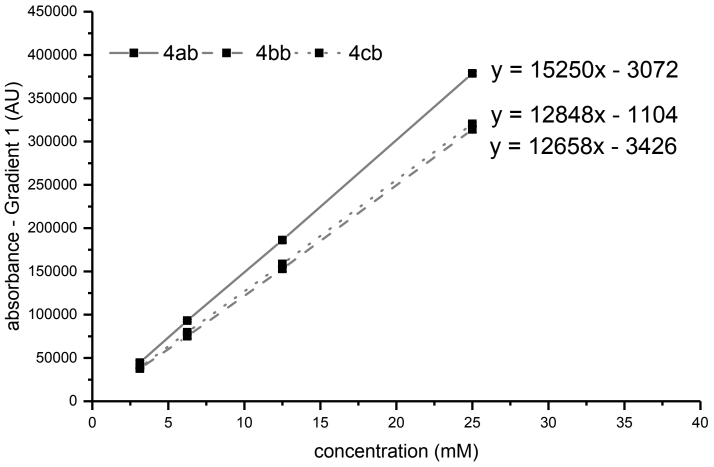

Figure S7 Calibration plot for compounds **4ab**-**cb** for UPLC analysis by Gradient 1. n = 3, error bars (where visible) represent the standard deviation

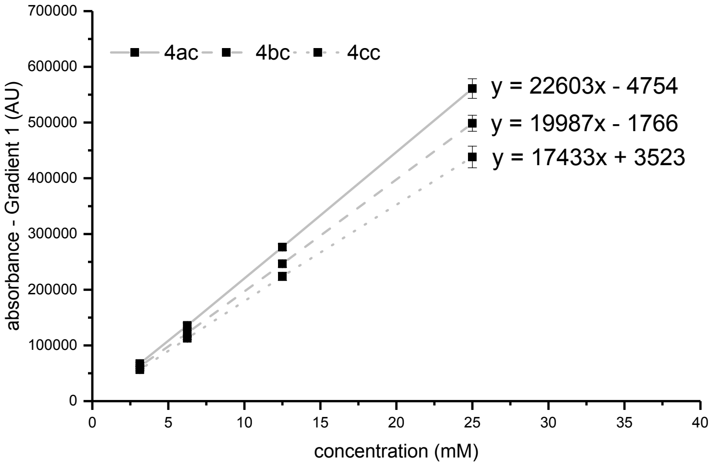

Figure S8 Calibration plot for compounds **4ac**-**cc** for UPLC analysis by Gradient 1. n = 3, error bars (where visible) represent the standard deviation

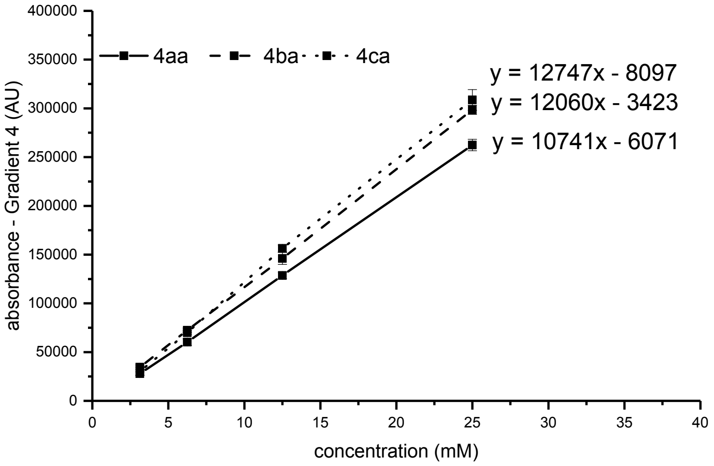

Figure S9 Calibration plot for compounds **4aa**-**ca** for UPLC analysis by Gradient 4. n = 3, error bars (where visible) represent the standard deviation

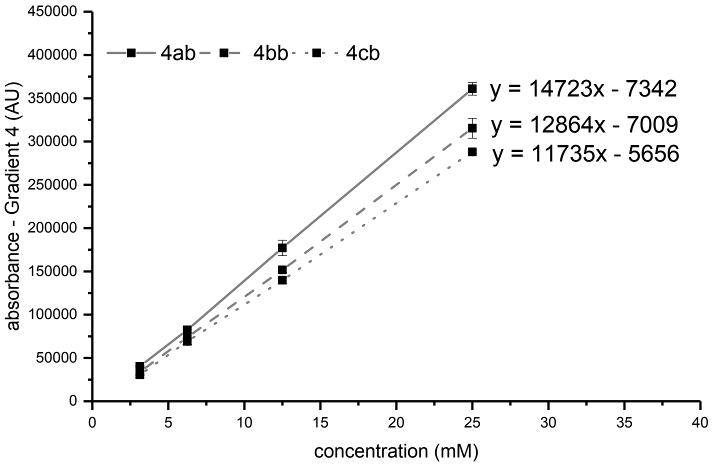

Figure S10 Calibration plot for compounds **4ab**-**cb** for UPLC analysis by Gradient 4. n = 3, error bars (where visible) represent the standard deviation

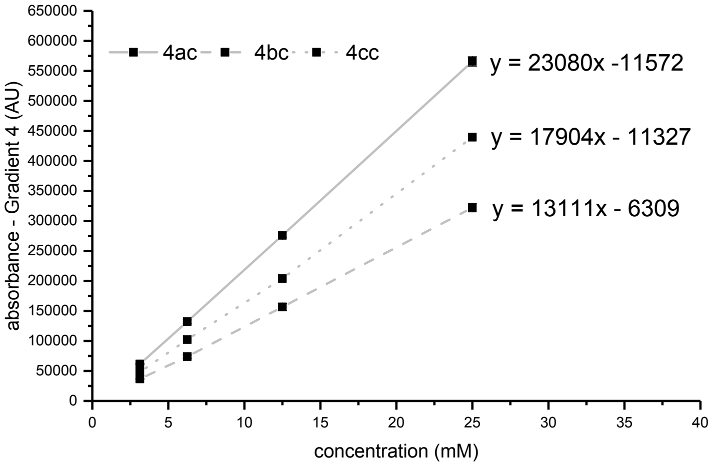

Figure S11 Calibration plot for compounds **4ac**-**cc** for UPLC analysis by Gradient 4. n = 3, error bars (where visible) represent the standard deviation

Supplementary kinetic data

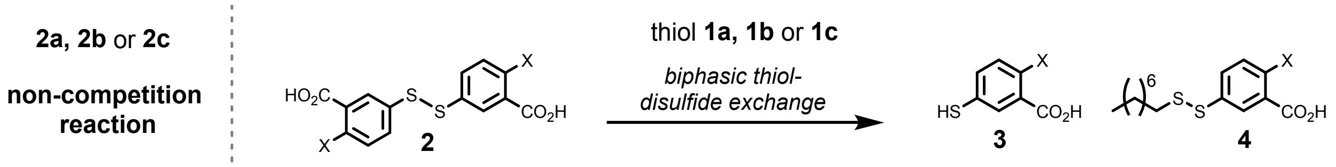

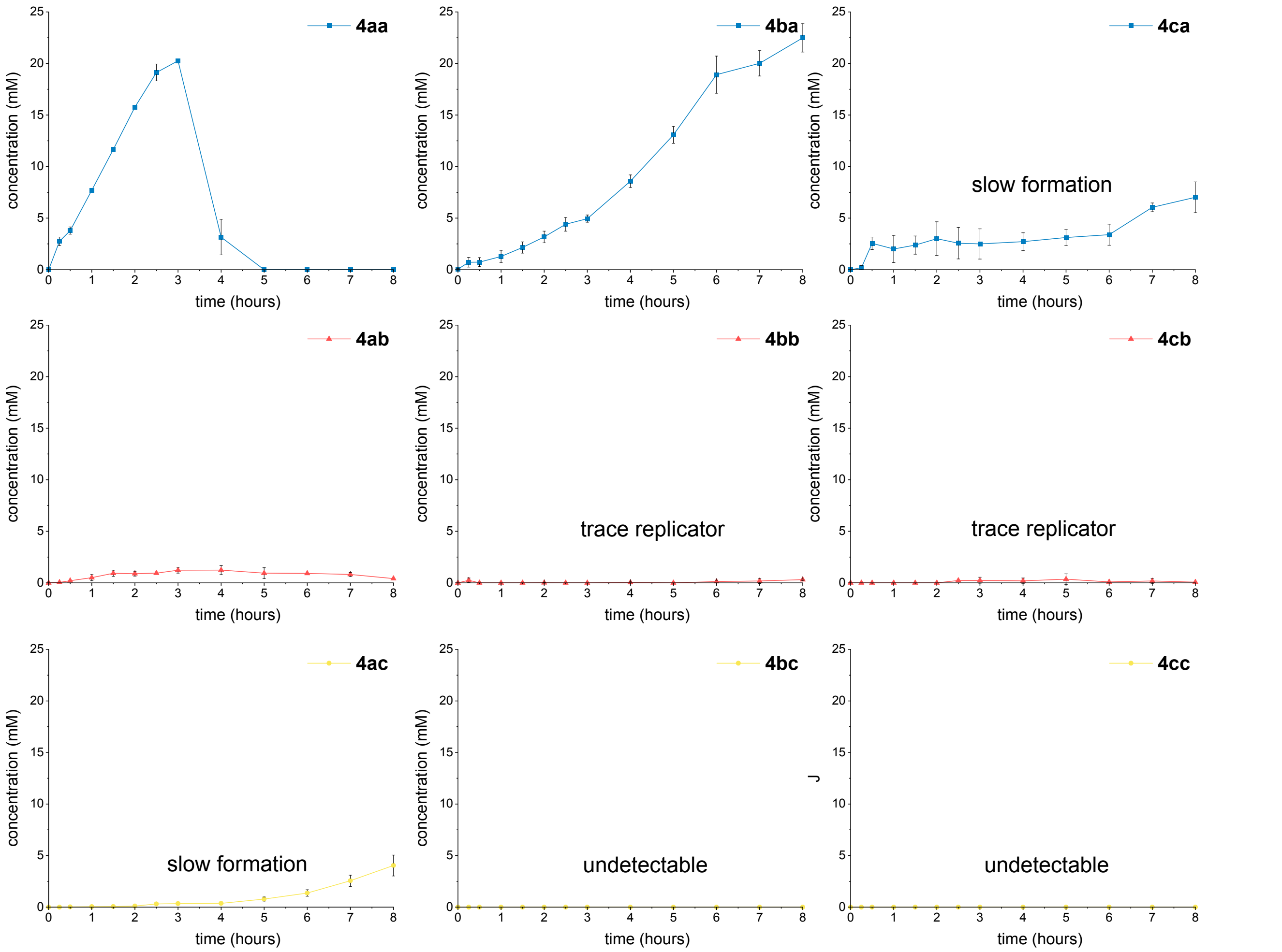

Replicators:

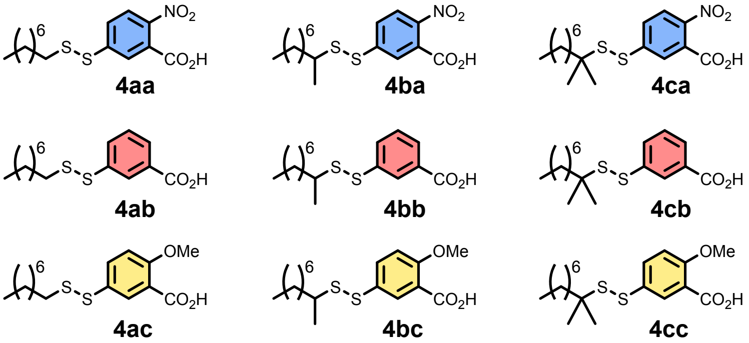

*Figure S12 Summary of non-competition experiments for all products **4** showing relative formation and destruction characteristics (i.e. relative reactivity in biphasic thiol-disulfide exchange) for a single thiol (**1**) and disulfide (**2**) pair. [**2a-c**] and [**3a-c**] data shown in full below. HEPES buffer (pH 8.0), r.t., 100 rpm, 8.0 eq. thiol (**1a**, **1b** or **1c**) n = 3, error bars (where visible) represent the standard deviation*

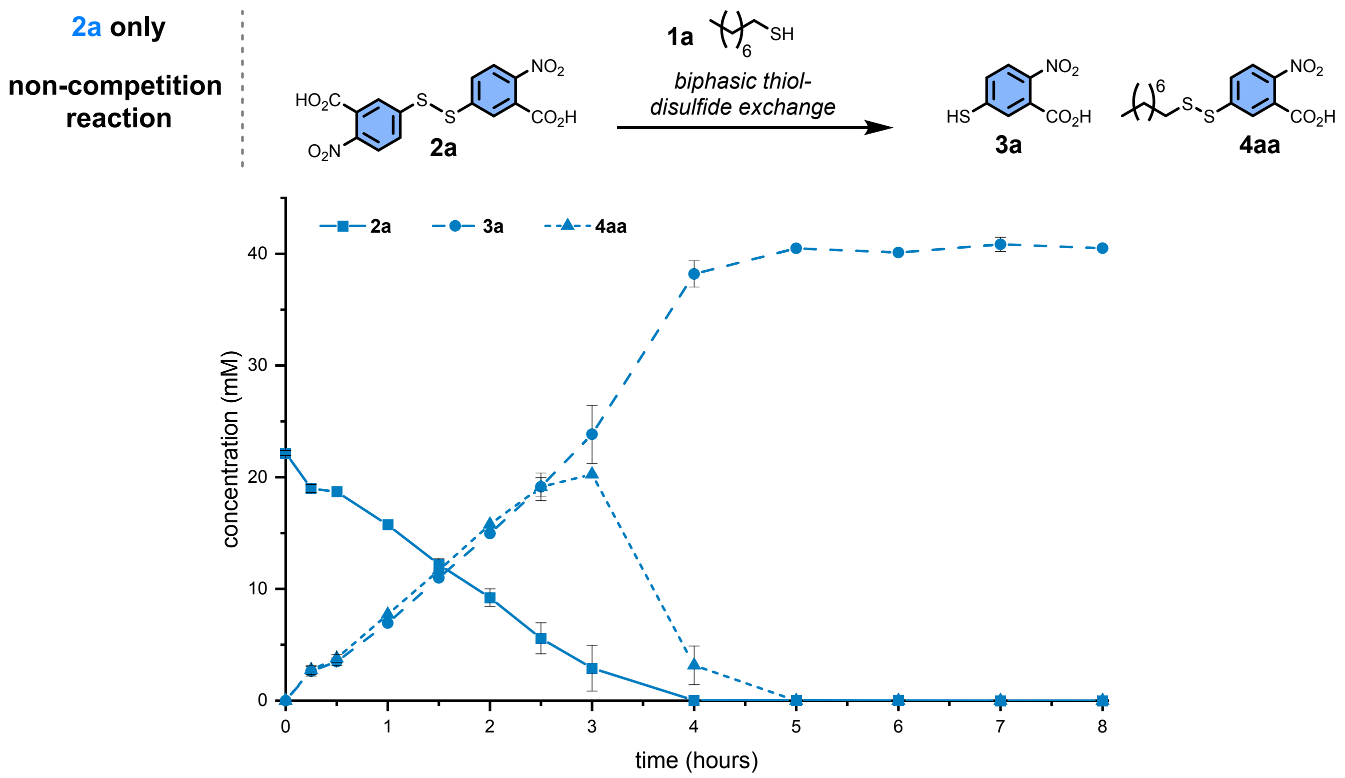

*Figure S13 Non-competition biphasic thiol-disulfide exchange between **2a** and thiol **1a**. HEPES buffer (pH 8.0), r.t., 100 rpm, 8.0 eq. thiol **1a** n = 3, error bars (where visible) represent the standard deviation*

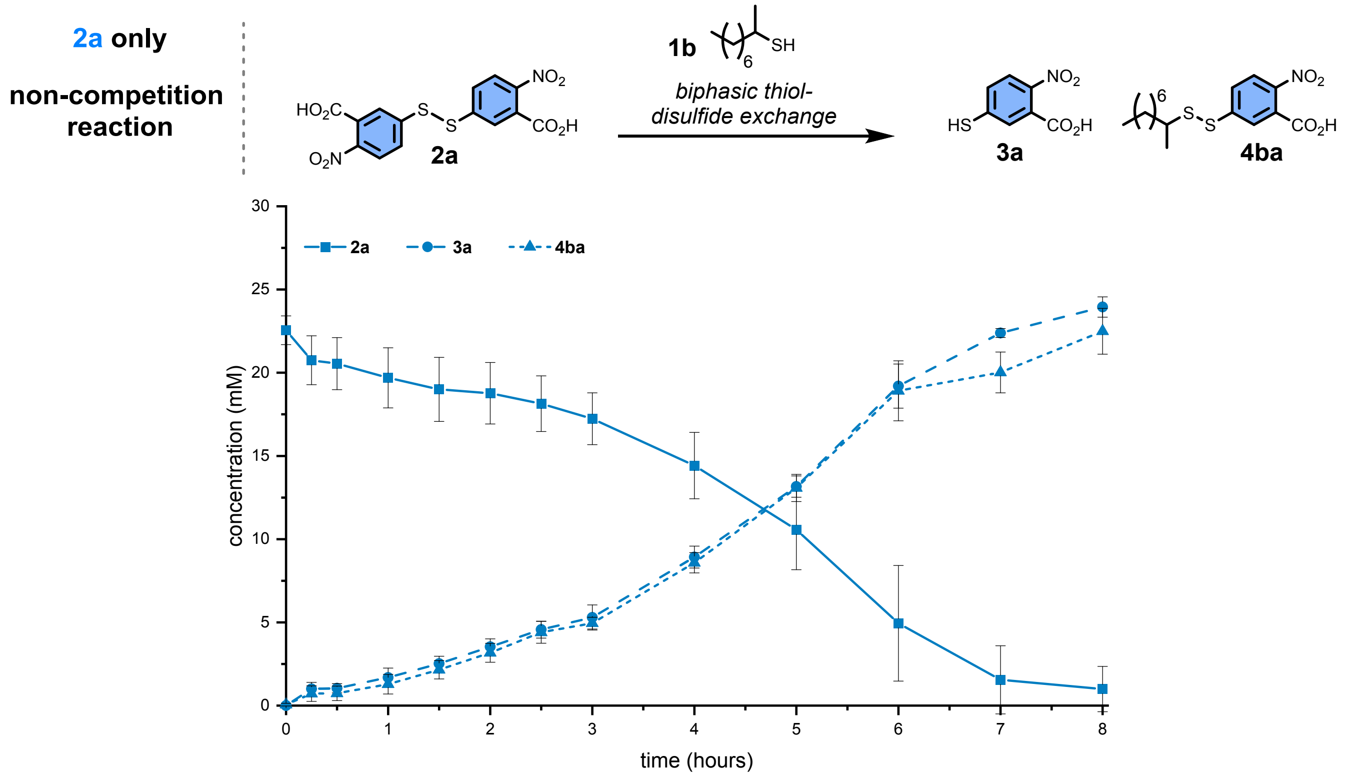

*Figure S14 Non-competition biphasic thiol-disulfide exchange between **2a** and thiol **1b**. HEPES buffer (pH 8.0), r.t., 100 rpm, 8.0 eq. thiol **1b** n = 3, error bars (where visible) represent the standard deviation*

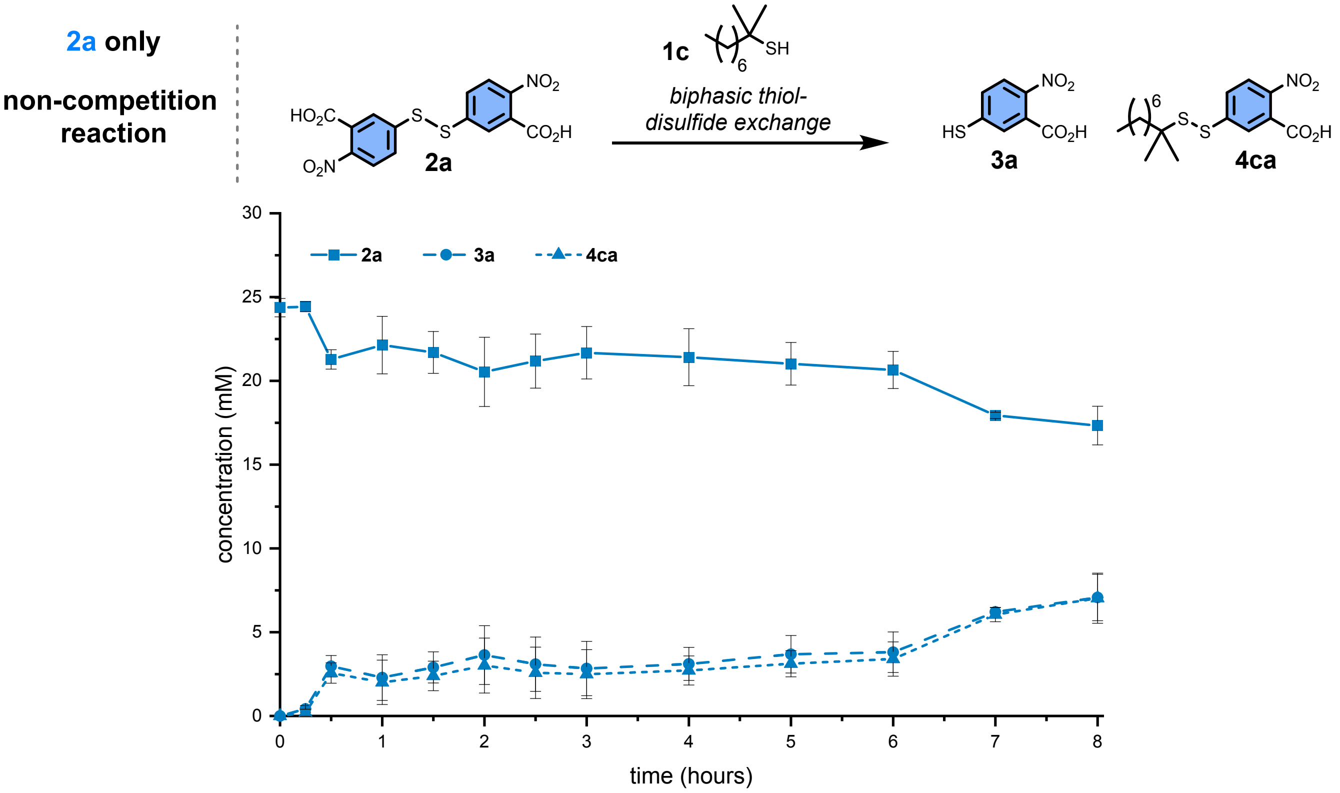

*Figure S15 Non-competition biphasic thiol-disulfide exchange between **2a** and thiol **1c**. HEPES buffer (pH 8.0), r.t., 100 rpm, 8.0 eq. thiol **1c** n = 3, error bars (where visible) represent the standard deviation*

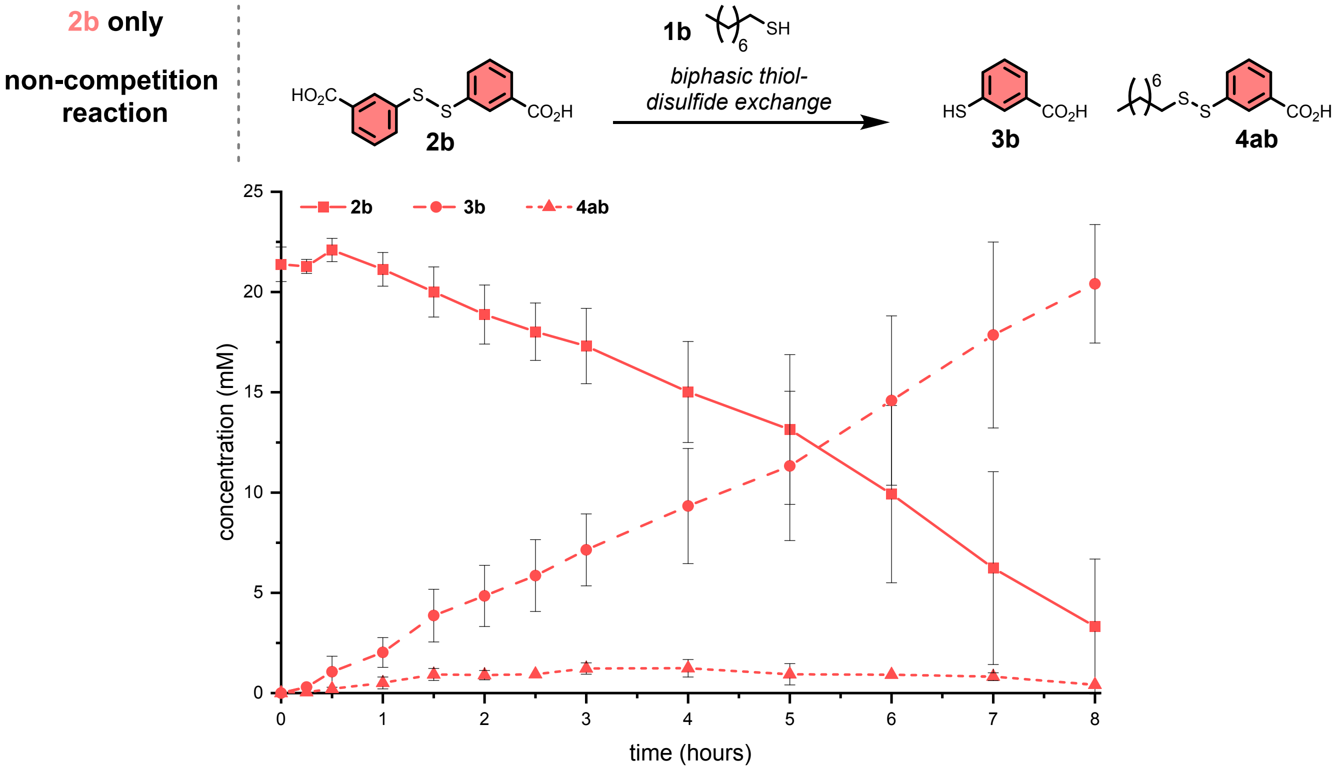

*Figure S16 Non-competition biphasic thiol-disulfide exchange between **2b** and thiol **1a**. HEPES buffer (pH 8.0), r.t., 100 rpm, 8.0 eq. thiol **1a** n = 3, error bars (where visible) represent the standard deviation*

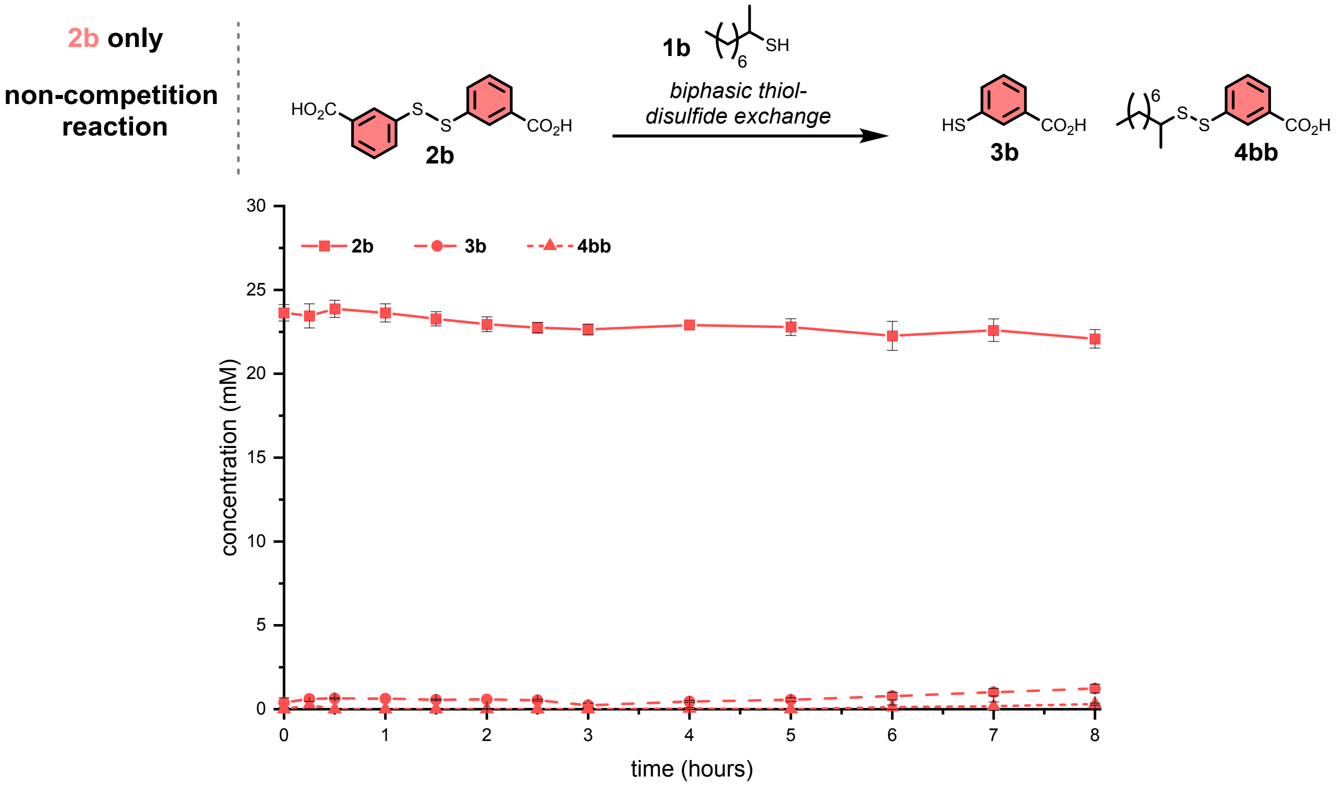

*Figure S17 Non-competition biphasic thiol-disulfide exchange between **2b** and thiol **1b**. HEPES buffer (pH 8.0), r.t., 100 rpm, 8.0 eq. thiol **1b** n = 3, error bars (where visible) represent the standard deviation*

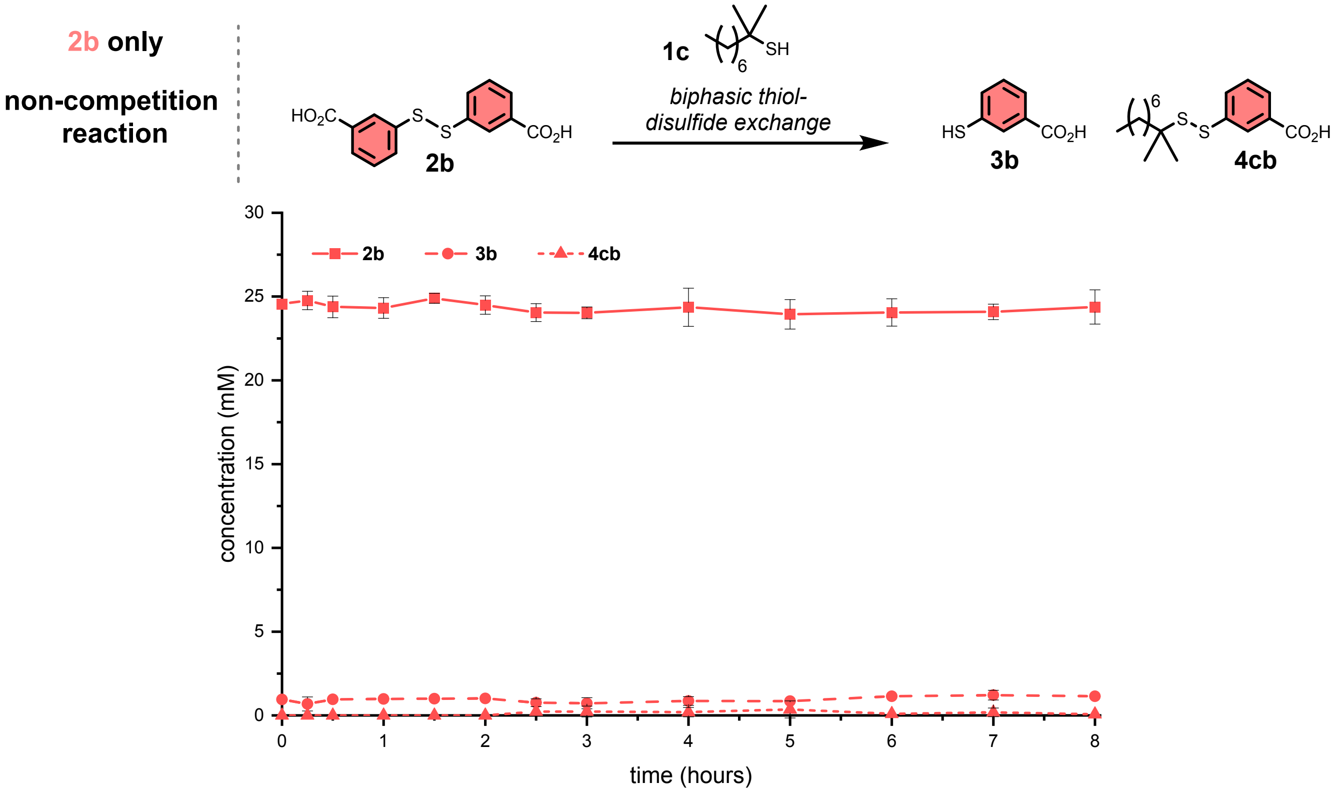

*Figure S18 Non-competition biphasic thiol-disulfide exchange between **2b** and thiol **1c**. HEPES buffer (pH 8.0), r.t., 100 rpm, 8.0 eq. thiol **1c** n = 3, error bars (where visible) represent the standard deviation*

2c only

non-competition reaction

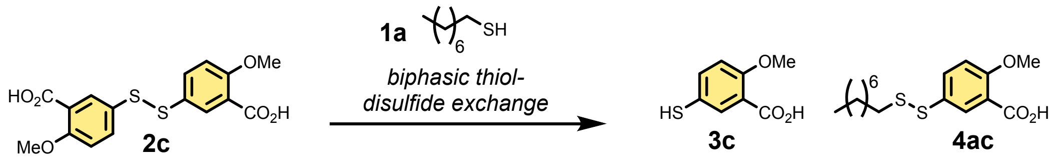

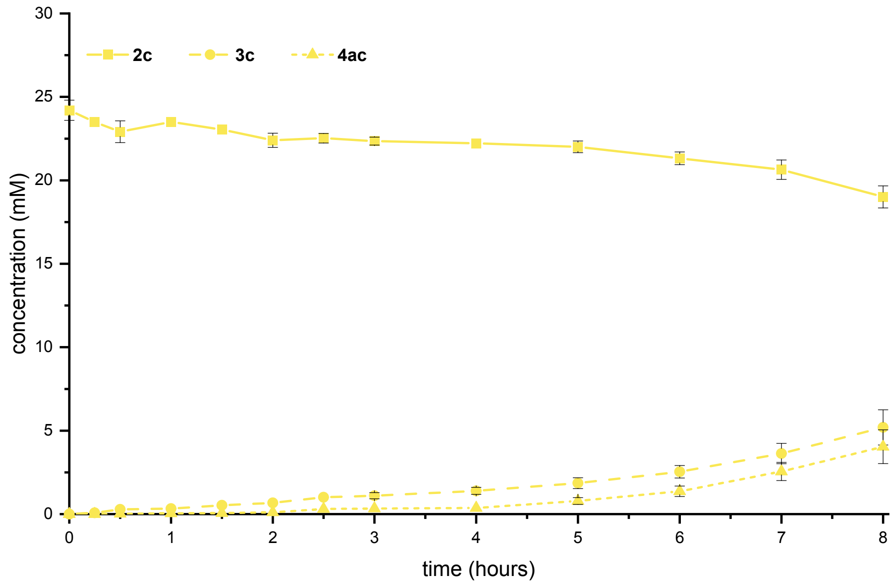

*Figure S19 Non-competition biphasic thiol-disulfide exchange between **2c** and thiol **1a**. HEPES buffer (pH 8.0), r.t., 100 rpm, 8.0 eq. thiol **1a** n = 3, error bars (where visible) represent the standard deviation*

2c only

non-competition reaction

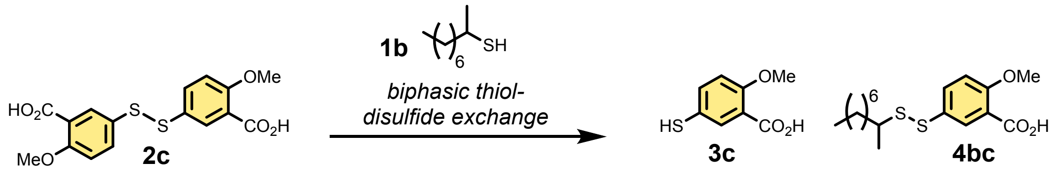

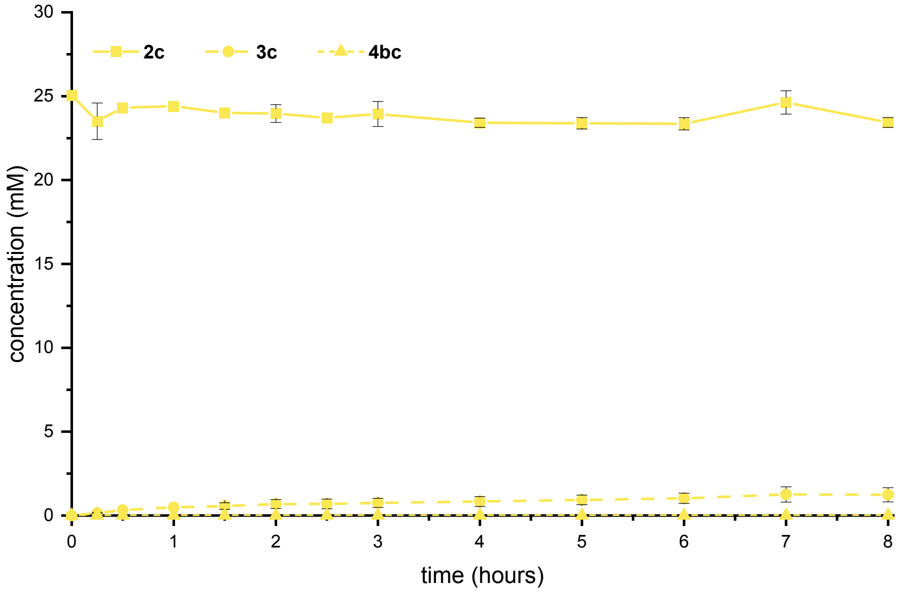

*Figure S20 Non-competition biphasic thiol-disulfide exchange between **2c** and thiol **1b**. HEPES buffer (pH 8.0), r.t., 100 rpm, 8.0 eq. thiol **1b** n = 3, error bars (where visible) represent the standard deviation*

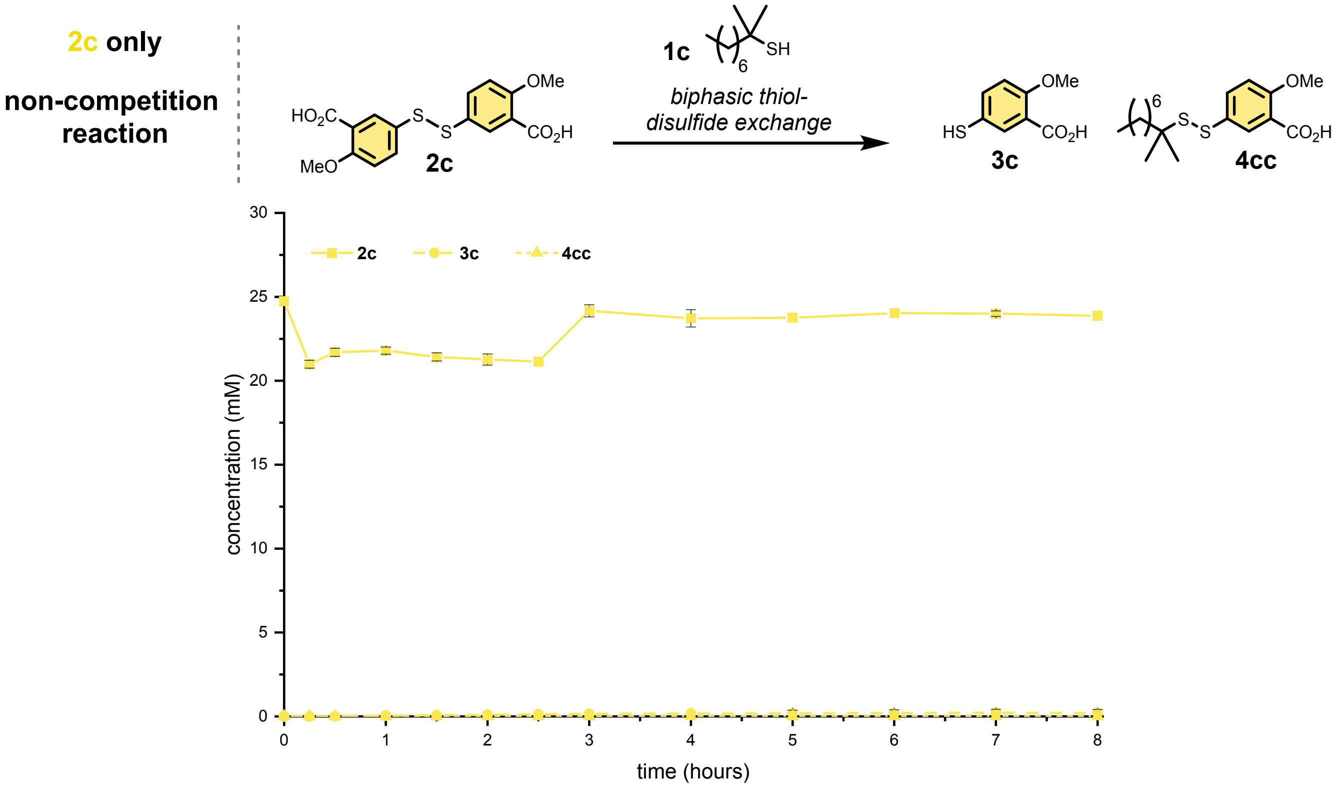

Figure S21 Non-competition biphasic thiol-disulfide exchange between ***2c*** *and thiol* ***1c****. HEPES buffer (pH 8.0), r.t., 100 rpm, 8.0 eq. thiol* ***1c*** *n = 3, error bars (where visible) represent the standard deviation*

Equilibration experiments

Disulfide metathesis equilibration was conducted in the same manner as the competition experiments described above using separate premade 15 mM stock solutions containing only each disulfide **2** in degassed aqueous HEPES buffer (pH 8.0). Metathesis was initiated by combining these solutions in equal volumes to give a mixture where $[\mathbf{2}]_{tot}$ = 15 mM. Data presented from this procedure represents a single experiment for each combination of disulfides.

These experiments showed disulfides **2a-f** coming to equilibrium over the course of approximately an hour (in the absence of other species). We propose this is catalysed by trace aryl thiol **3** produced by disulfide hydrolysis in the alkaline buffer. No metathesis was observed over a period of 24 hours in samples treated with a maleimide quench, consistent with thiol catalysis.

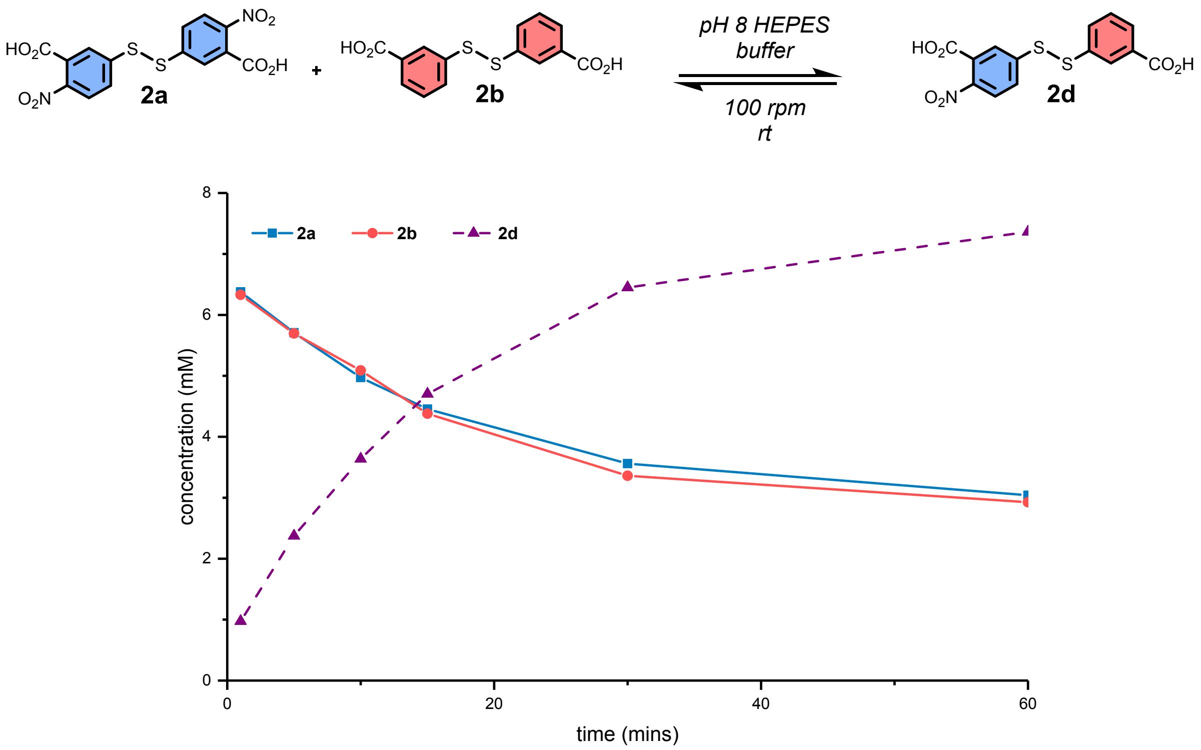

*Figure S22 Disulfide metathesis between **2a** and **2c**. HEPES buffer (pH 8.0), r.t., 100 rpm*

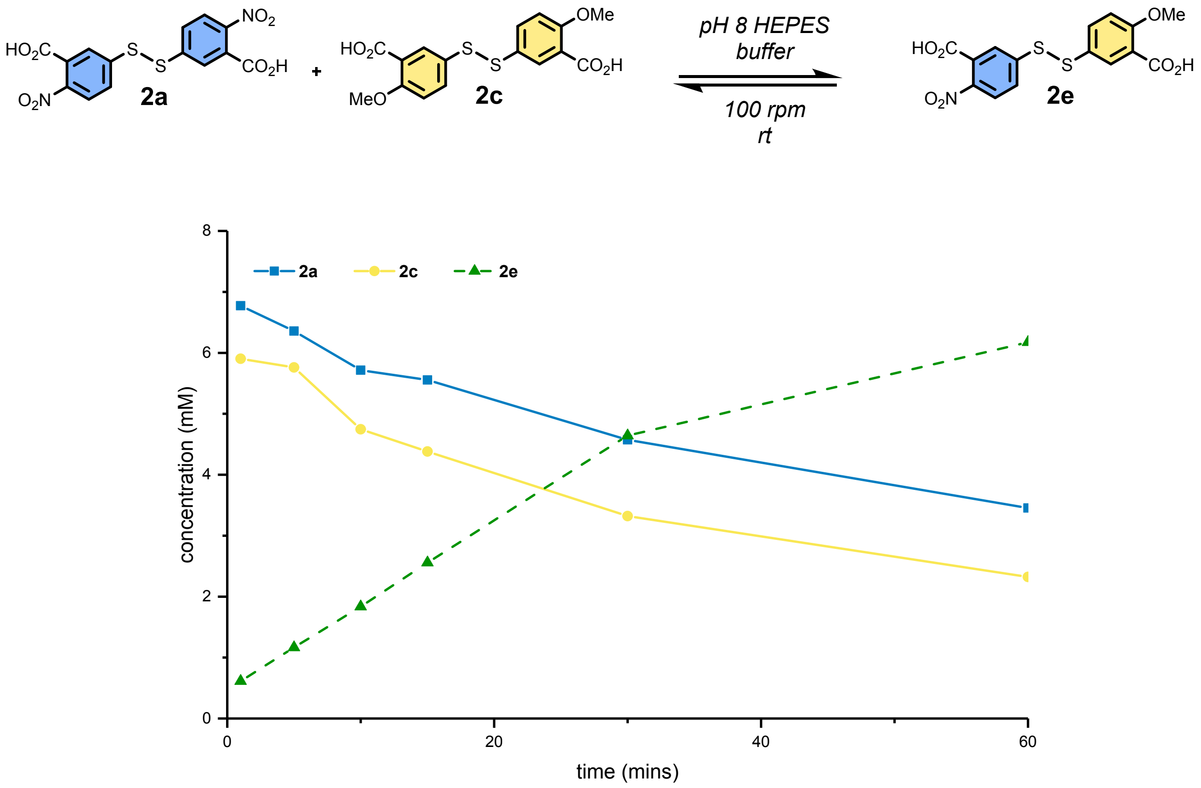

*Figure S23 Disulfide metathesis between **2a** and **2c**. HEPES buffer (pH 8.0), r.t., 100 rpm*

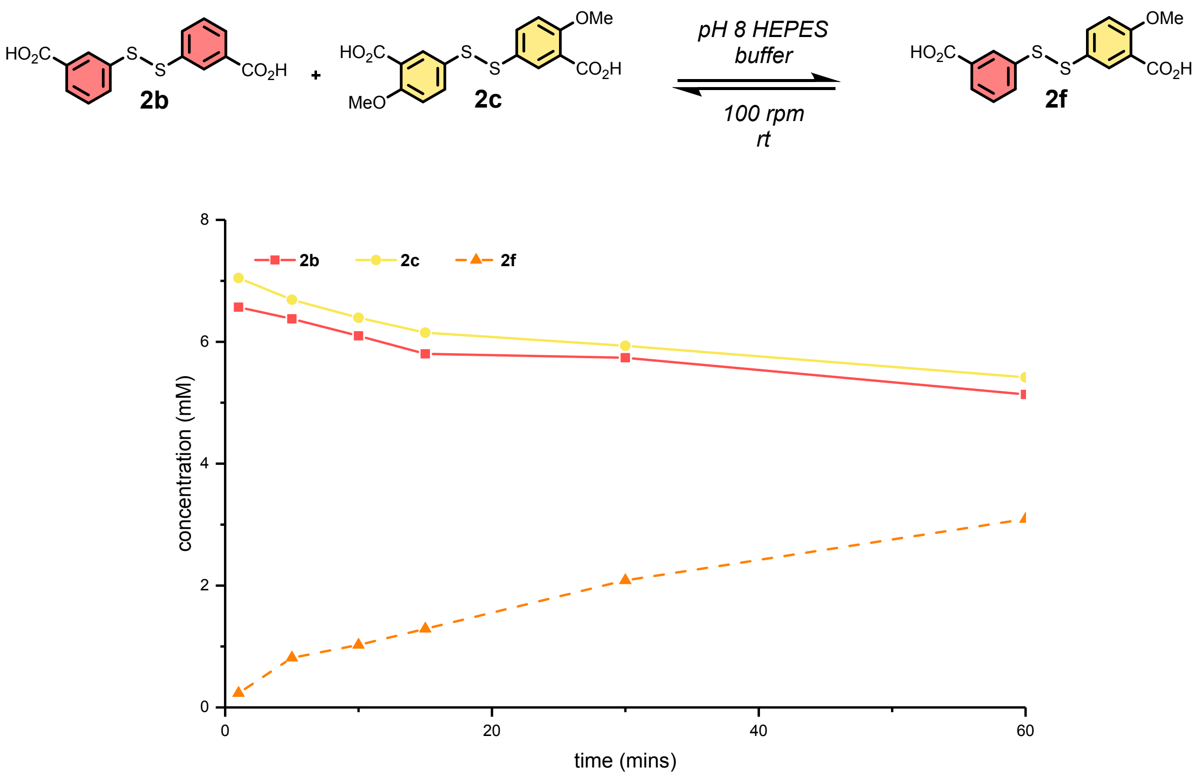

Figure S24 Disulfide metathesis between ***2b*** *and* ***2c****. HEPES buffer (pH 8.0), r.t., 100 rpm*

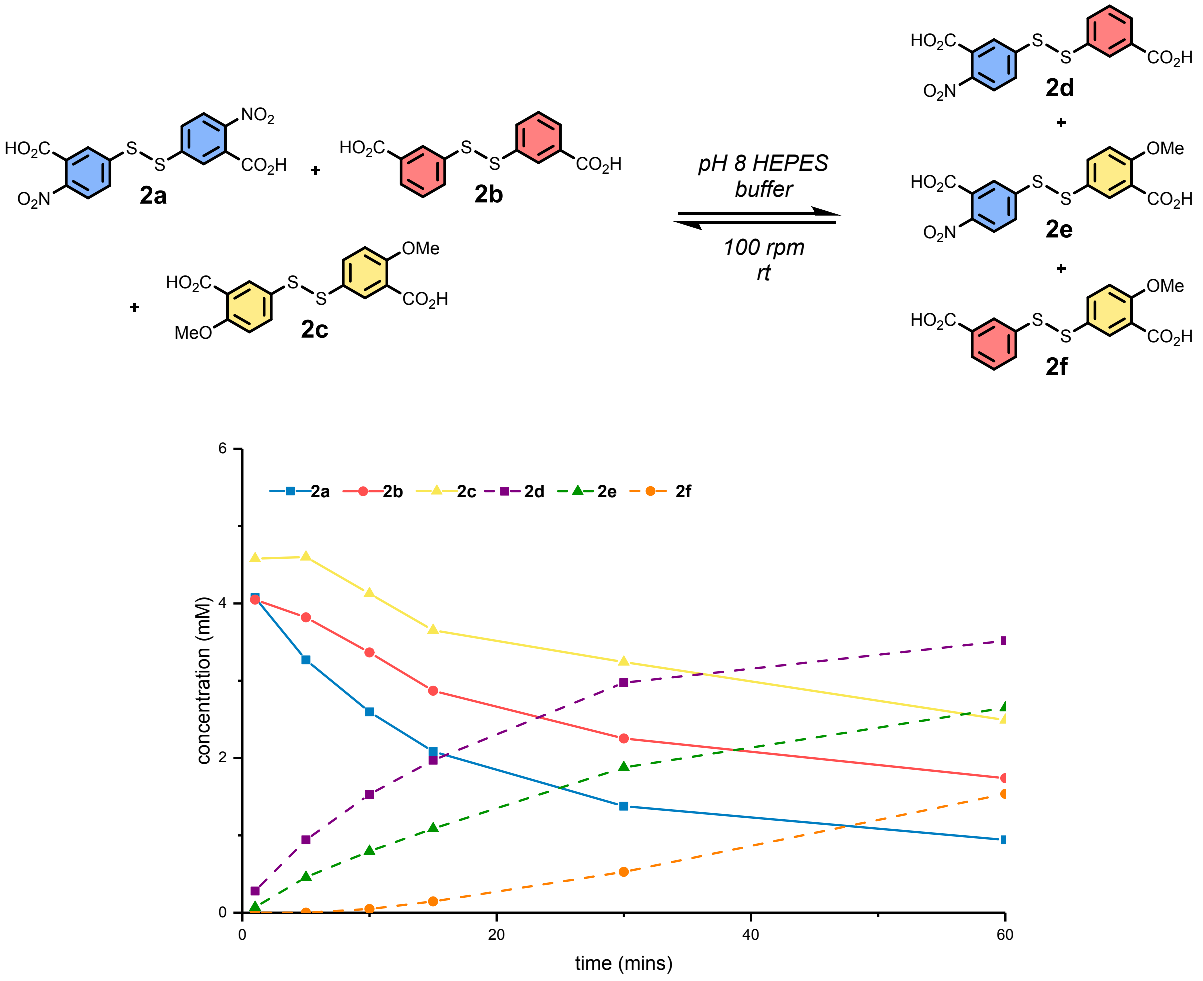

Figure S25 Disulfide metathesis between **2a-c**. HEPES buffer (pH 8.0), r.t., 100 rpm

Seeding experiments

Independent reactions with and without product seed (3.5 mM) were conducted to test for the presence of autocatalysis. Reactions seeded with product display accelerated reaction rates, the removal of a lag period (surfactant **4ac**) and achieve higher product concentrations on the same timescale compared to the unseeded control. Together this shows that these compounds are weakly autocatalytic alone in non-competition experiments. These characteristics are consistent with our previous studies of self-replicators and with the modest hydrophobicity of the thiol tails.[1–3]

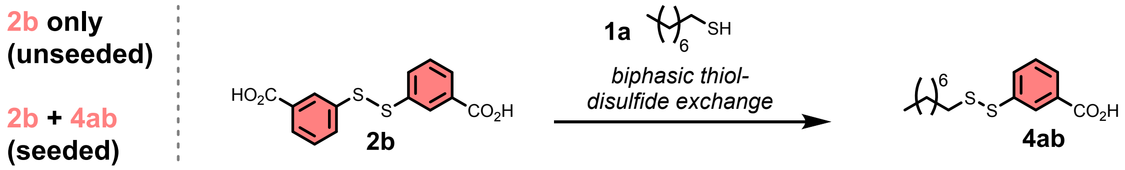

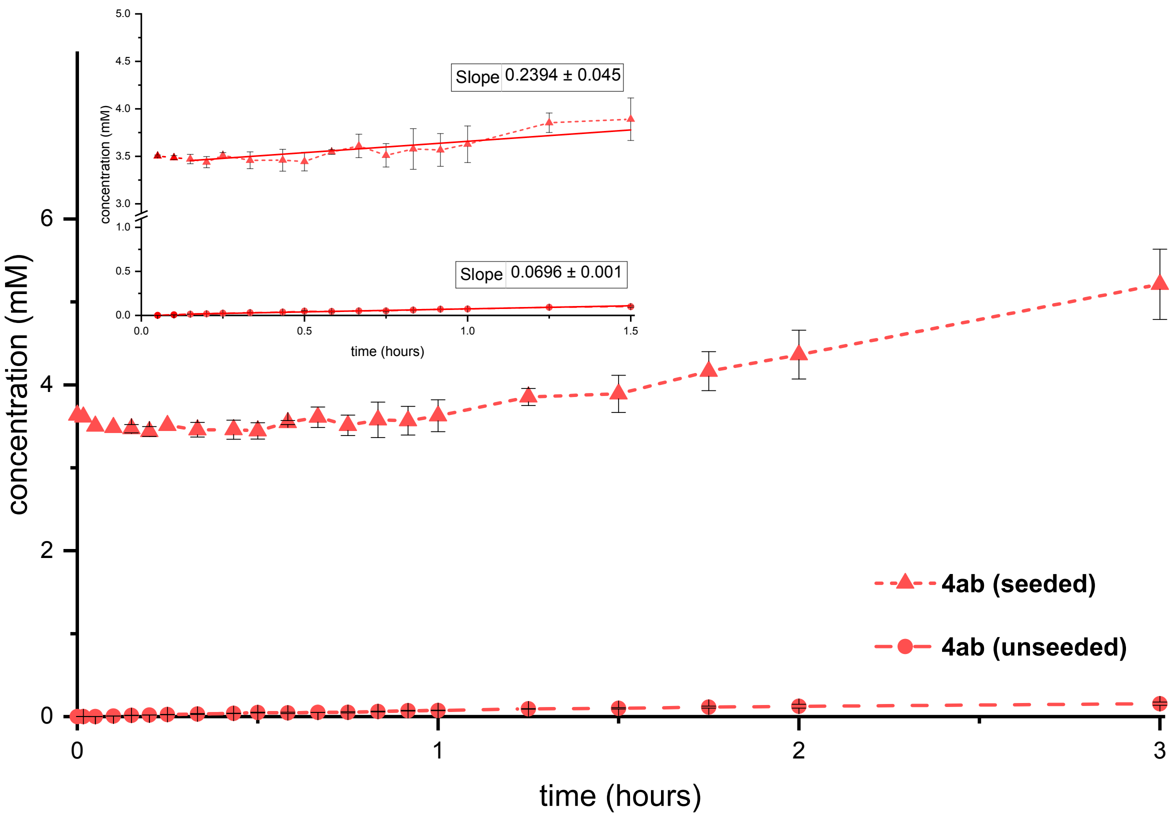

Figure S26 Independent product seeded and unseeded biphasic reactions between ***2b*** *and thiol* ***1a****. Inset plot compares the initial rates. Slope (unseeded) = 0.176 mMh^{-1}, slope (seeded) = 0.984 mMh^{-1}* ***[2b]****$_0$ = 10 mM,* ***[4ab]****$_0$ = 3.5 mM HEPES buffer (pH 8.0), r.t., 100 rpm, 1.0 eq. thiol* ***1*** *n = 3, error bars (where visible) represent the standard deviation. Reactions seeded with product display accelerated reaction rates and achieve higher product concentrations on the same timescale compared to the unseeded control, providing evidence of autocatalytic kinetics*

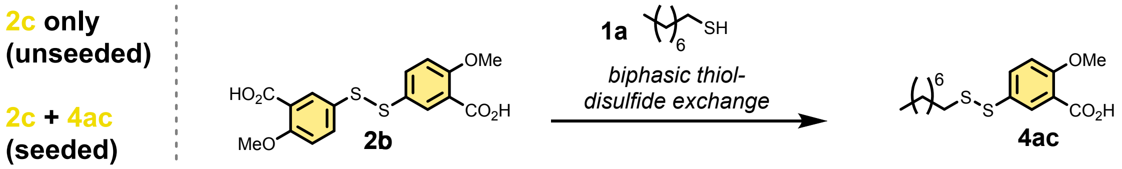

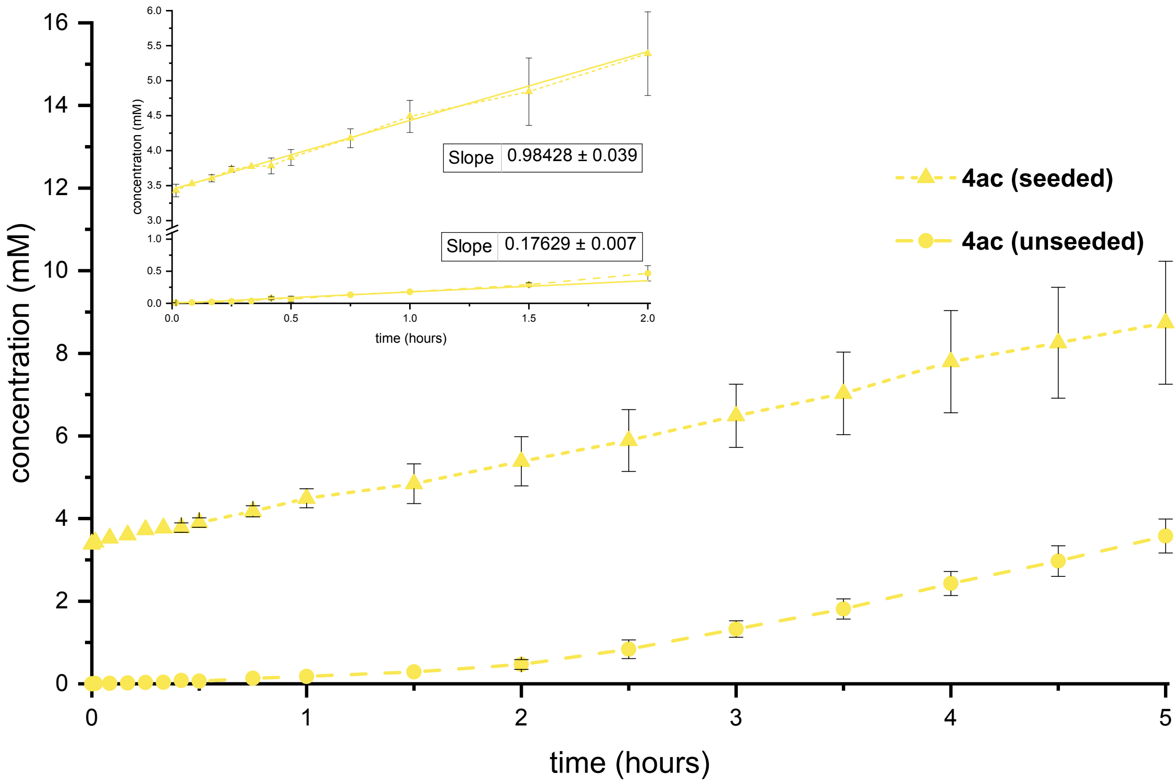

*Figure S27 Independent product seeded and unseeded biphasic reactions between **2c** and thiol **1a**. Inset plot compares the initial rates. Slope (unseeded) = 0.176 mMh^{-1}, slope (seeded) = 0.984 mMh^{-1}**.** [**2c**]$_0$ = 10 mM, [**4ac**]$_0$ = 3.5 mM HEPES buffer (pH 8.0), r.t., 100 rpm, 1.0 eq. thiol **1** n = 3, error bars (where visible) represent the standard deviation. Reactions seeded with product display accelerated reaction rates, the removal of a lag period and achieve higher product concentrations on the same timescale compared to the unseeded control, providing evidence of autocatalytic kinetics*

Synthetic procedures

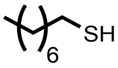

1

octane-1-thiol (**1a**): commercially available and was used without further purification.

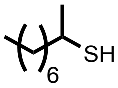

1'

nonane-2-thiol (**1b**): was synthesised according to a literature procedure.[4]
^{1}H NMR (400 MHz, $CDCl_3$) δ 2.93 (hept, J = 7.0 Hz, 1H), 1.60 - 1.45 (m, 3H), 1.32 (d, J = 7.0 Hz, 3H), 1.31 - 1.22 (m, 10H), 0.91- 0.85 (m, 3H); ^{13}C NMR (101 MHz, $CDCl_3$) δ 41.0, 35.6, 31.8, 29.3, 29.2, 27.5, 25.6, 22.6, 14.1.

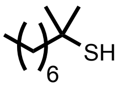

1''

2-methylnonane-2-thiol (**1c**): was synthesised according to a literature procedure.[4]
^{1}H NMR (400 MHz, $CDCl_3$) δ 1.67 (s, 1H), 1.58 – 1.52 (m, 2H), 1.46 - 1.39 (m, 2H), 1.37 (s, 6H), 1.33 - 1.23 (m, 8H), 0.89 (t, J = 7.0 Hz, 3H); ^{13}C NMR (101 MHz, $CDCl_3$) δ 44.6, 44.8, 32.8, 31.8, 30.0, 29.3, 25.4, 24.7, 14.1.

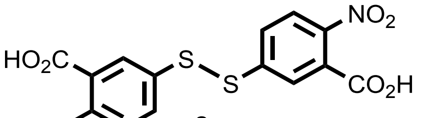

2a

5,5'-dithiobis(2-nitrobenzoic acid) (**2a**): commercially available and was used without further purification.

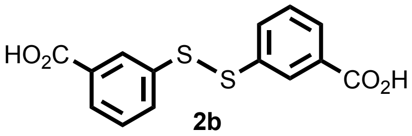

2b

3,3'-dithiobis(benzoic acid) (**2b**): A solution of commercial **3b** (1.85 g, 12.0 mmol, 1.0 equiv.) in DMSO (40 mL) was stirred at room temperature for 18 hours before acidification with 1 M aq. HCl (200.0 mL), forming a white precipitate. This was filtered and washed with 1 M aq. HCl (2 x 20 mL). Th e solid was dissolved in MeOH/$CHCl_3$ (1:1) at 40 °C, passed through a celite plug and the filtrate concentrated to dryness to give 3,3'-dithiobis(benzoic acid) (**2b**) as an off-white powder (1.636 g, 10.7 mmol, 89%).

^{1}H NMR (400 MHz, DMSO-d_6): δ 8.08 (t, J = 1.8 Hz, 2H), 7.85 (dt, J = 7.7, 1.3 Hz, 2H), 7.74 (ddd, J = 7.9, 2.1, 1.1 Hz, 2H), 7.51 (t, J = 7.8 Hz, 2H); ^{13}C NMR (101 MHz, DMSO-d_6): 166.7, 136.1, 133.0, 130.9, 129.7, 128.5, 127.5; HRMS (ESI+, m/z): [M-H]- calc. for $C_{14}H_9O_4{}^{32}S_2$, 304.9948; found, 304.9949; m.p. 249.9 - 250.4 °C

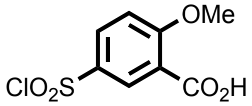

6

5-(chlorosulfonyl)-2-methoxybenzoic acid (**6**): 2-methoxybenzoic acid (2.50 g, 16.4 mmol, 1.0 eq.) was added to HSO_3Cl (4.92 mL, 73.8 mmol, 4.5 eq.) in portions over 10 minutes with ice cooling. After reacting at 0 °C for 30 minutes, $SOCl_2$ (1.19 mL, 16.4 mmol, 1.0 eq.) was added dropwise and the mixture stirred at room temperature for 3 hours. The solution was poured onto ice and the precipitate collected by vacuum filtration with cold 1 M aq. HCl washings. Drying *in vacuo* gave **6** as a white powder (3.93 g, 96% yield).

^{1}H NMR (400 MHz, $CDCl_3$): δ 8.77 (m, 1H), 8.22 (dd, J = 9.0, 2.6 Hz, 1H), 7.25 (d, J = 9.0 Hz, 1H), 4.17 (s, 3H); ^{13}C NMR (101 MHz, $CDCl_3$): δ 173.2, 163.2, 137.4, 133.7, 133.4, 119.4, 113.1, 57.7; HRMS (ESI+, m/z): [M-H]- calc. for $C_8H_5O_5{}^{35}Cl^{32}S$, 248.9630; found, 248.9629; m.p. 145.6 - 147.7 °C Lit[5]: m.p. 138 - 140 °C, ^{1}H NMR ($CDCl_3$): δ 8.78 (s, 1H), 8.21 (s, 1H), 7.23 (d, 1H), 4.18 (s, 3H); 94% yield.

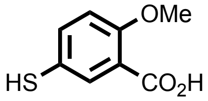

3c

5-mercapto-2-methoxybenzoic acid (**3c**): To a suspension of **6** (10.05 g, 40.1 mmol, 1.0 eq.) in AcOH (120.0 mL) was added a suspension of $SnCl_2{\bullet}2H_2O$ (54.30 g, 240.6 mol, 6.0 eq.) in conc. HCl (37%, 60.0 mL) at room temperature. The mixture was then heated to 80 °C for 16 hours. The cooled solution was then poured onto ice and extracted with $CHCl_3$/MeOH (9:1, 5 x 50 mL) before washing of the combined organics with acidified brine (100 mL) and drying with $MgSO_4$. Concentration of the organic layer to dryness in vacuo gave **3c** as a pale yellow powder (6.65 g, 91% yield).

^{1}H NMR (400 MHz, acetone-d_6): δ 11.00 (s, 1H), 7.82 (d, J = 2.6 Hz, 1H), 7.53 (dd, J = 2.5, 8.7 Hz, 1H), 7.15 (d, J = 8.7 Hz, 1H), 3.97 (s, 3H); ^{13}C NMR (101 MHz, acetone-d_6): δ 165.9, 158.0, 136.1, 133.9, 123.2, 121.3, 114.4, 56.9; HRMS (ESI-, m/z): [M-H]- calc. for $C_8H_7O_3S$, 183.0121; found, 183.0120; m.p. 103.1 – 104.3 °C

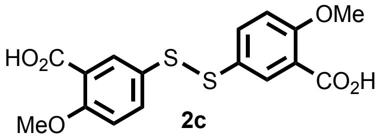

2c

5,5'-dithiobis(2-methoxybenzoic acid) (**2c**): was synthesised in an analogous fashion to **2b** from **3c** (6.615 g, 35.9 mmol) to give **2c** as a white powder (6.469 g, 98% yield).

^{1}H NMR (400 MHz, DMSO-d_6): δ 12.84 (br s, 1H), 7.72 (d, J = 2.5 Hz, 1H), 7.60 (dd, J = 8.7, 2.5 Hz, 1H), 7.16 (d, J = 8.8 Hz, 1H), 3.83 (s, 3H); ^{13}C NMR (101 MHz, DMSO-d_6): δ 166.3, 158.4, 134.7, 132.3, 126.3, 122.3, 113.8, 56.1; HRMS (ESI-, m/z): [M-H]- calc. for $C_{16}H_{13}O_6{}^{32}S_2$, 365.0159; found, 365.0158; m.p. 215.8 - 218.0 °C

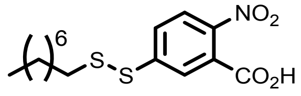

4aa

2-nitro-5-(octyldisulfaneyl)benzoic acid (**4aa**): To a solution of triethylamine (702 µL, 5.04 mmol, 2.0 equiv.) and **2a** (1.00 g, 2.52 mmol, 1.0 equiv.) in DCM (10 mL) was dropwise added a solution of triethylamine (351 µL, 2.52 mmol, 1.0 equiv.)

and thiol **1a** (437 µL, 2.52 mmol, 1.0 equiv.) in DCM (10 mL). After 1 hour at room temperature, the reaction was diluted with 1 M aq. HCl (10 mL) extracted with DCM (3 x 20 mL). The combined organics were dried (NaSO4) and the solvent removed *in vacuo*. The crude material was purified by column chromatography (methanol/chloroform (1:99)) to give **4aa** as a colourless oil (766.2 mg, 2.23 mmol, 88% yield).

^{1}H NMR (400 MHz, DMSO-d$_6$) δ 7.92 (d, J = 8.5 Hz, 1H), 7.83 (d, J = 2.0 Hz, 1H), 7.77 (dd, J = 8.5, 2.0 Hz, 1H), 2.82 (t, J = 7.0 Hz, 2H), 1.59 (p, J = 7.0 Hz, 2H), 1.37 – 1.27 (m, 2H), 1.27 – 1.12 (m, 8H), 0.83 (t, J = 7.0 Hz, 3H); ^{13}C NMR (101 MHz, DMSO-d$_6$) δ 166.0, 146.0, 143.6, 127.2, 125.9, 124.4, 120.8, 38.1, 31.1, 28.5, 28.2, 27.6, 22.0, 13.9; HRMS (ES-, m/z) calc. for $C_{15}H_{20}O_4N^{32}S_2$ [M-H]- 342.0828, found 342.0834. Data consistent with literature values.[3]

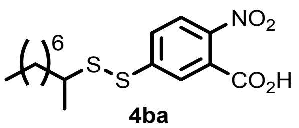

4ba

(±)-2-nitro-5-(nonan-2-yldisulfaneyl)benzoic acid (**4ba**): was synthesised in an analogous fashion to **4aa** from **2a** (494.6 mg, 1.25 mmol) and thiol **1b** (200.0 mg, 1.25 mmol) to give a colourless oil (408.5 mg, 1.114 mmol, 91% yield).

^{1}H NMR (400 MHz, DMSO-*d$_6$*) δ 8.02 (d, *J* = 8.5 Hz, 1H), 7.93 (d, *J* = 2.0 Hz, 1H), 7.90 (dd, *J* = 8.5, 2.0 Hz, 1H), 3.05 (hept, *J* = 6.5 Hz, 1H), 1.61 – 1.40 (m, 2H), 1.33 (p, *J* = 7.5 Hz, 2H), 1.24 (d, *J* = 6.5 Hz, 3H), 1.22 – 1.08 (m, 8H), 0.83 (t, *J* = 7.0 Hz, 3H); ^{13}C NMR (101 MHz, DMSO-*d$_6$*) δ 165.6, 145.7, 145.0, 128.8, 128.3, 125.9, 124.8, 46.9, 35.2, 31.2, 28.7, 28.5, 26.3, 22.0, 20.3, 13.9; HRMS (ES-, m/z) calc. for $C_{16}H_{22}O_4N^{32}S_2$ [M-H]- 356.0996, found 356.0993.

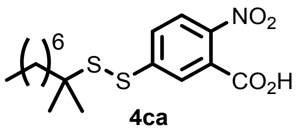

4ca

2-nitro-5-((2-methylnonan-2-yl)disulfaneyl)benzoic acid (**4ca**): was synthesised in an analogous fashion to **4aa** from **2a** (454.6 mg, 1.15 mmol) and thiol **1c** (200.0 mg, 1.15 mmol) to give a colourless oil (291.1 mg, 0.784 mmol, 68% yield).

^{1}H NMR (400 MHz, DMSO-*d$_6$*) δ 8.00 (d, *J* = 8.5 Hz, 1H), 7.94 (d, *J* = 2.0 Hz, 1H), 7.92 (dd, *J* = 8.5, 2.0 Hz, 1H), 1.52 – 1.45 (m, 2H), 1.29 – 1.12 (m, 14H), 1.10 – 1.01 (m, 2H), 0.83 (t, *J* = 7.0 Hz, 3H); ^{13}C NMR (101 MHz, DMSO-*d$_6$*) δ 165.6, 145.7, 145.2, 128.7, 126.3, 124.6, 53.7, 40.5, 31.2, 29.3, 28.5, 27.2, 24.2, 22.0, 13.9; HRMS (ES-, m/z) calc. for $C_{17}H_{22}O_4N^{32}S_2$ [M-H]- 370.1152, found 370.1149.

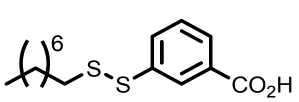

4ab

3-(octyldisulfaneyl)benzoic acid (**4ab**): was synthesised in an analogous fashion to **4aa** from **2b** (100.0 mg, 0.326 mmol) and thiol **1a** (56.5 µL, 0.326 mmol) to give a colourless oil (68.0 mg, 0.228 mmol, 70% yield).

^{1}H NMR (400 MHz, DMSO-*d$_6$*) δ 8.10 (t, *J* = 2.0 Hz, 1H), 7.82 (dt, *J* = 7.5, 1.5 Hz, 1H), 7.76 (ddd, *J* = 8.0, 2.0, 1.0 Hz, 1H), 7.51 (t, *J* = 8.0 Hz, 1H), 2.80 (t, *J* = 7.0 Hz, 2H), 1.58 (p, *J* = 7.0 Hz, 2H) 1.36 – 1.12 (m, 10H), 0.83 (t, *J* = 7.0 Hz, 3H), 0.83 (t, *J* = 7.0 Hz, 3H); ^{13}C NMR (101 MHz, DMSO-*d$_6$*) δ 166.6, 137.6, 131.9, 131.0, 129.5, 127.7, 127.1, 38.3, 31.1, 28.4, 28.4, 28.1, 27.5, 22.0, 13.9; HRMS (ES-, m/z) calc. for $C_{15}H_{21}O_2{}^{32}S_2$ [M-H]- 297.0988, found 297.0985.

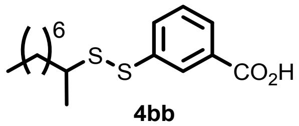

4bb

(±)-3-(nonan-2-yldisulfaneyl)benzoic acid (**4bb**): was synthesised in an analogous fashion to **4aa** from **2b** (100.0 mg, 0.326 mmol) and thiol **1b** (52.3 mg, 0.326 mmol) to give a colourless oil (56.9 mg, 0.183 mmol, 56% yield).

^{1}H NMR (400 MHz, DMSO-d_6) δ 8.12 (t, *J* = 2.0 Hz, 1H), 7.80 (dt, *J* = 7.5, 1.5 Hz, 1H), 7.76 (ddd, *J* = 8.0, 2.0, 1.0 Hz, 1H), 7.49 (t, *J* = 8.0 Hz, 1H), 2.97 (hept, *J* = 6.5 Hz, 1H), 1.59 – 1.44 (m, 1H), 1.43 – 1.38 (m, 1H), 1.33 – 1.04 (m, 13H), 0.83 (t, *J* = 7.0 Hz, 3H); ^{13}C NMR (101 MHz, DMSO-d_6) δ 166.6, 138.1, 131.0, 129.4, 127.6, 127.2, 46.6, 35.1, 31.1, 28.7, 28.4, 26.2, 22.0, 20.4, 13.9; HRMS (ES-, m/z) calc. for $C_{16}H_{23}O_2{}^{32}S_2$ [M-H]- 311.1145, found 311.1142.

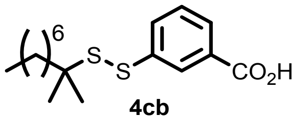

4cb

3-((2-methylnonan-2-yl)disulfaneyl)benzoic acid (**4cb**): was synthesised in an analogous fashion to **4aa** from **2b** (100.0 mg, 0.326 mmol) and thiol **1c** (56.8 mg, 0.326 mmol) to give a colourless oil (24.8 mg, 0.076 mmol, 23% yield).

^{1}H NMR (400 MHz, DMSO-d_6) δ 8.13 (t, *J* = 2.0 Hz, 1H), 7.82 – 7.76 (m, 2H), 7.48 (t, *J* = 8.0 Hz, 1H), 1.47 – 1.39 (m, 2H), 1.29 – 1.04 (m, 14H), 1.00 – 0.89 (m, 2H), 0.82 (t, *J* = 7.0 Hz, 3H); ^{13}C NMR (101 MHz, DMSO-d_6) δ 166.6, 138.4, 131.7, 129.2, 127.6, 127.6, 52.9, 40.5, 31.2, 29.2, 28.5, 27.2, 24.2, 22.0, 13.9; HRMS (ES-, m/z) calc. for $C_{17}H_{25}O_2{}^{32}S_2$ [M-H]- 325.1290, found 325.1299.

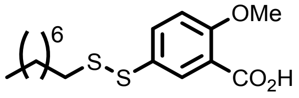

4ac

2-methoxy-5-(octyldisulfaneyl)benzoic acid (**4ac**): To a solution of **2c** (366.4 mg, 1.00 mmol) in HEPES buffer (40.0 mL, pH 8.00, 0.25 M) was added thiol **1a** (1.38 mL, 8.00 mmol). After 22 hours stirring (100 rpm) at room temperature, the reaction was diluted with 1 M aq. HCl (20 mL) extracted with methanol/chloroform (1:9, 5 x 50 mL). The combined organics were washed with acidified brine (100 mL), dried ($MgSO_4$) and the solvent removed *in vacuo*. The crude material was purified by column and preparative thin layer chromatography (methanol/chloroform (3:97)) to give **4ac** as a clear yellow oil (188.1 mg, 0.69 mmol, 69% yield).

^{1}H NMR (400 MHz, DMSO-d_6): δ 7.77 (d, J = 2.6 Hz, 1H), 7.65 (dd, J = 8.6, 2.4 Hz, 1H), 7.15 (d, J = 8.7 Hz, 1H), 3.82 (s, 1H), 2.76 (t, J = 7.1 Hz, 2H), 1.59 (p, J = 7.2 Hz, 2H), 1.36-1.12 (m, 10H), 0.84 (t, J = 7.0 Hz, 3H); ^{13}C NMR (101 MHz, DMSO-d_6): δ 166.6, 157.9, 134.1, 131.6, 127.2, 122.4, 113.6, 56.0, 38.1, 31.2, 28.5, 28.5, 28.1, 27.6, 22.1, 14.0; HRMS (ESI-, m/z): [M-H]- calc. for $C_{16}H_{23}O_3{}^{32}S_2$, 327.1094; found, 327.1097

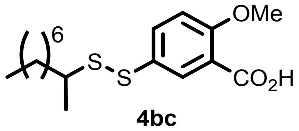

4bc

(±)-2-methoxy-5-(nonan-2-yldisulfaneyl)benzoic acid (**4bc**): was synthesised in an analogous fashion to **4ac** from **2c** (366.4 mg, 1.00 mmol) and thiol **1b** (380 µL, ca. 2.00 mmol), with a reaction time of 52 hours and stir rate of 500 rpm, to give a clear yellow oil (323.6 mg, 0.94 mmol, 94% yield).

^{1}H NMR (400 MHz, DMSO-d_6): δ 7.80 (d, J = 2.6 Hz, 1H), 7.67 (dd, J = 8.7, 2.6 Hz, 1H), 7.14 (d, J = 8.8 Hz, 1H), 3.81 (s, 3H), 2.92 (sextet, J = 6.7 Hz, 1H), 1.61-1.35 (m, 2H), 1.32-1.03 (m, 14H), 0.83 (t, J = 7.0 Hz, 3H),; ^{13}C NMR (101 MHz, DMSO-d_6): δ 166.5, 157.8, 133.8, 131.4, 127.7, 122.0, 113.5, 56.0, 46.4, 35.1, 31.2, 28.7, 28.5, 26.3, 22.0, 20.5, 13.9;

HRMS (ESI+, m/z): [M+H]+ calc. for $C_{17}H_{27}O_3{}^{32}S_2$, 343.1396; found, 343.1397

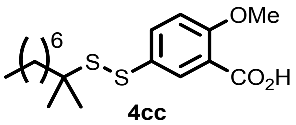

4cc

2-methoxy-5-((2-methylnonan-2-yl)disulfaneyl)benzoic acid (**4cc**): was synthesised in an analogous fashion to **4ac** from **2c** (366.4 mg, 1.00 mmol) and thiol **1c** (380 μL, ca. 2.00 mmol), with a reaction time of 71 hours and stir rate of 500 rpm, to give a clear yellow oil (251.1 mg, 0.70 mmol, 70% yield).

^{1}H NMR (400 MHz, DMSO-d_6): δ 7.81 (d, J = 2.5 Hz, 1H), 7.68 (dd, J = 8.8,2.6 Hz, 1H), 7.13 (d, J = 8.8 Hz, 1H), 3.81 (s, 3H), 1.47-1.38 (m, 2H), 1.26-0.91 (m, 16H), 0.83 (t, J = 7.1, 3H); ^{13}C NMR (101 MHz, DMSO-d_6): δ 166.4, 157.8, 133.8, 131.4, 128.0, 121.8, 113.4, 56.0, 52.6, 40.5, 31.2, 29.3, 28.6, 27.4, 24.3, 22.0, 13.9; HRMS (ESI+, m/z): [M+H]+ calc. for $C_{18}H_{29}O_3S_2$, 357.1553; found, 357.1554

NMR spectra

nonane-2-thiol (1b)

1H NMR
CDCl3
1b

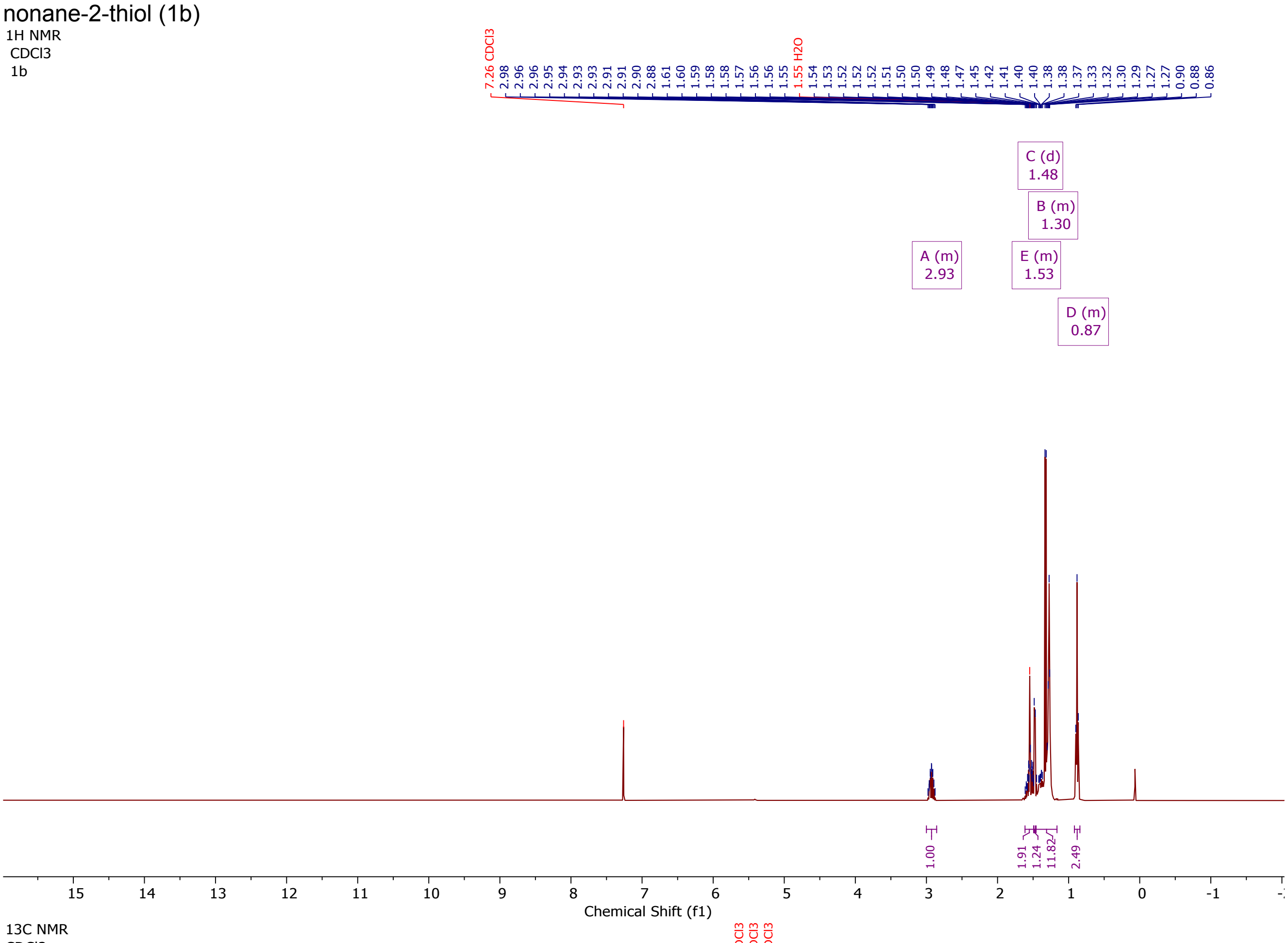

13C NMR
CDCl3
1b

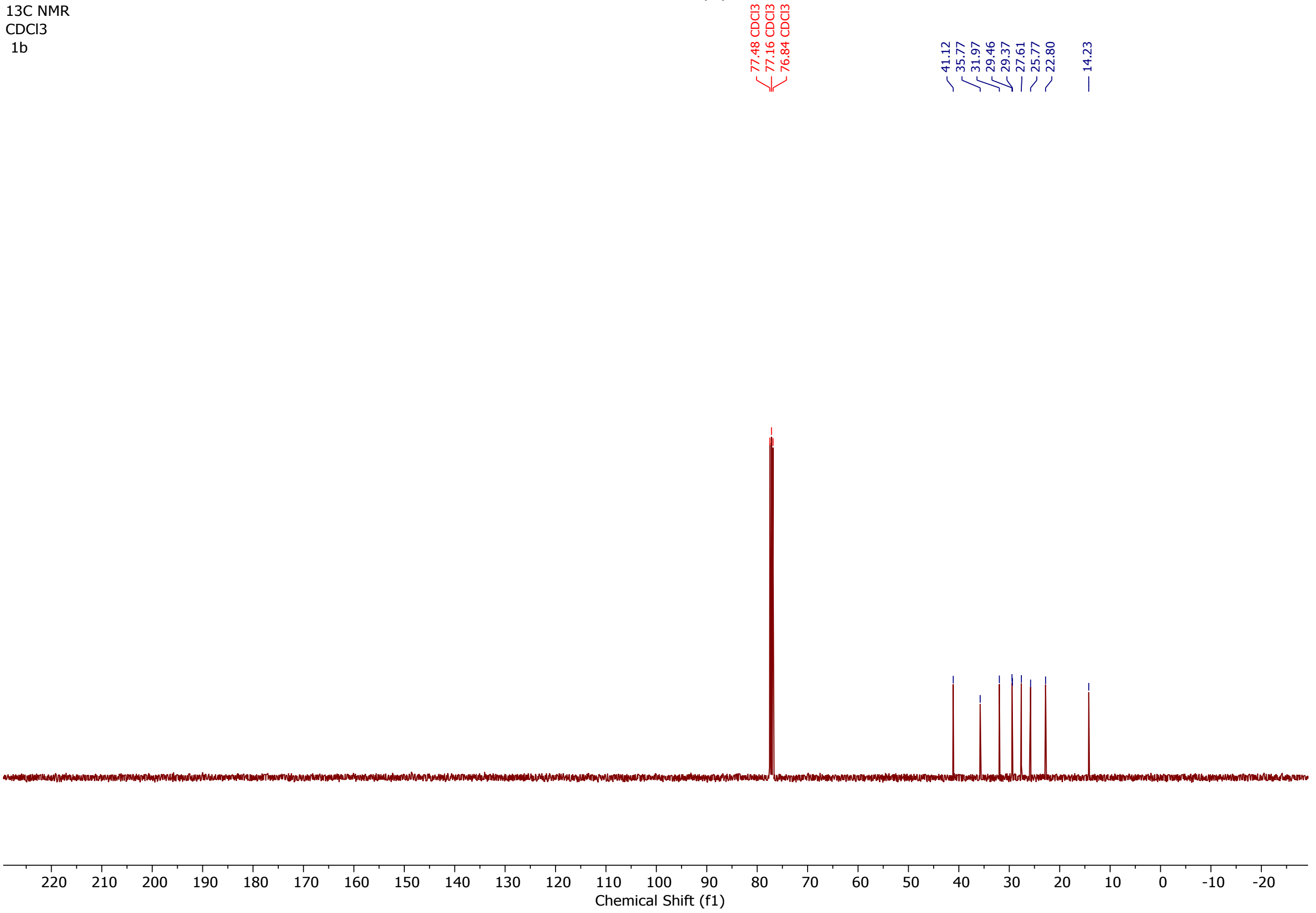

2-methylnonane-2-thiol (1c)

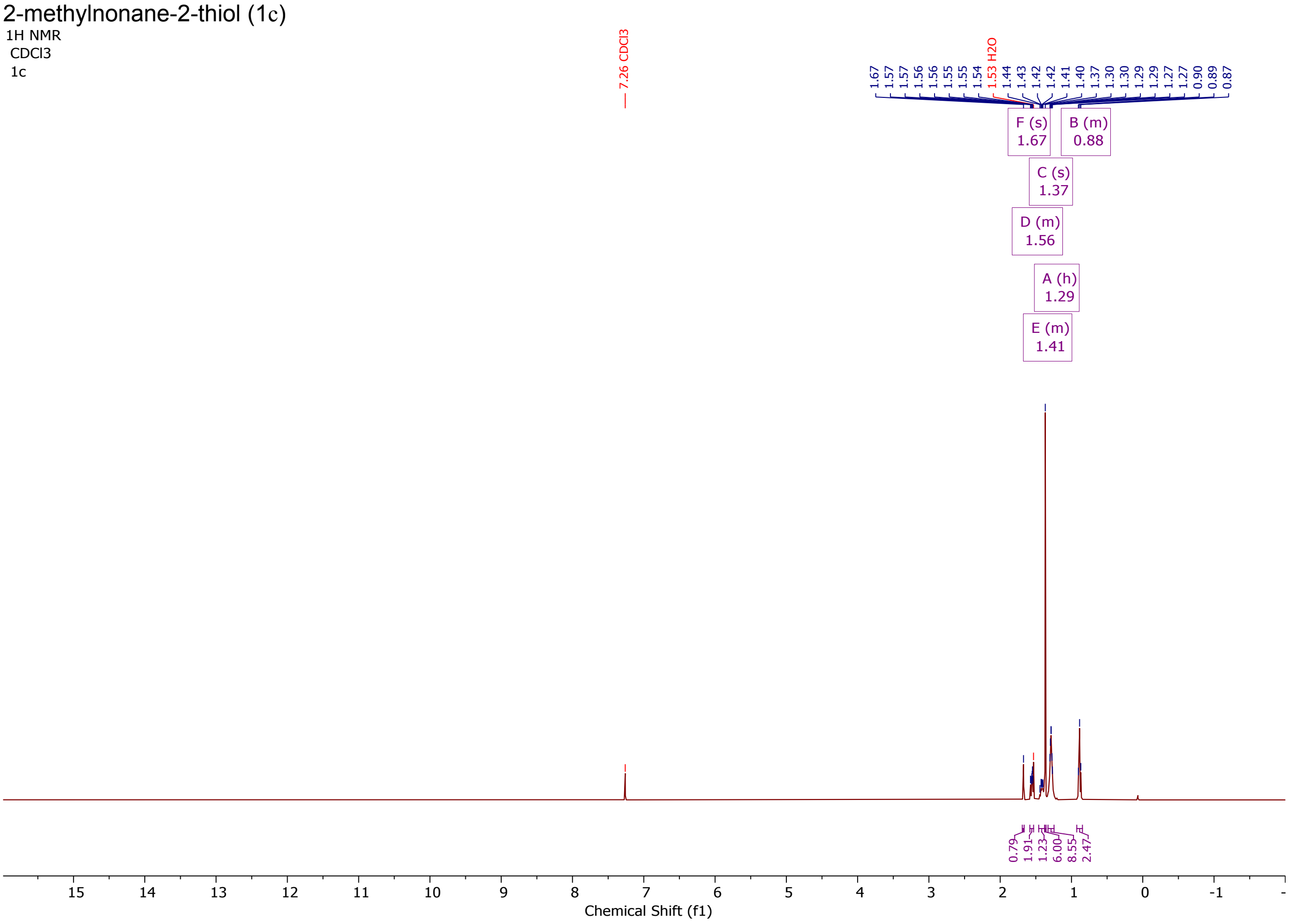

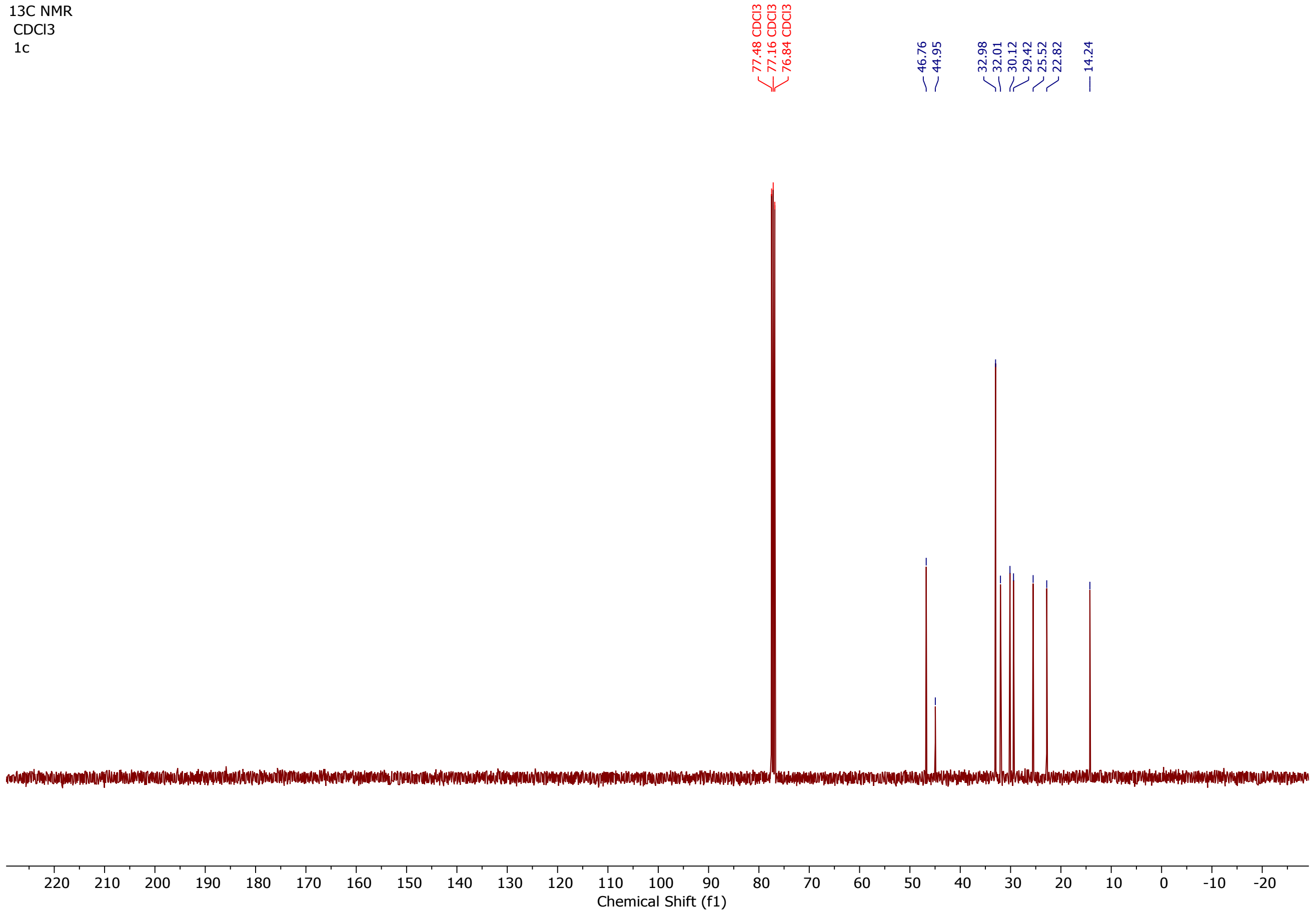

3,3'-dithiobis(benzoic acid) (2b)

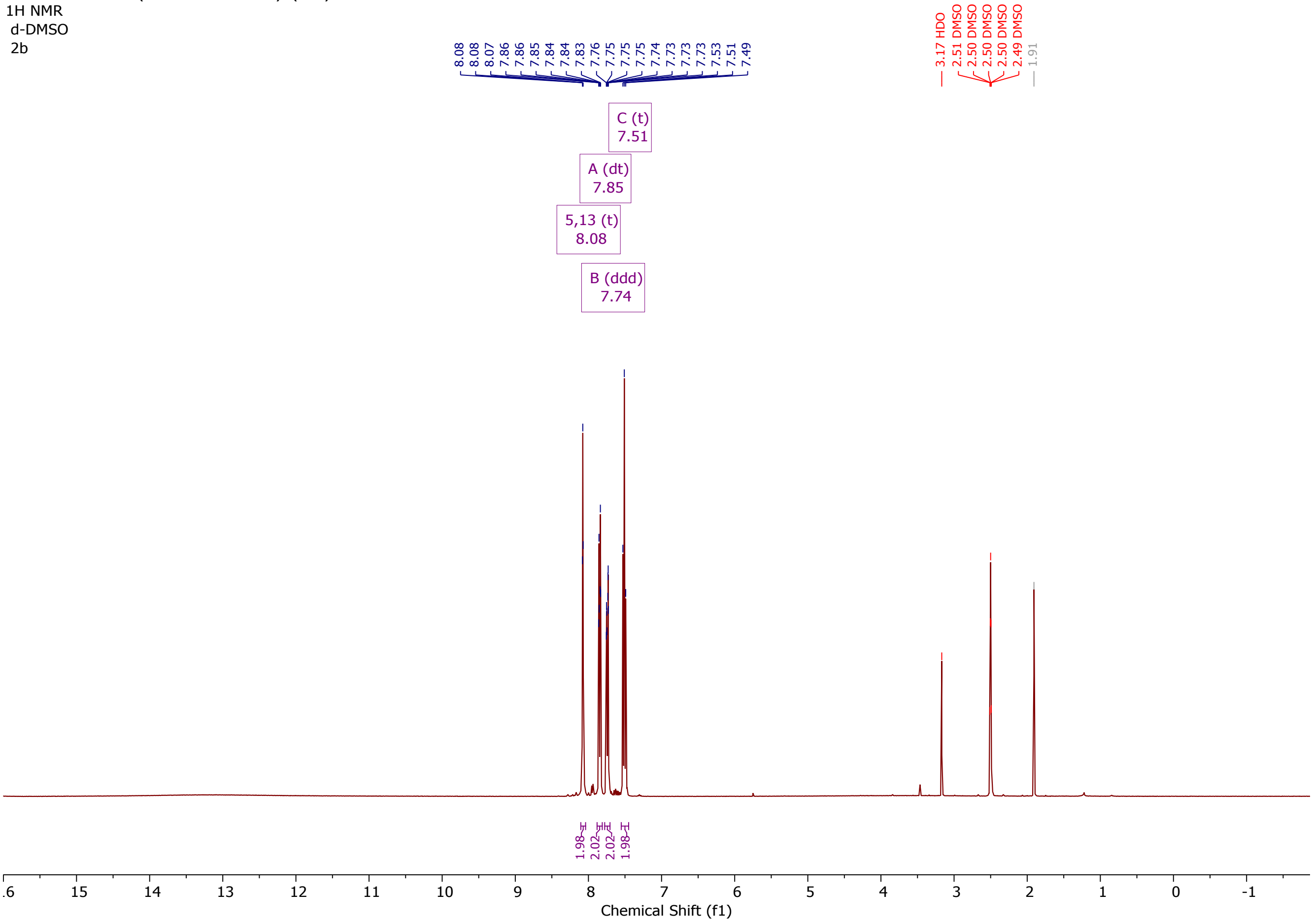

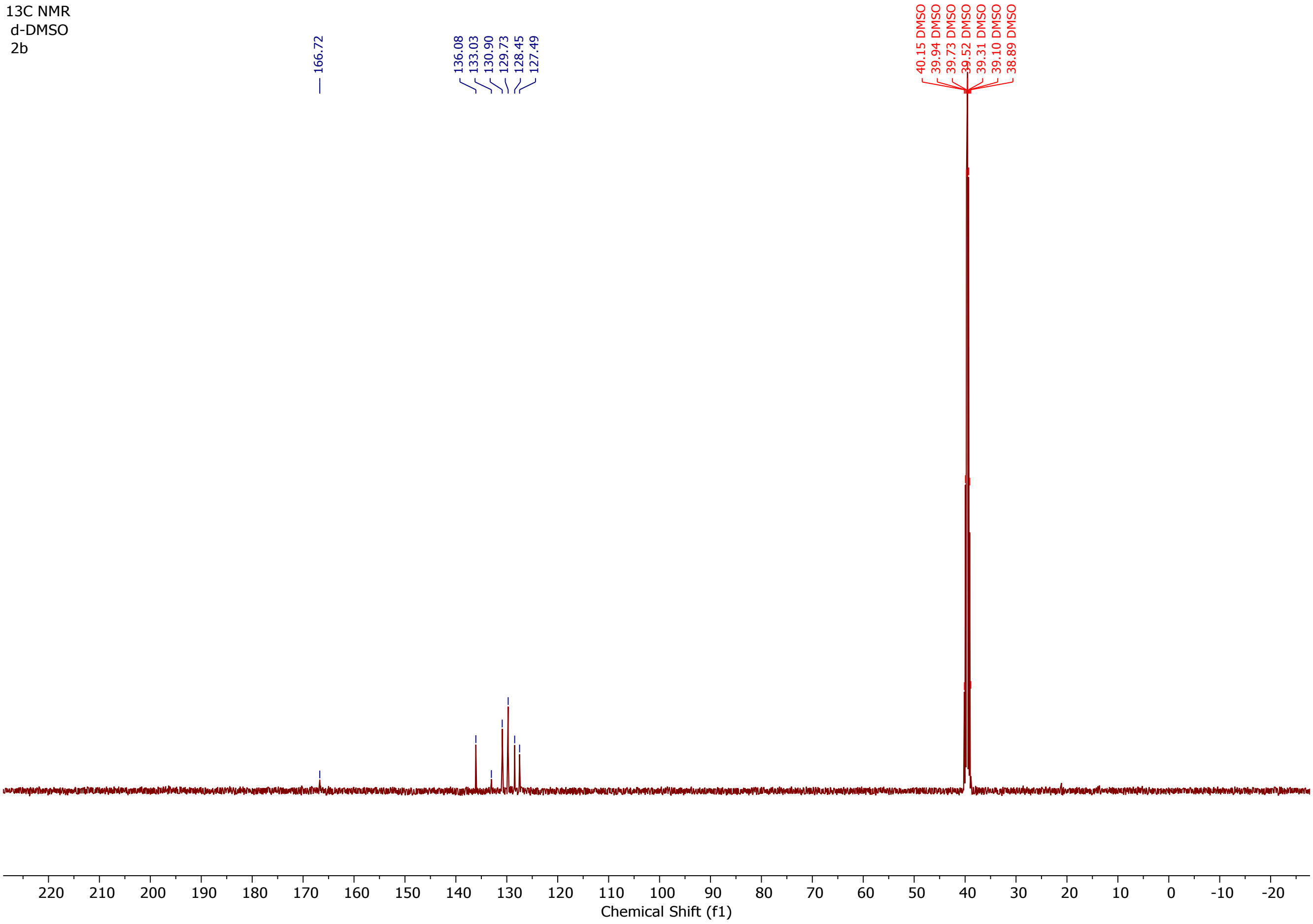

5-(chlorosulfonyl)-2-methoxybenzoic acid (6)

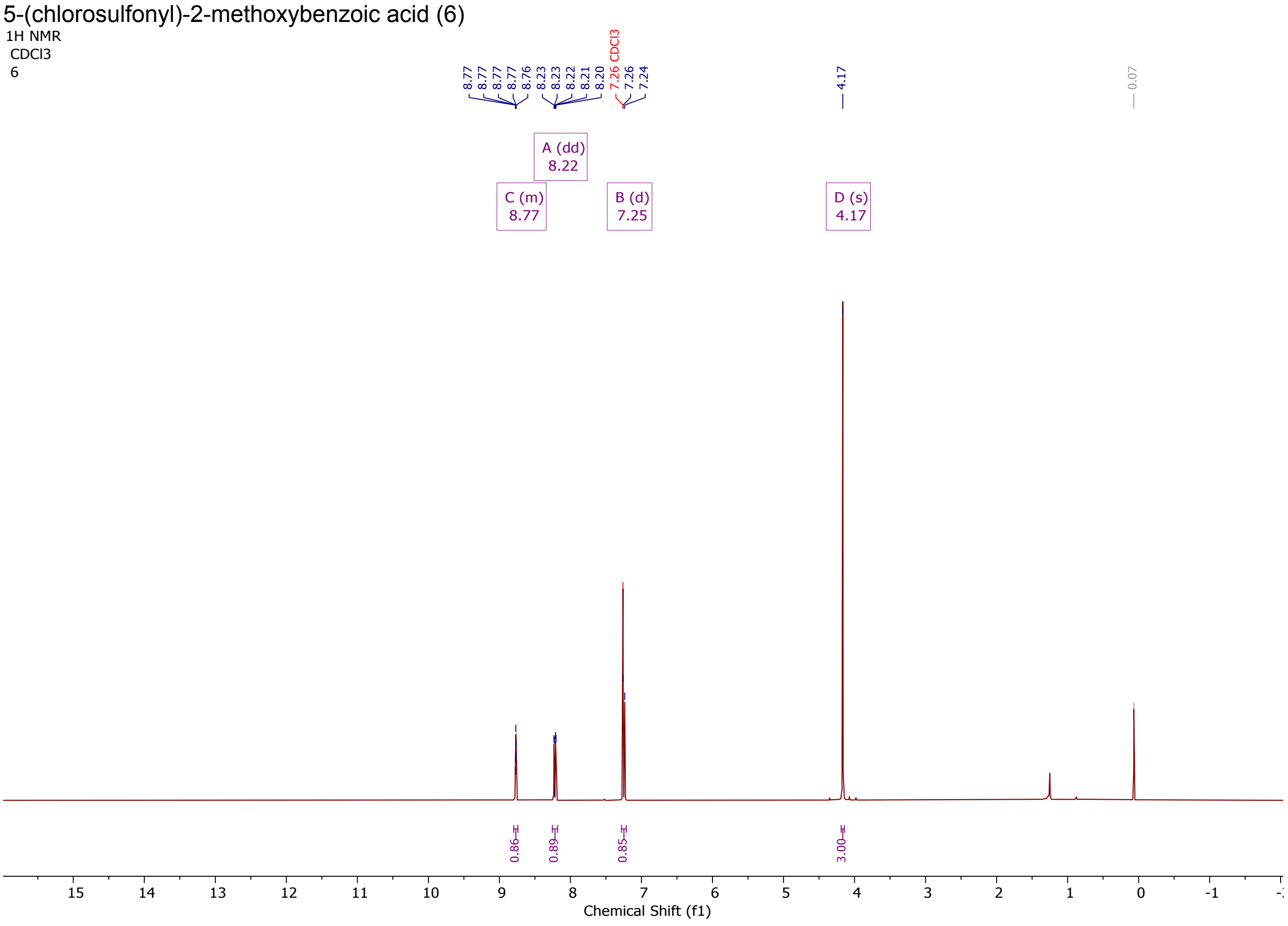

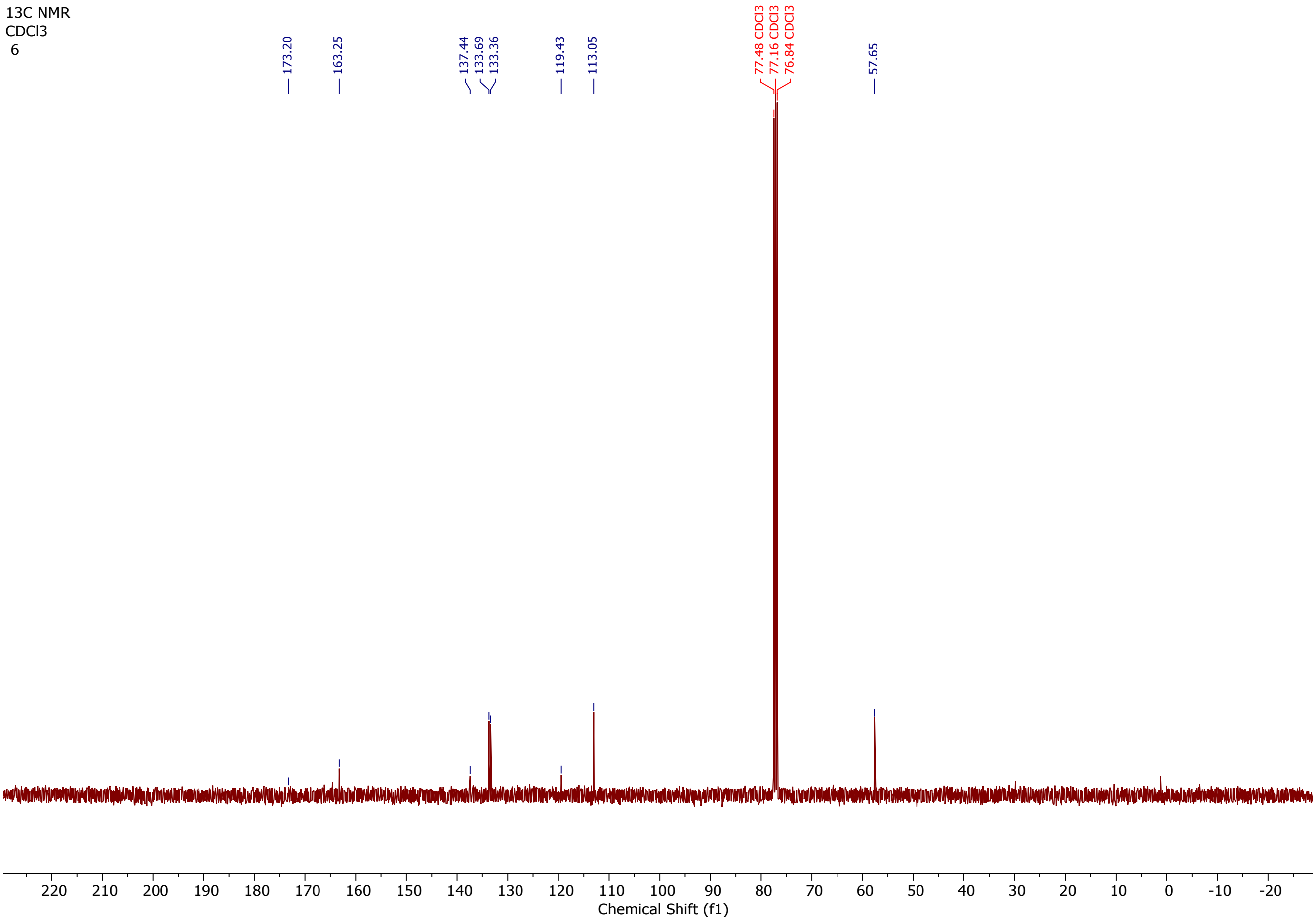

5-mercapto-2-methoxybenzoic acid (3c)

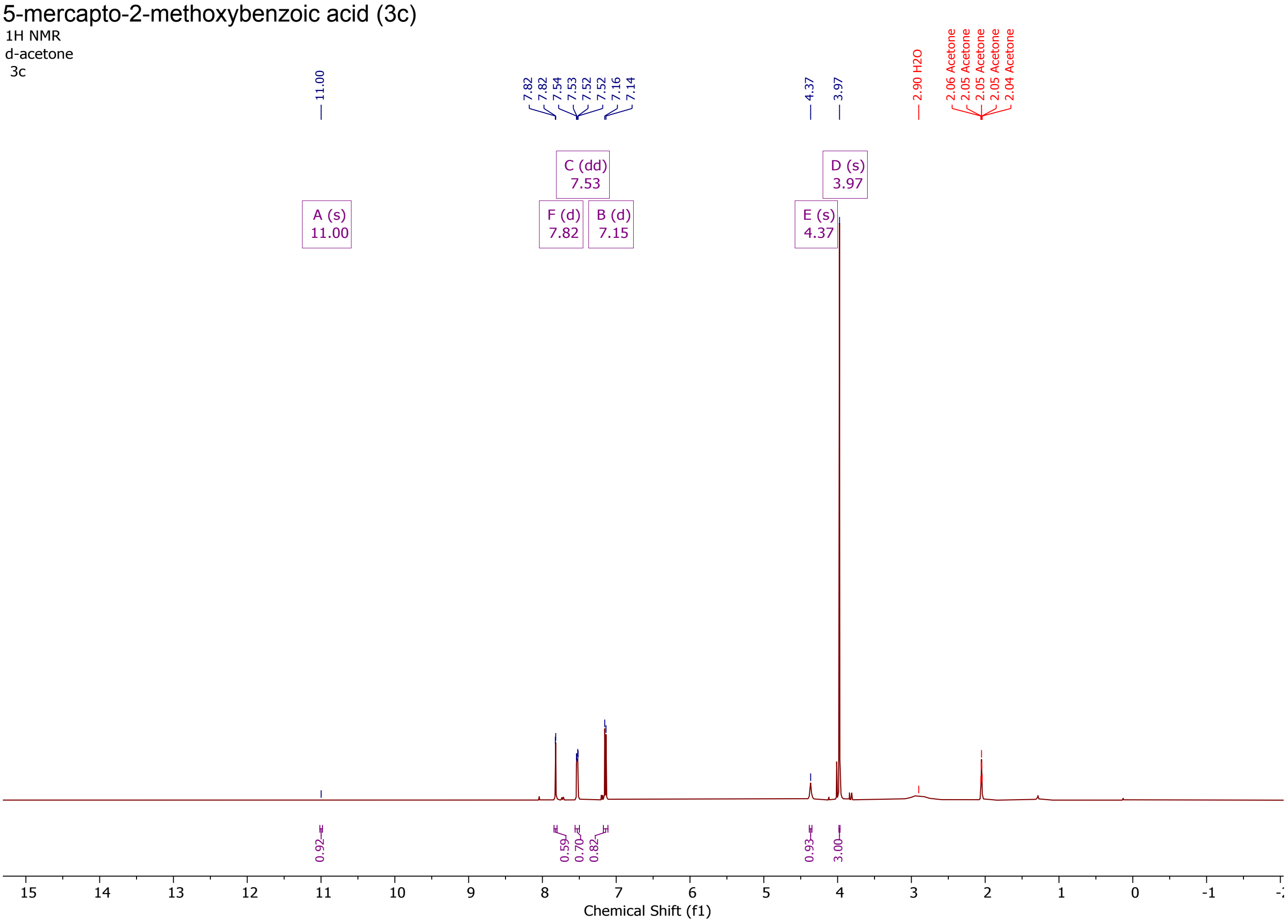

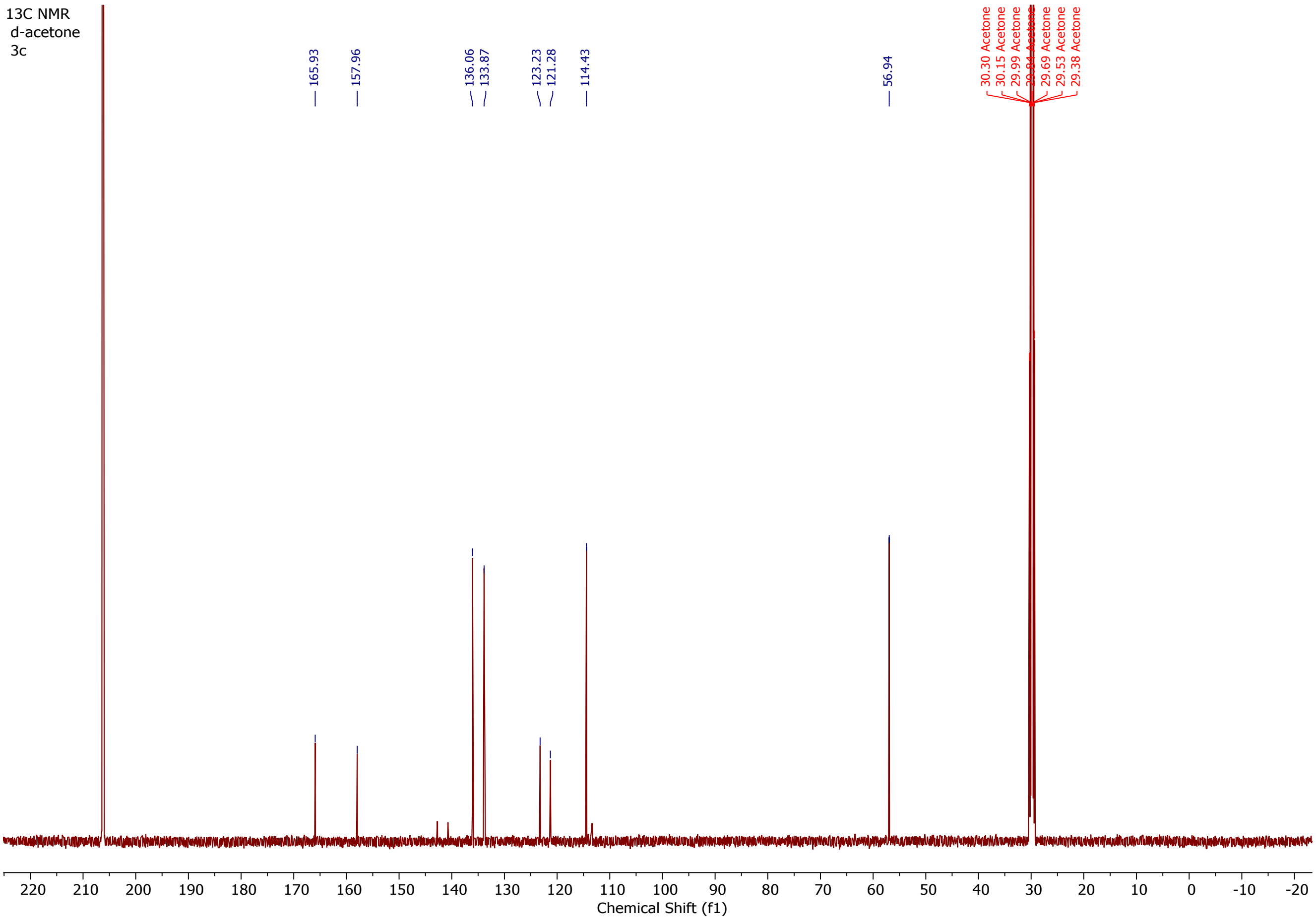

5,5’-dithiobis(2-methoxybenzoic acid) (2c)

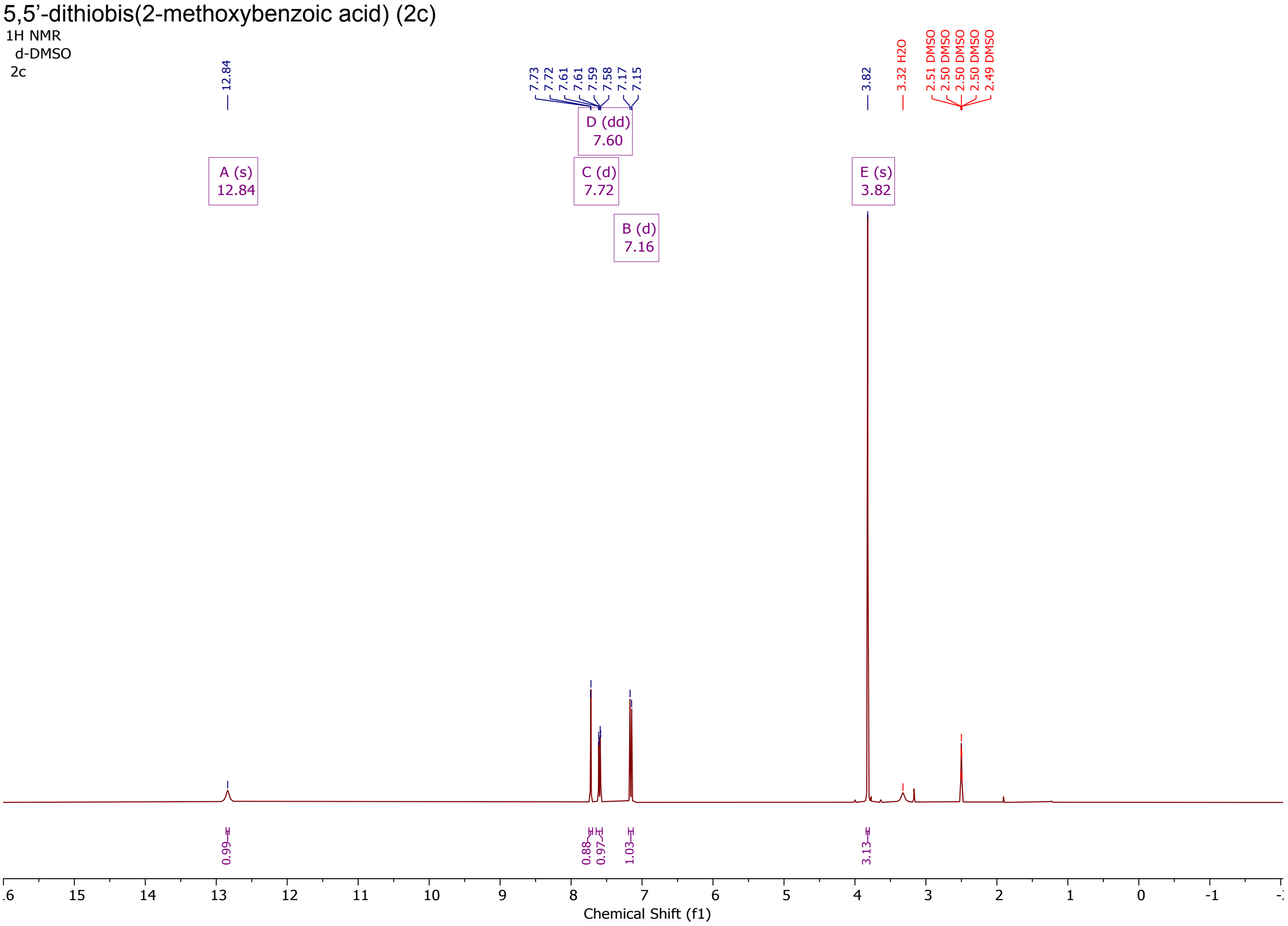

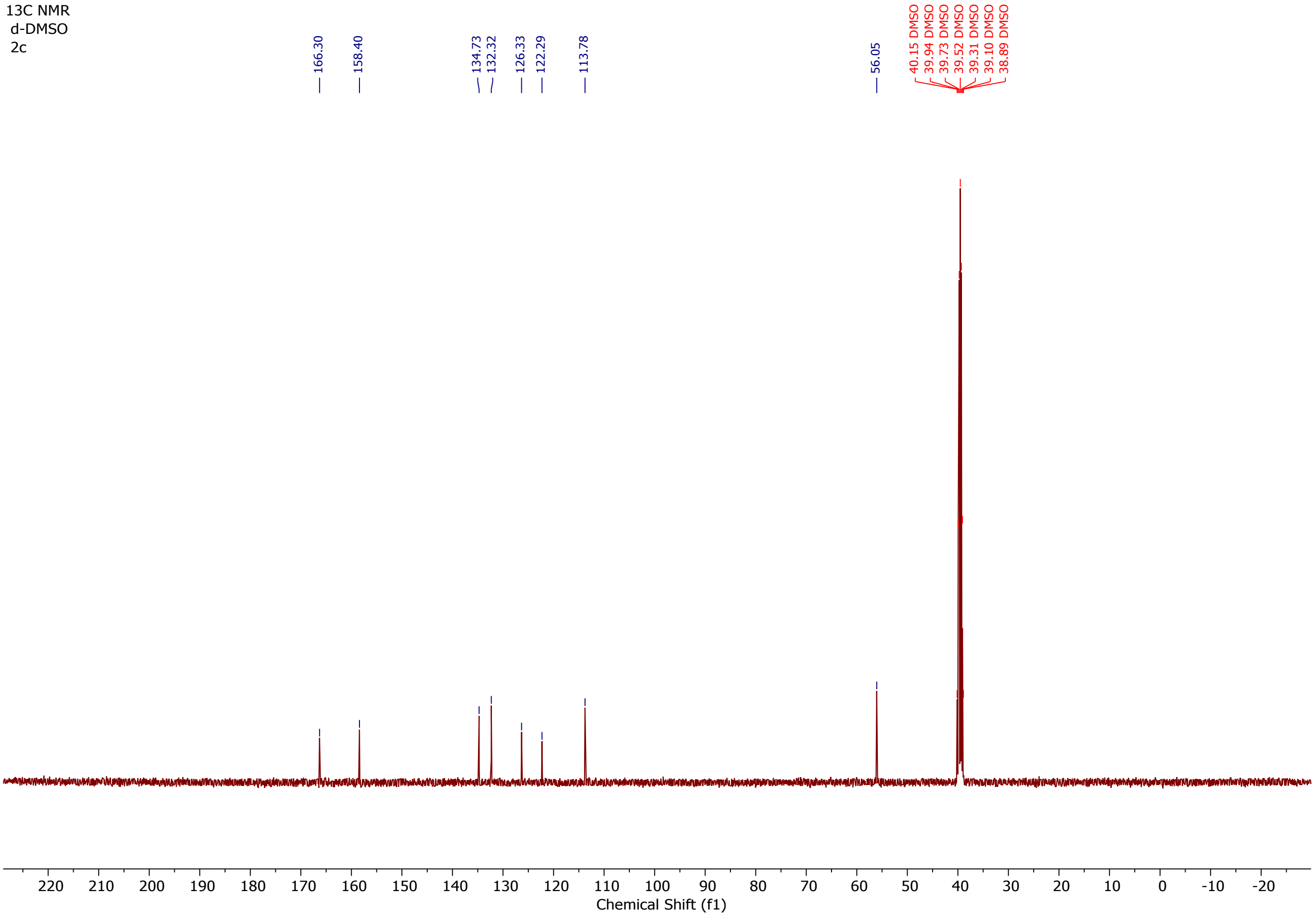

2-nitro-5-(octyldisulfaneyl)benzoic acid (4aa)

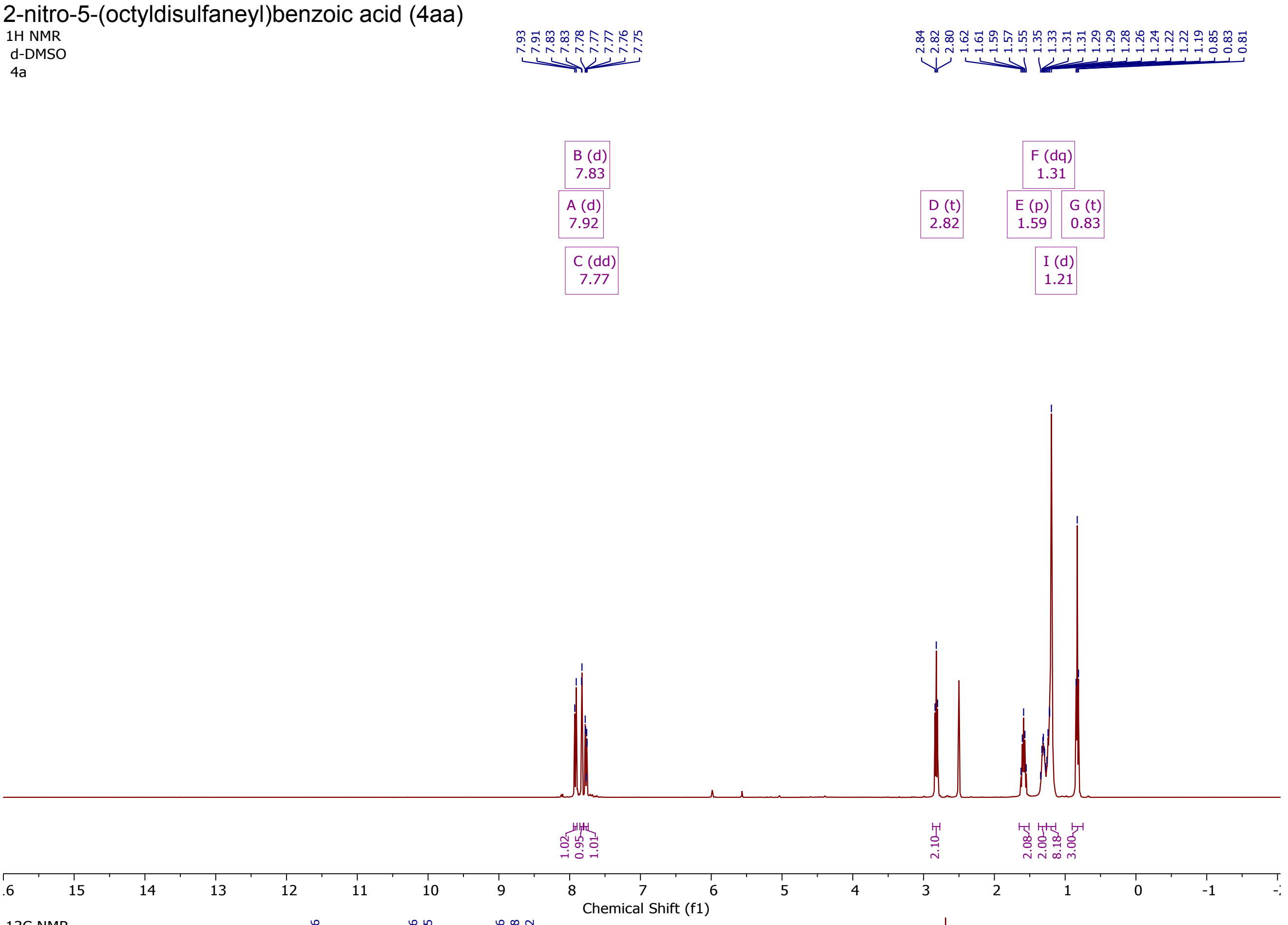

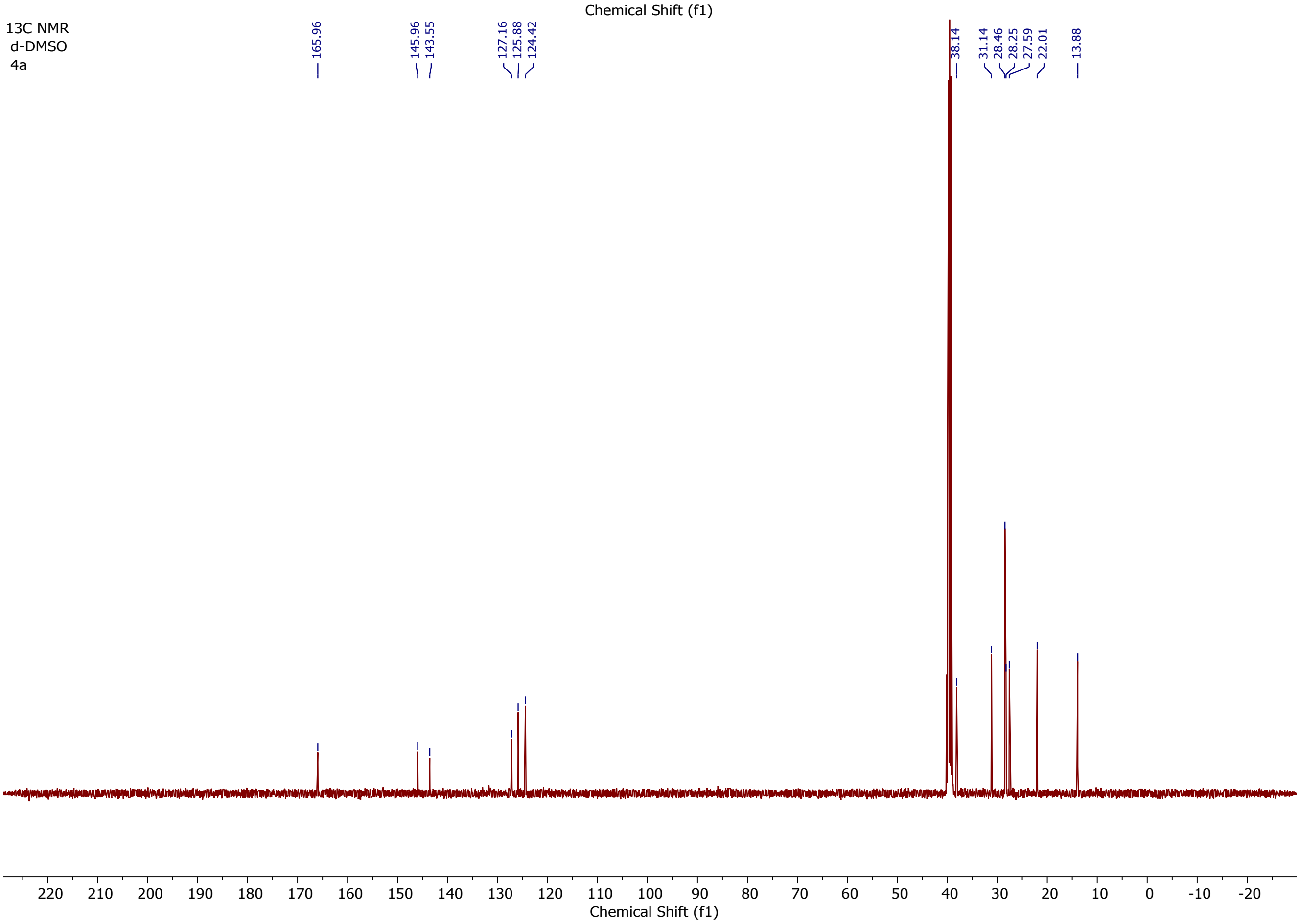

2-nitro-5-(nonan-2-yldisulfaneyl)benzoic acid (4ba)

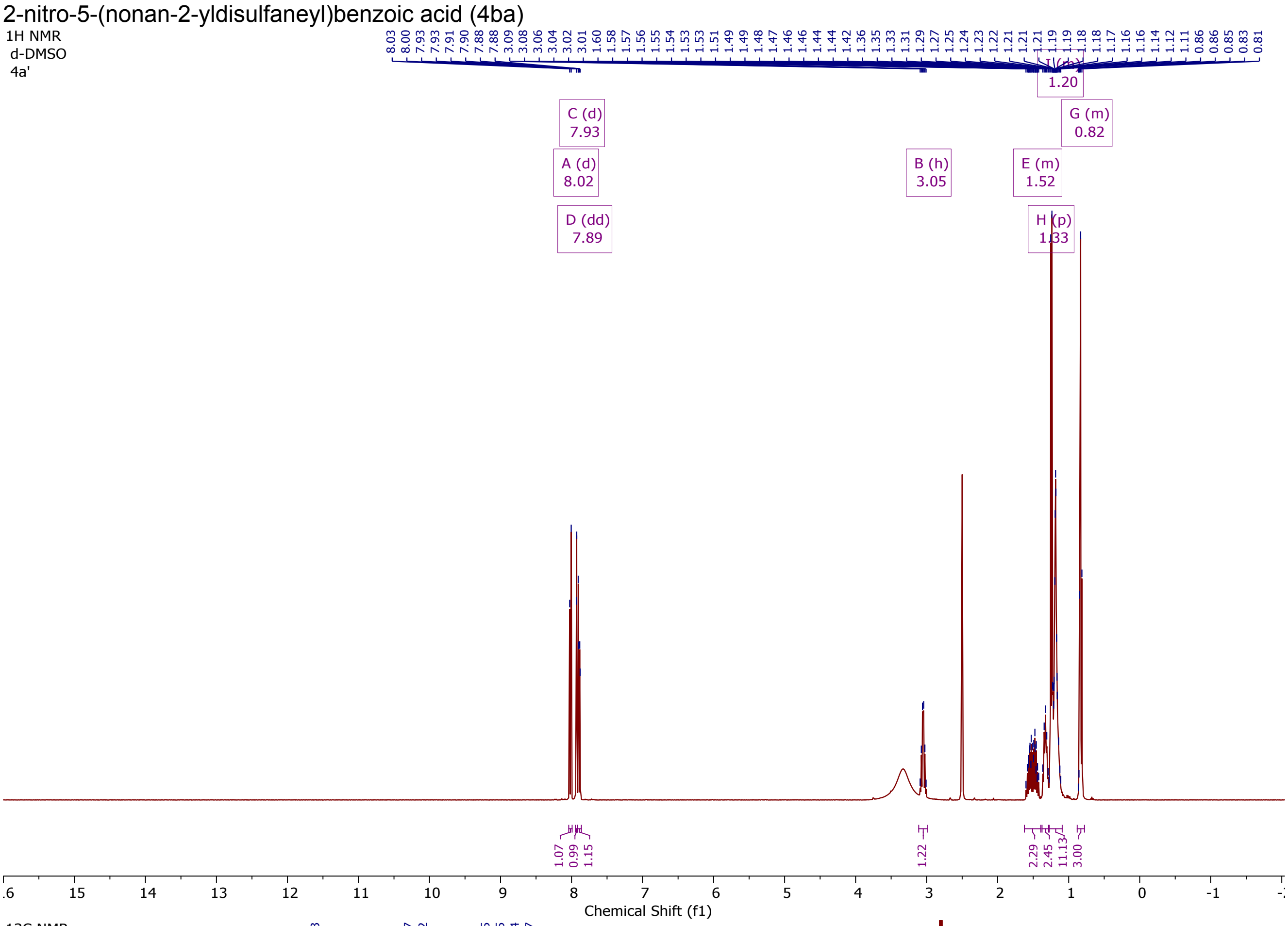

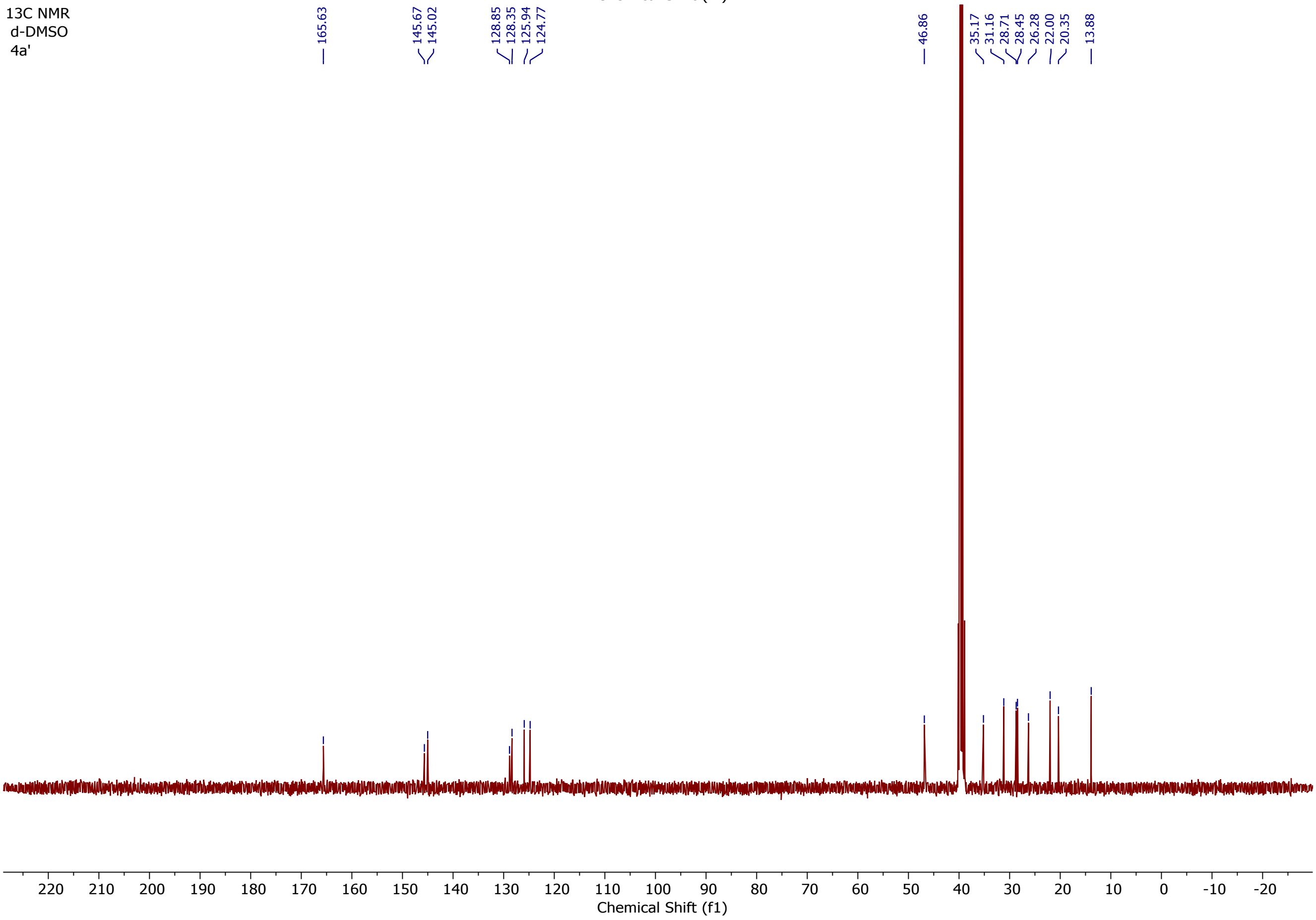

2-nitro-5-((2-methylnonan-2-yl)disulfaneyl)benzoic acid (4ca)

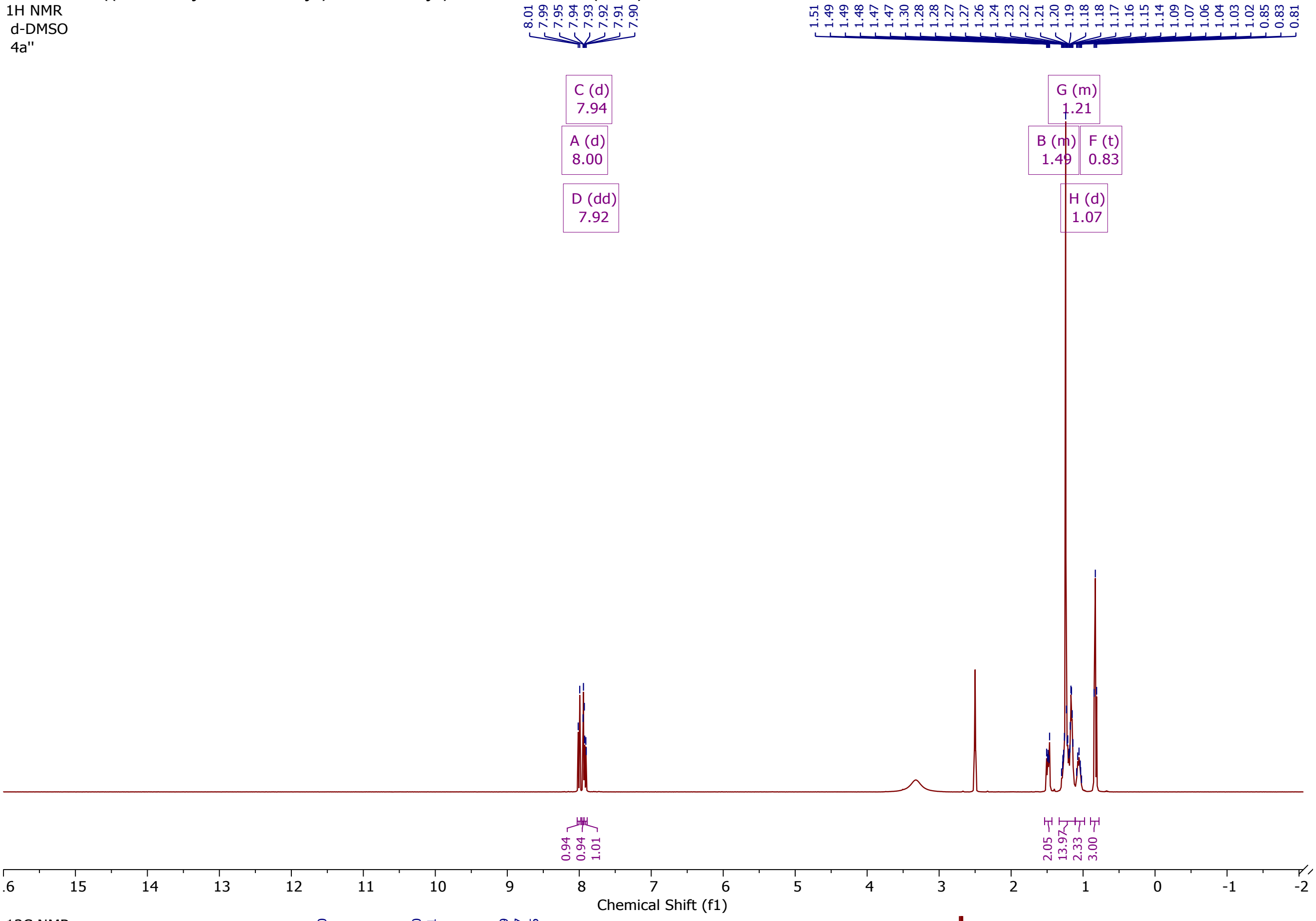

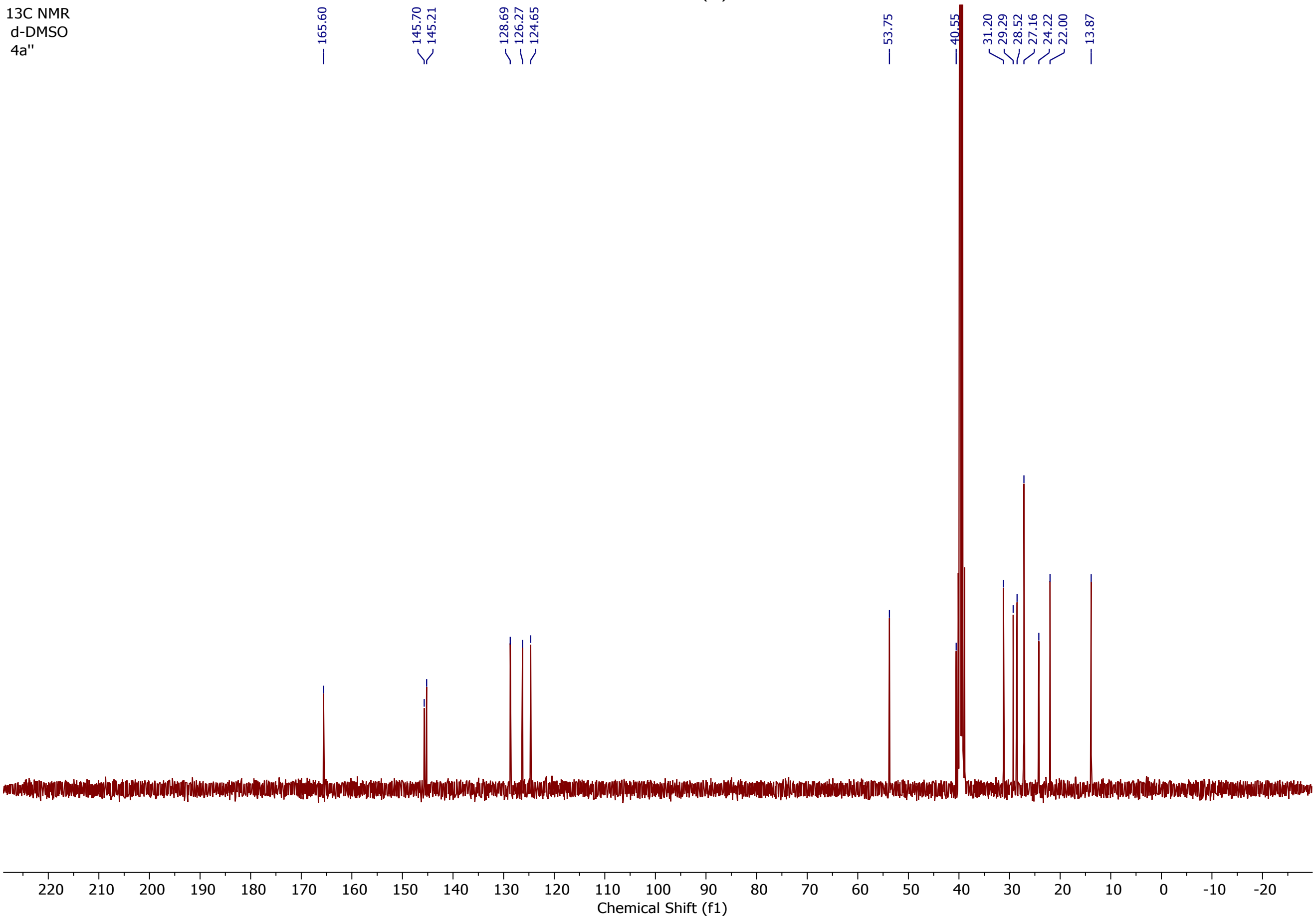

3-(octyldisulfaneyl)benzoic acid (4ab)

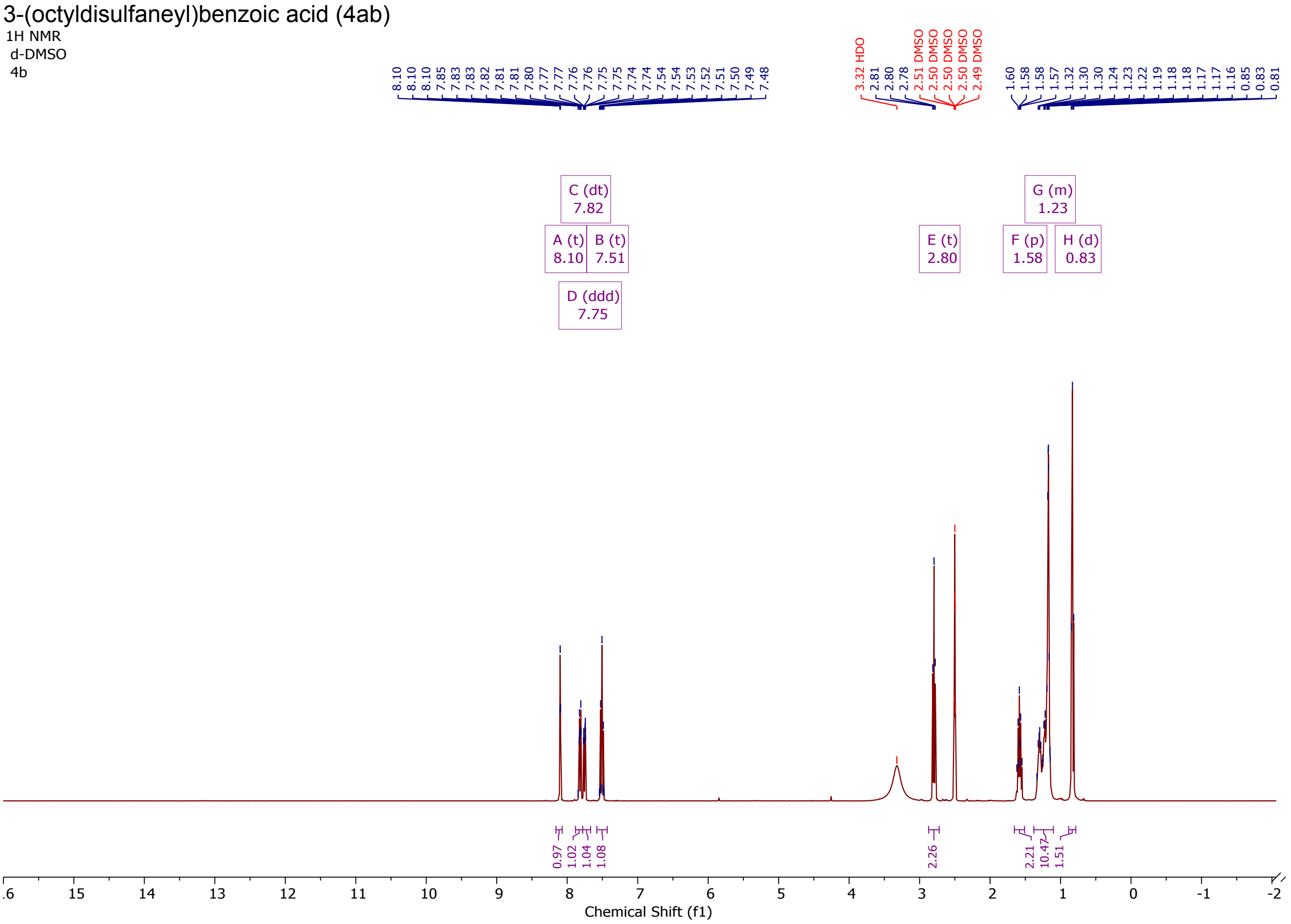

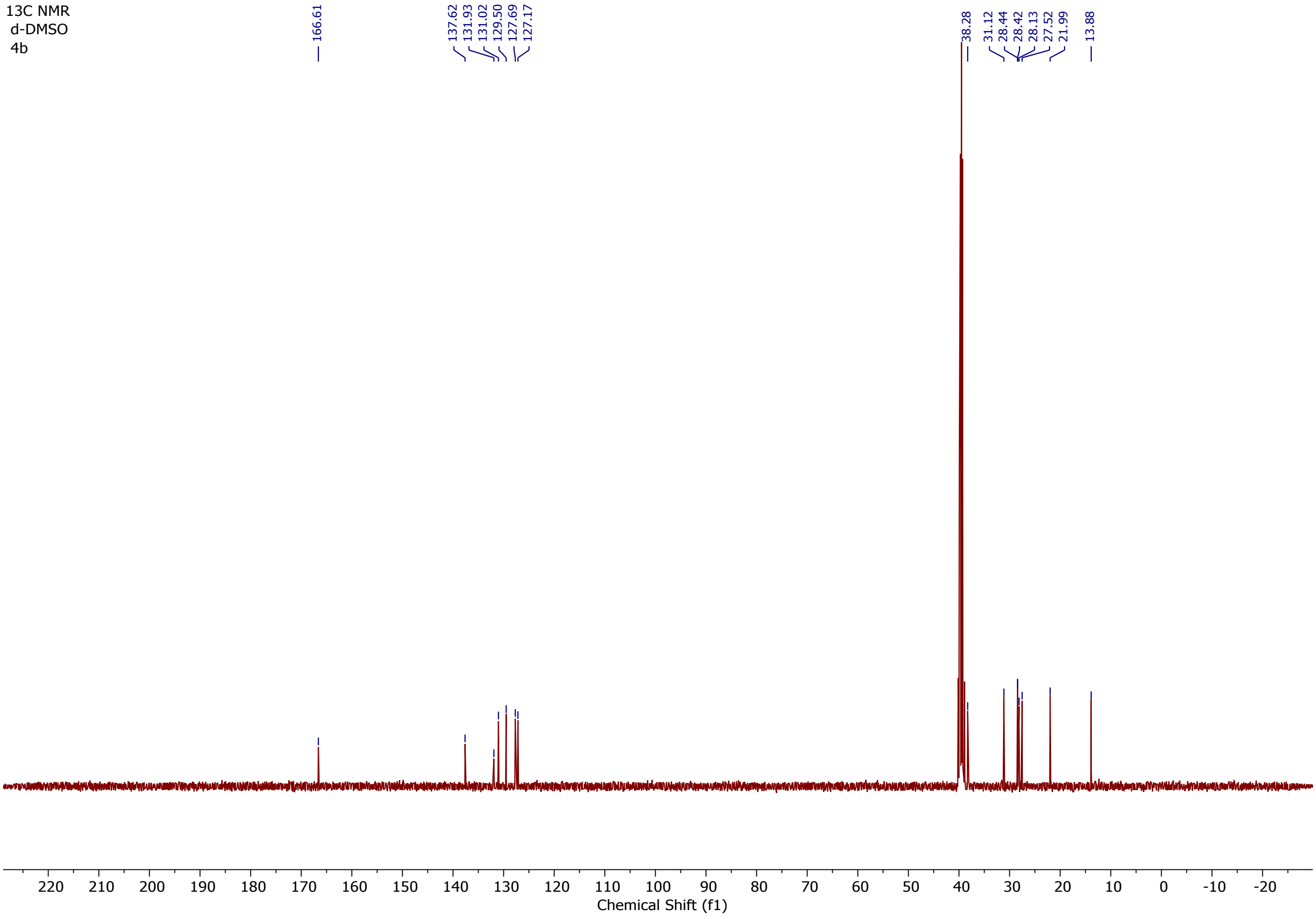

3-(nonan-2-yldisulfaneyl)benzoic acid (4bb)

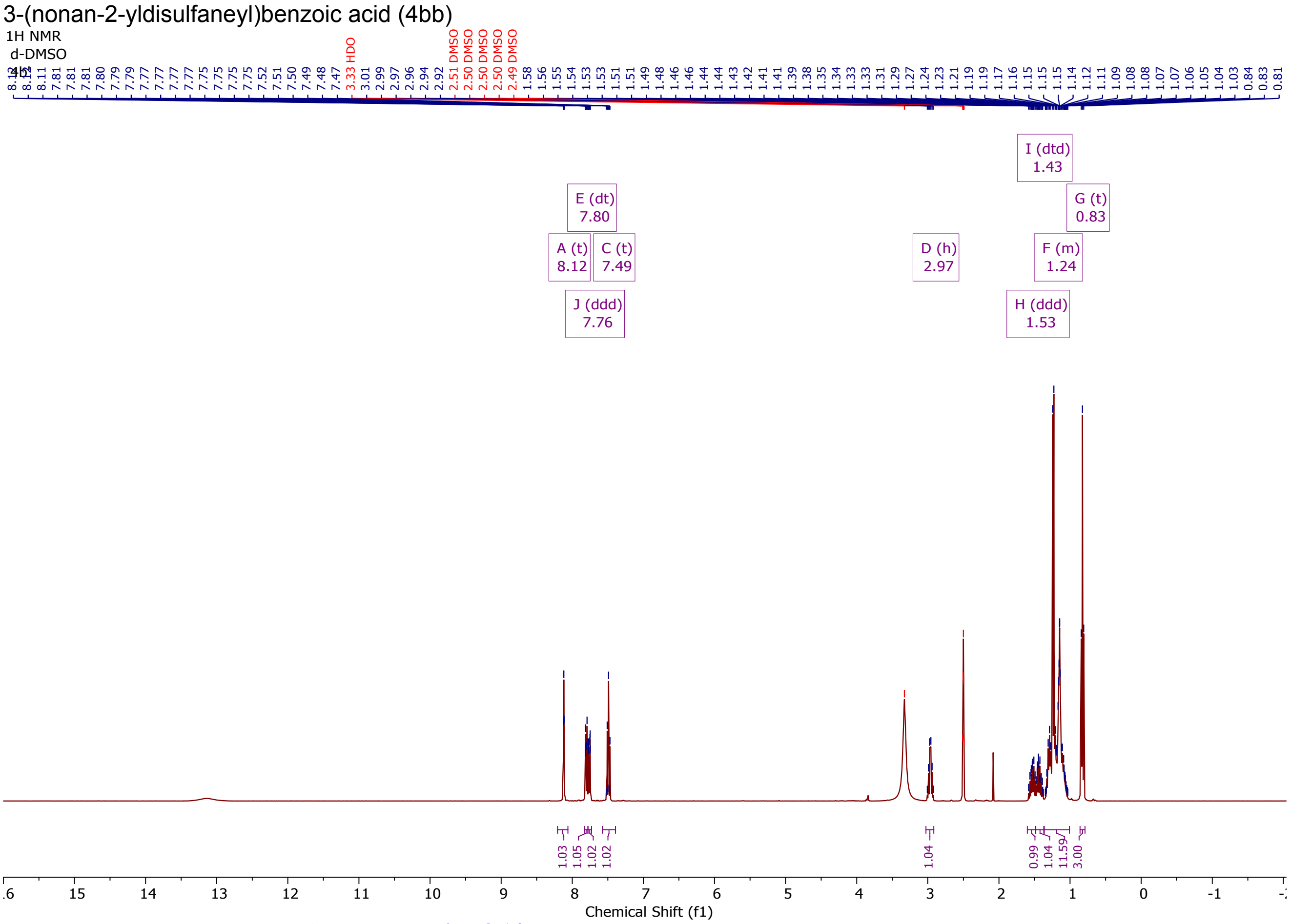

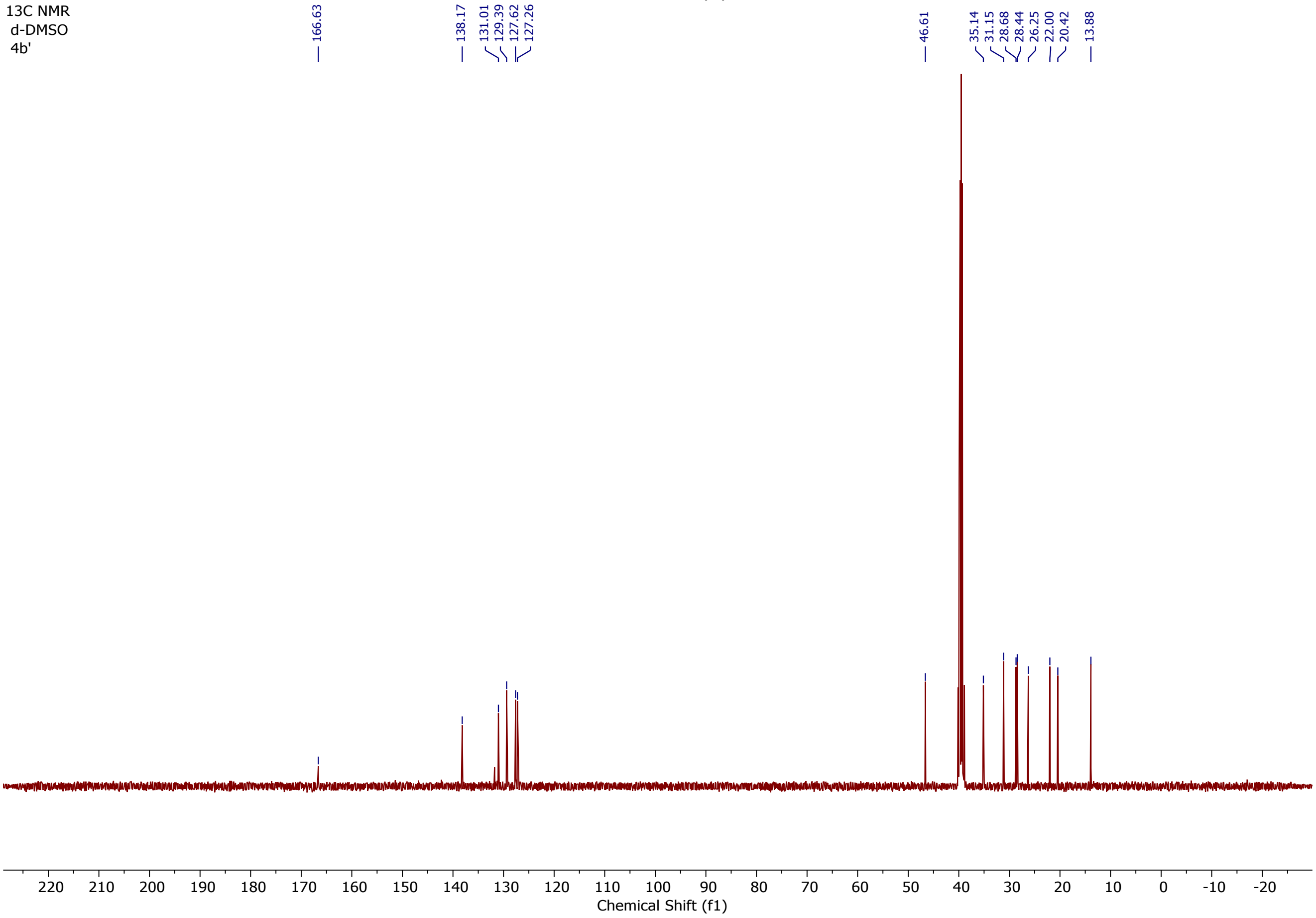

3-(2-methylnonan-2-yl)disulfaneyl)benzoic acid (4cb)

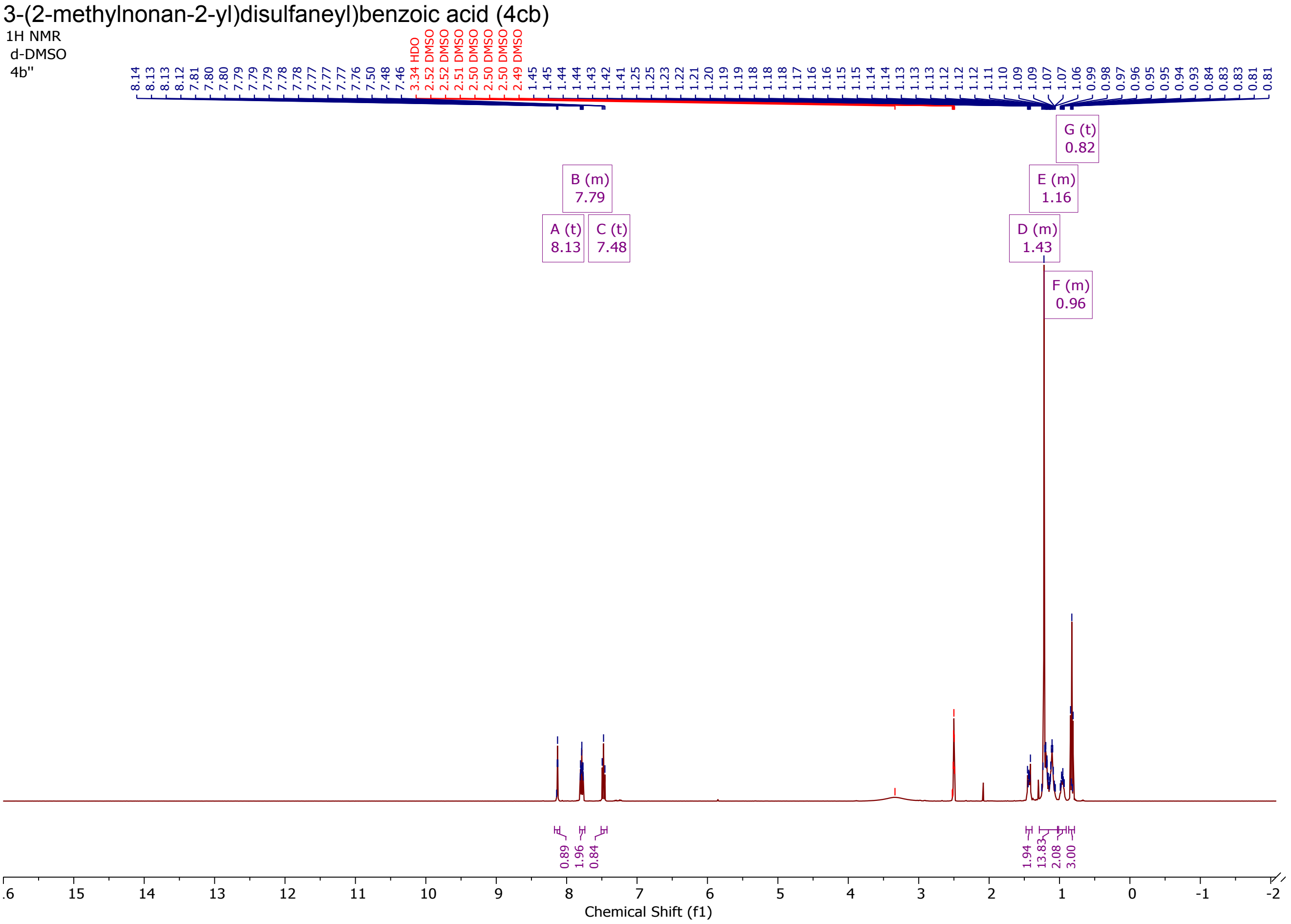

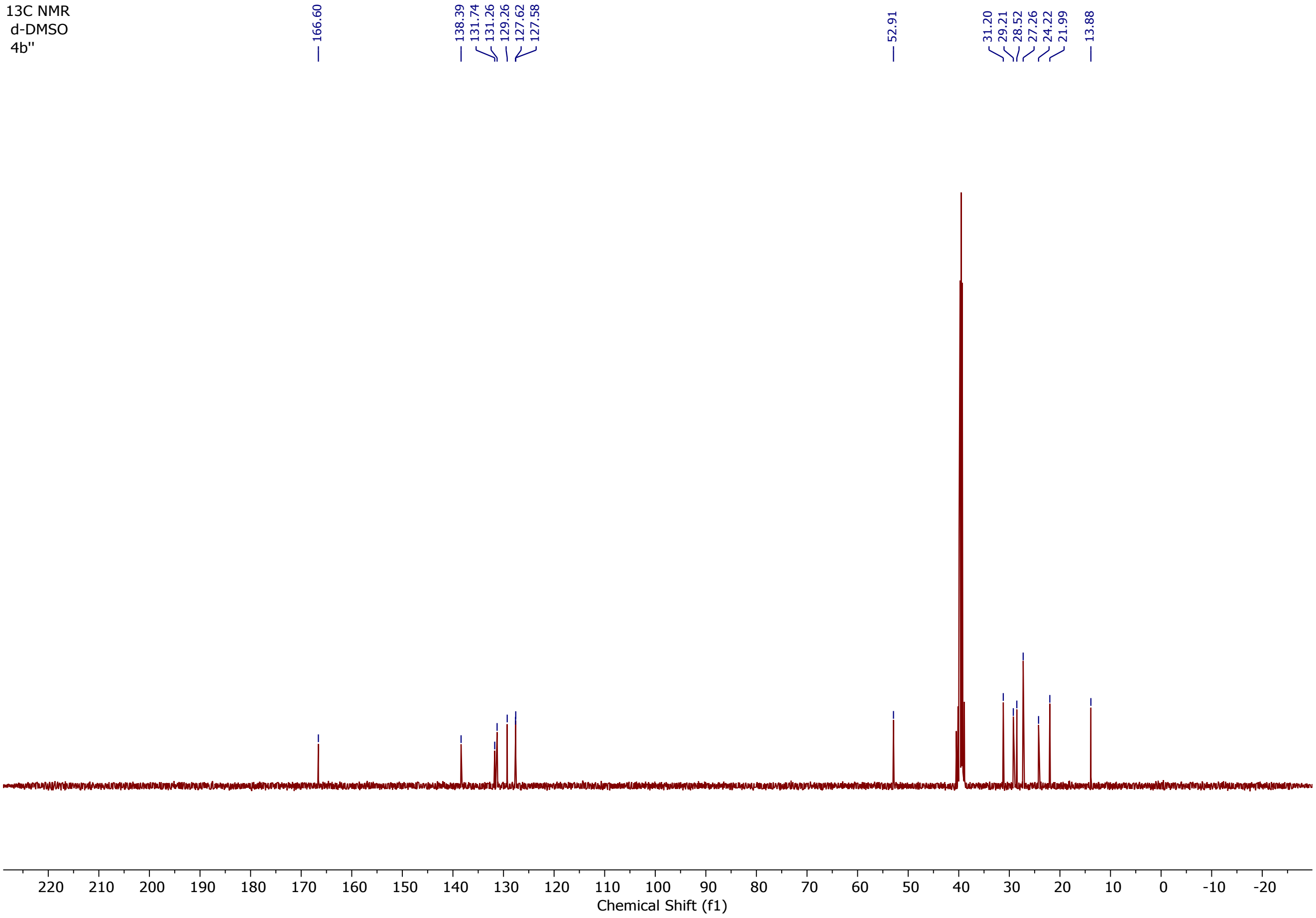

2-methoxy-5-(octyldisulfaneyl)benzoic acid (4ac)

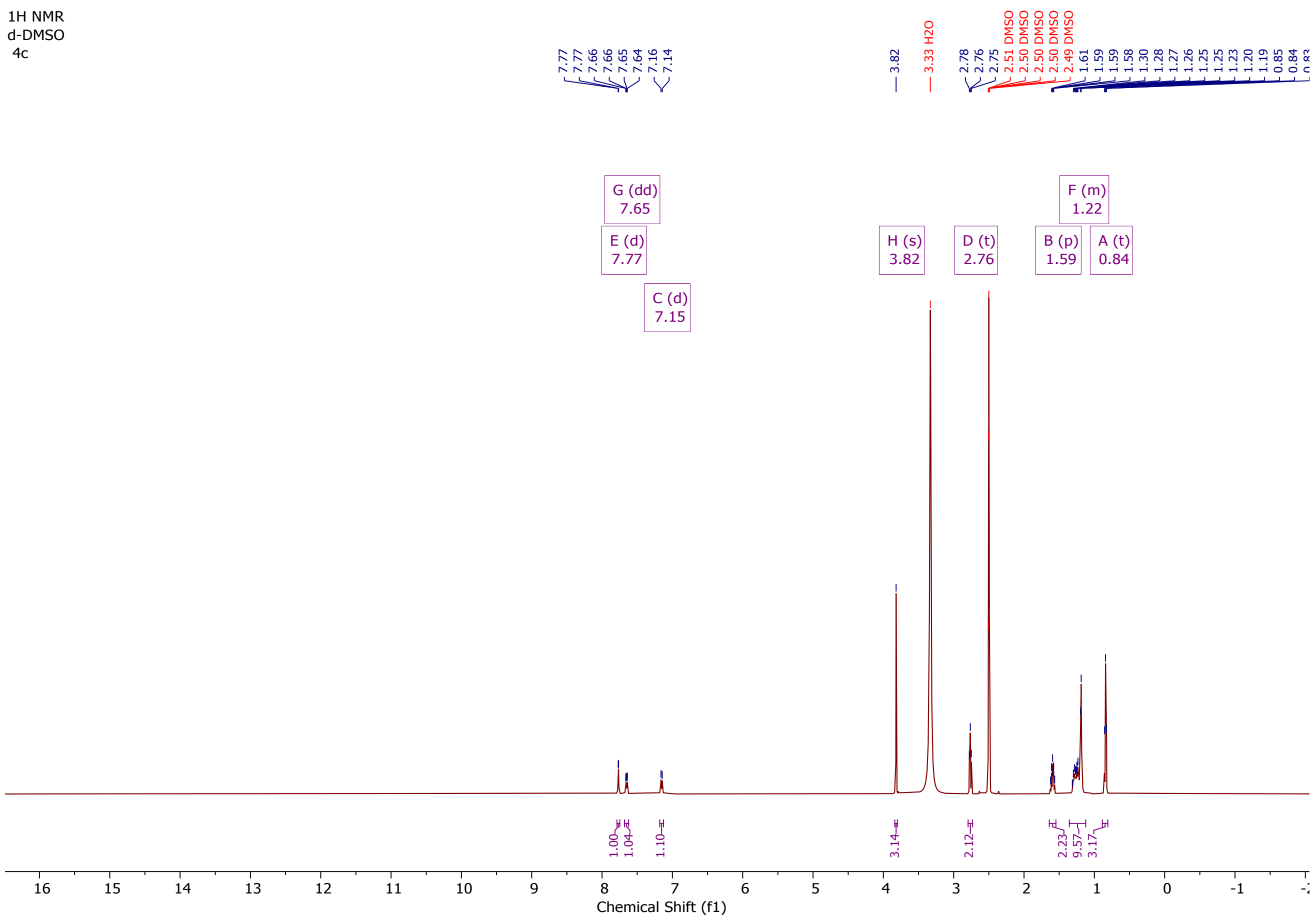

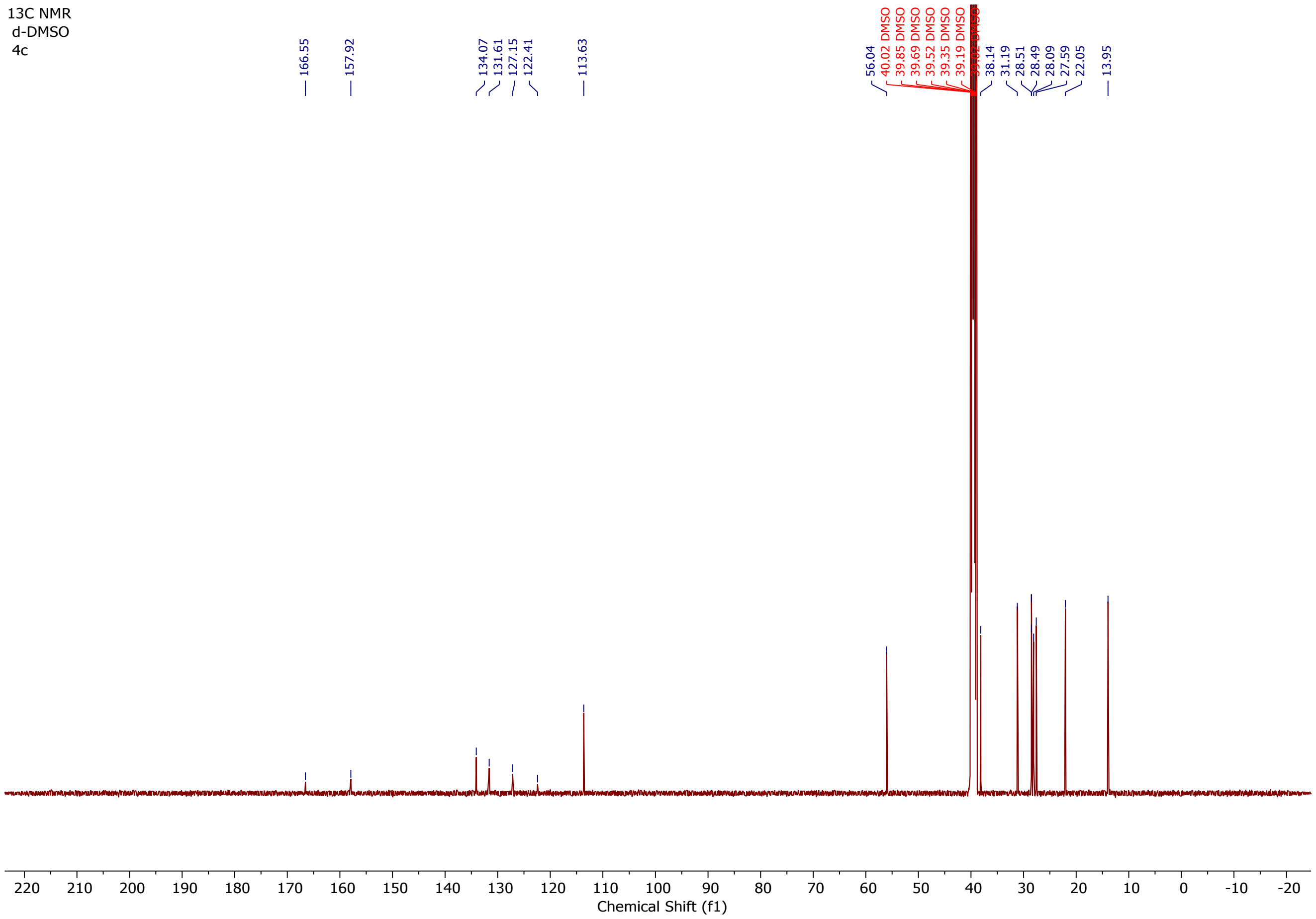

2-methoxy-5-(nonan-2-yldisulfaneyl)benzoic acid (4bc)

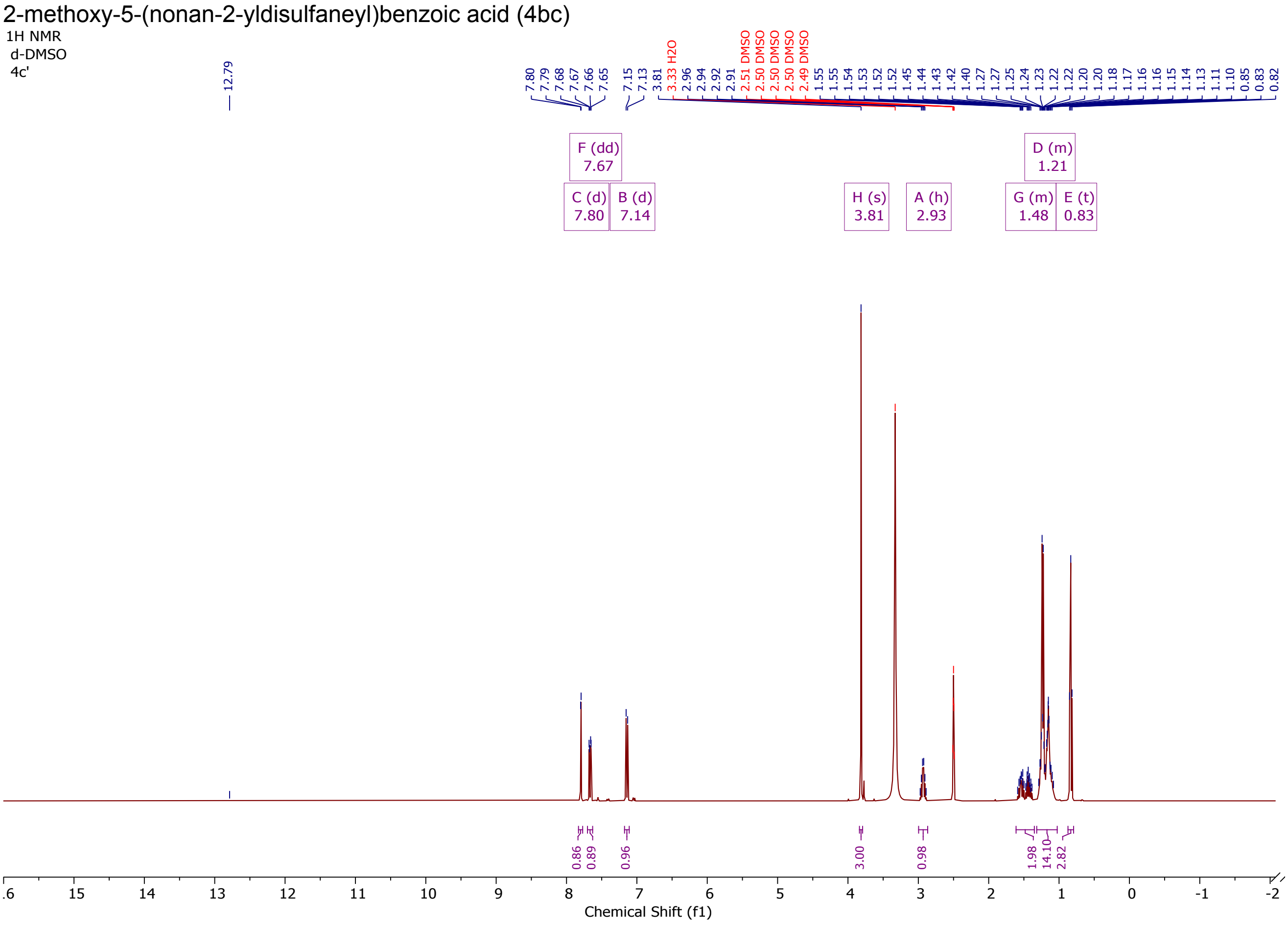

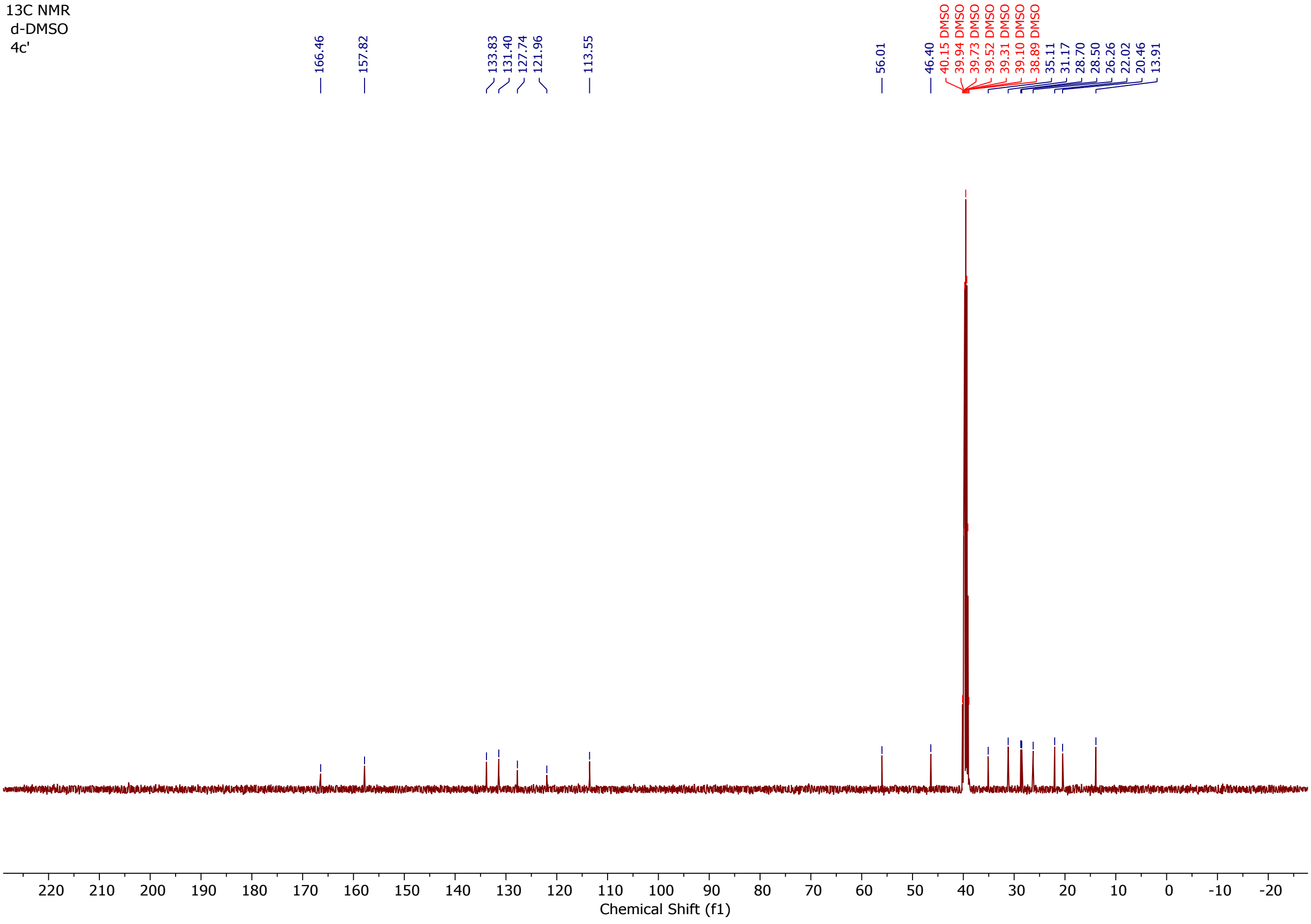

2-methoxy-5-(2-methylnonan-2-yl)disulfaneyl)benzoic acid (4cc)

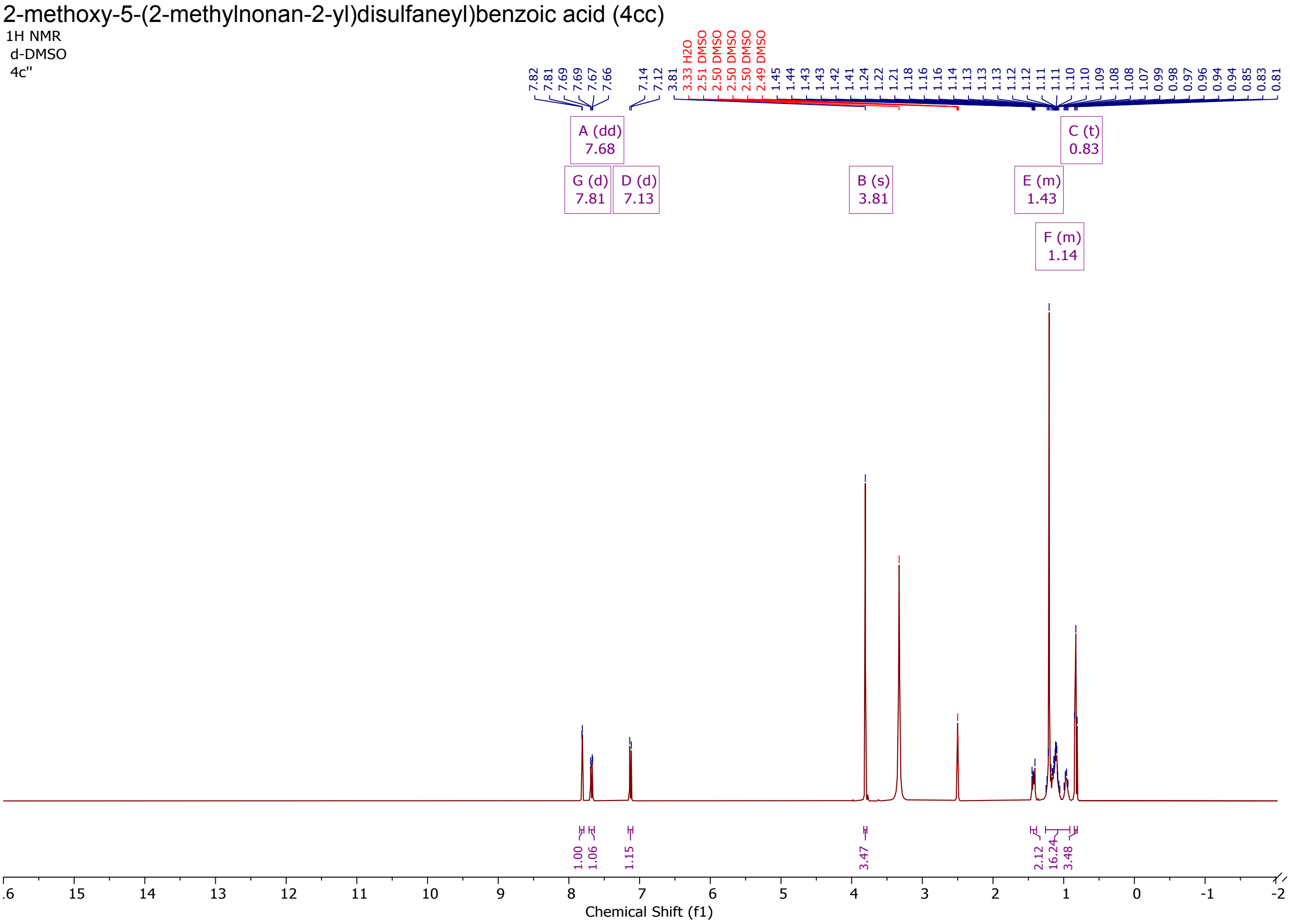

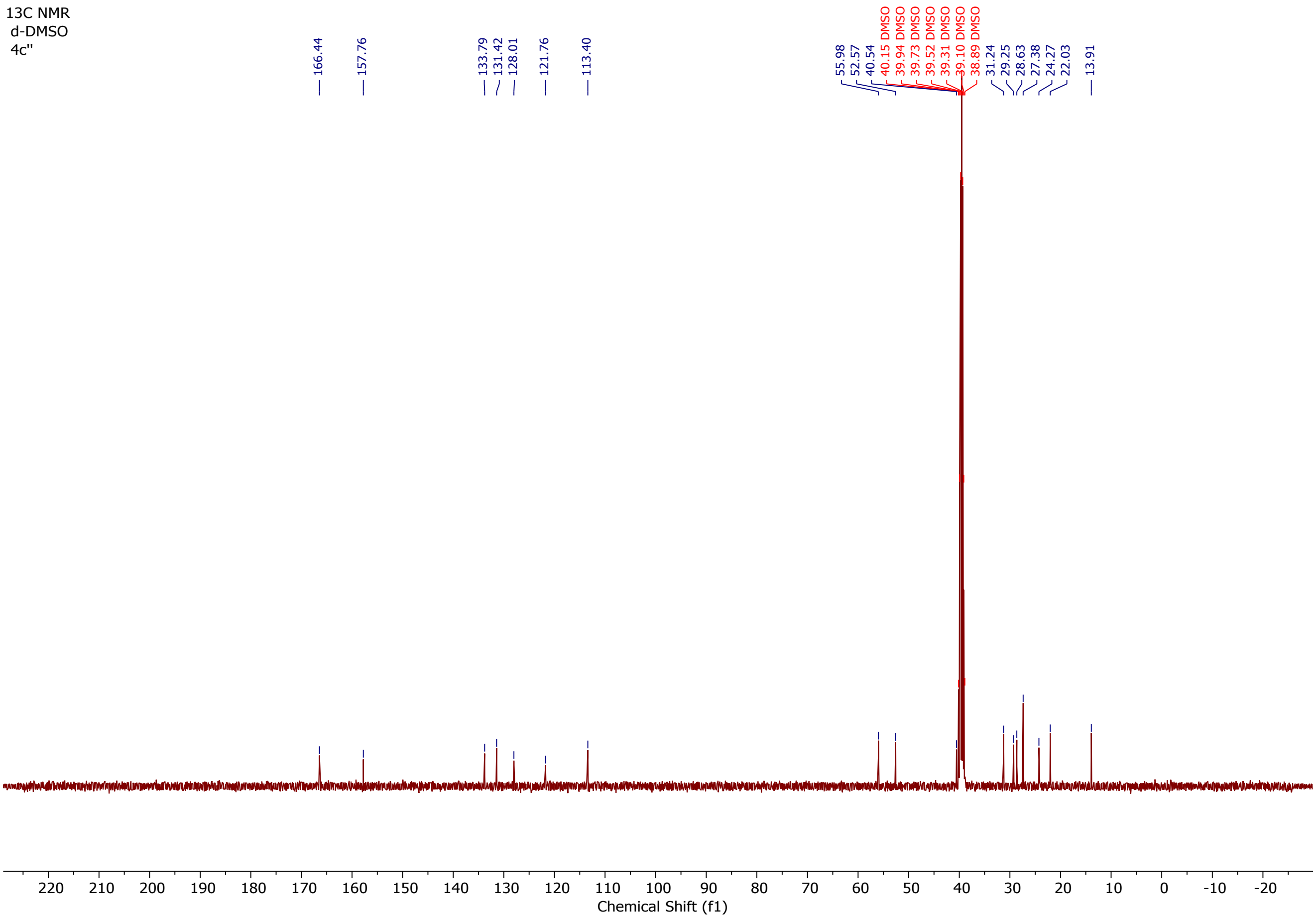

Additional surfactant characterisation

Ring tensiometry measurements

The critical aggregation concentrations (CACs) of surfactants were determined with a Krus K10ST tensiometer. Measurements and calibration were performed at room temperature. Surface tensions were measured for a dilution series of each surfactant in HEPES buffer (0.25 M, pH 8.00), typically in a concentration range of 0.024-12.5 mM, using a volume of 3.0 mL per sample. Each measurement was performed in triplicate. CACs are found at the concentration which there is a change of linear regime between surface tension and natural logarithm of surfactant concentration. Solutions of the **4aa-ca** series all appeared transparent, whilst those of the other two series (**4ab-cb**, **4ac**-**cc**) appeared turbid, suggesting the presence of large aggregates such as vesicles.

Table S13. Critical micelle concentrations (CAC) for all nine surfactants (**4**) as determined by ring tensiometry

Compound	CAC /μM
**4aa**	473
**4ba**	294
**4ca**	83
**4ab**	290
**4bb**	234
**4cb**	188
**4ac**	560
**4bc**	284
**4cc**	156

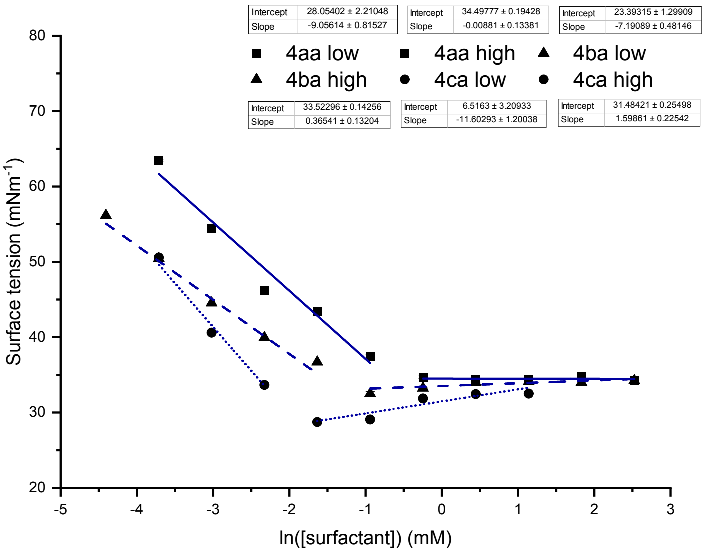

*Figure S28 CAC determination by ring tensiometry for surfactants **4aa** (squares, solid line, 473 μM), **4ba** (triangles, dashed line, 294 μM) and **4ca** (circles, dotted line, 83 μM)*

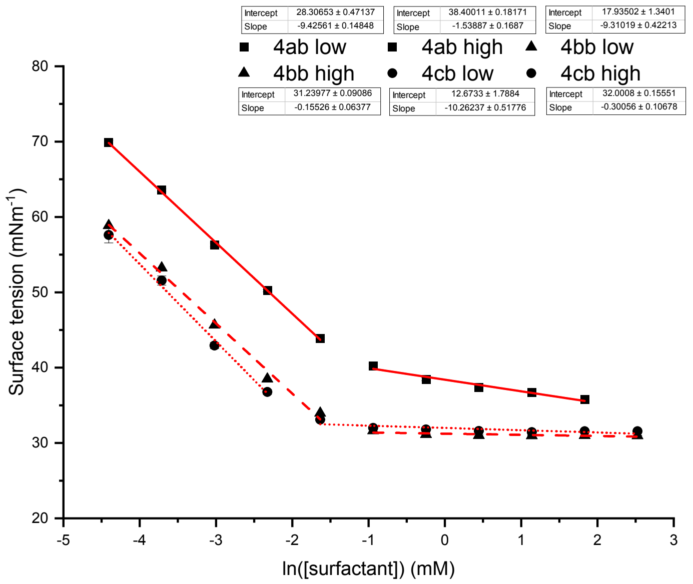

*Figure S29 CAC determination by ring tensiometry for surfactants **4ab** (squares, solid line, 290 μM), **4bb** (triangles, dashed line, 234 μM) and **4cb** (circles, dotted line, 188 μM)*

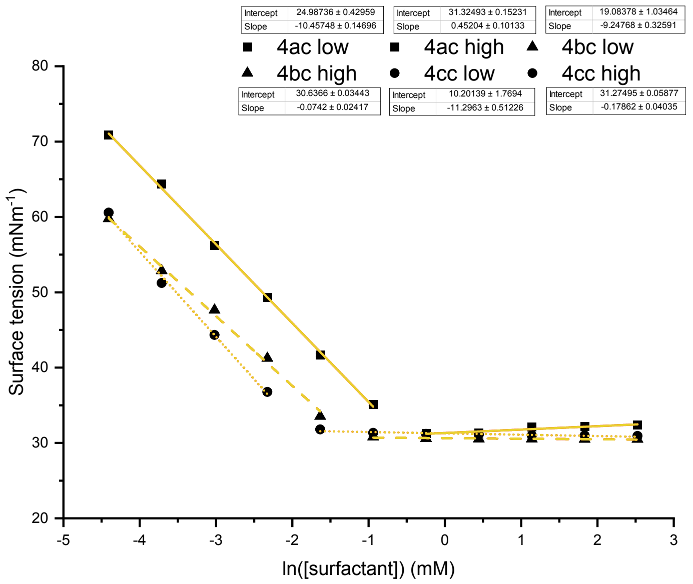

*Figure S30 CAC determination by ring tensiometry for surfactants **4ac** (squares, solid line, 560 μM), **4bc** (triangles, dashed line, 284 μM) and **4cc** (circles, dotted line, 156 μM)*

Dynamic light scattering (DLS) measurements

Dynamic light scattering (DLS) measurements were recorded using a Malvern Zetasizer Nano ZEN5600 machine and Zetasizer software for data processing. Samples were prepared as a serial dilution in HEPES buffer (pH 8.0) from 25.0 mM to 0.049 mM. Three measurements were taken for each sample. Refractive index of 1.371 and viscosity of 1.0780 mPas were used in data processing. At the extremes of this concentration range, multiple scattering events and low signal to noise led to artifacts.

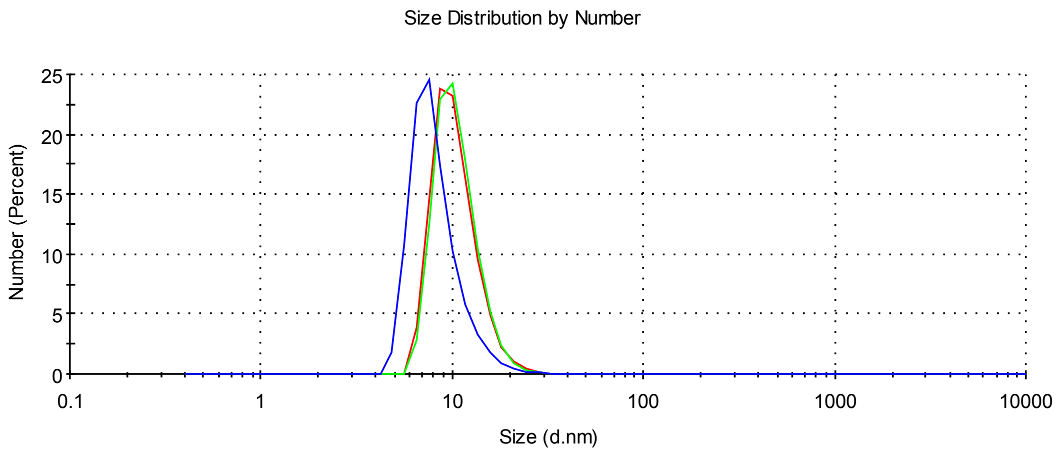

*Figure S31 Size distribution of **4aa** (12.5 mM) by DLS. Mean aggregate size = 9.8 nm*

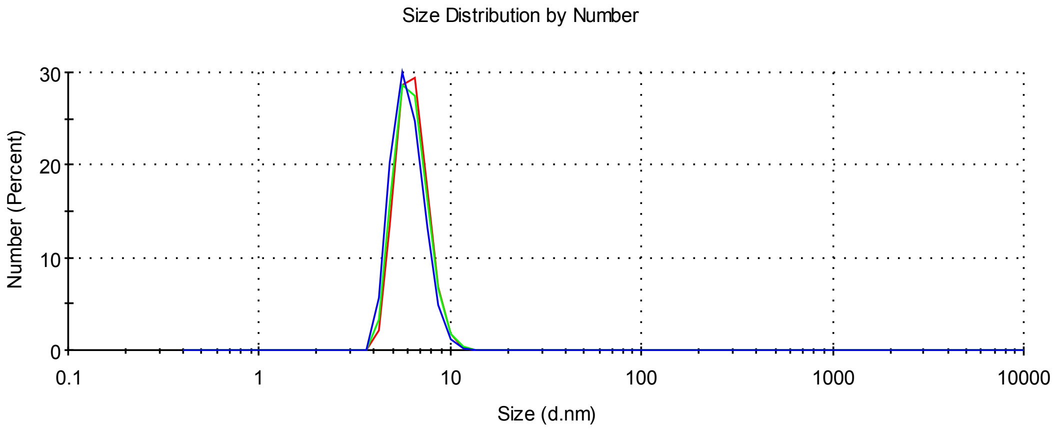

*Figure S32 Size distribution of **4ba** (12.5 mM) by DLS. Mean aggregate size = 6.3 nm*

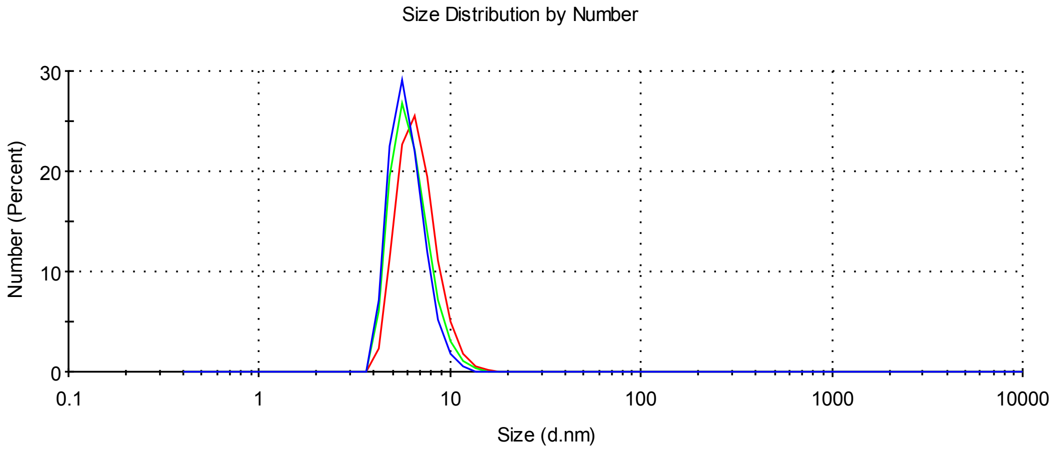

*Figure S33 Size distribution of **4ca** (25.0 mM) by DLS. Mean aggregate size = 6.4 nm*

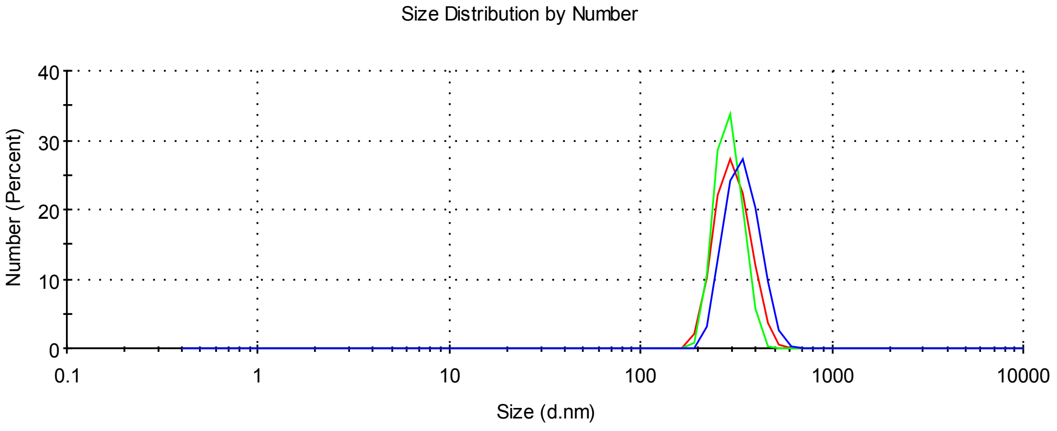

*Figure S34 Size distribution of **4ab** (0.195 mM) by DLS. Mean aggregate size = 313.4 nm*

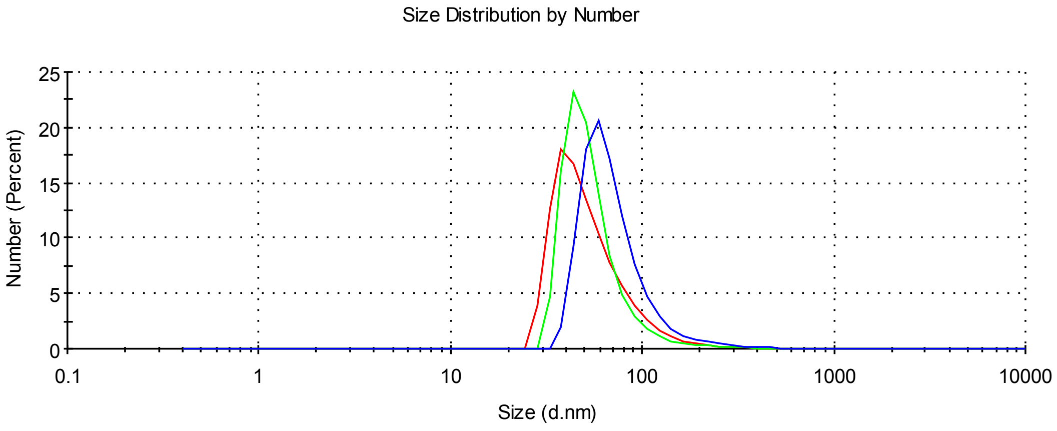

*Figure S35 Size distribution of **4bb** (3.125 mM) by DLS. Mean aggregate size = 62.9 nm*

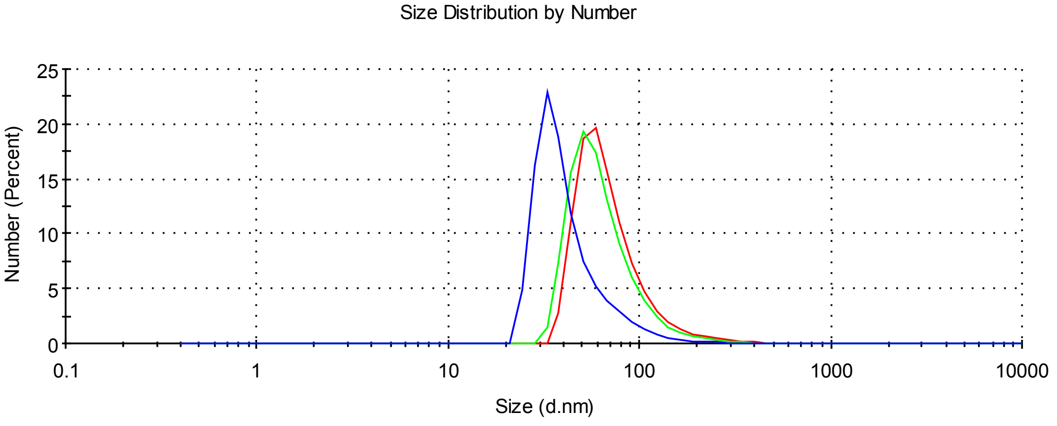

*Figure S36 Size distribution of **4cb** (0.195 mM) by DLS. Mean aggregate size = 61.8 nm*

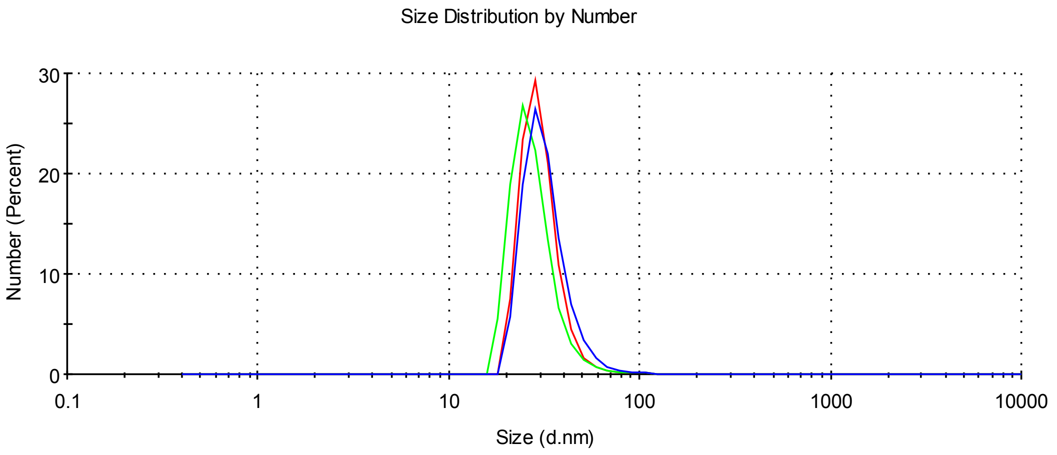

*Figure S37 Size distribution of **4ac** (25.0 mM) by DLS. Mean aggregate size = 30.5 nm*

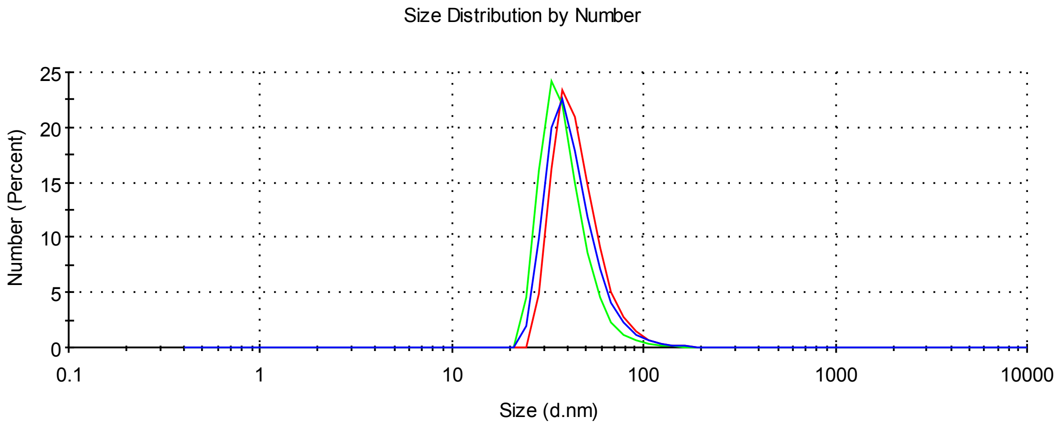

*Figure S38 Size distribution of **4bc** (1.56 mM) by DLS. Mean aggregate size = 43.3 nm*

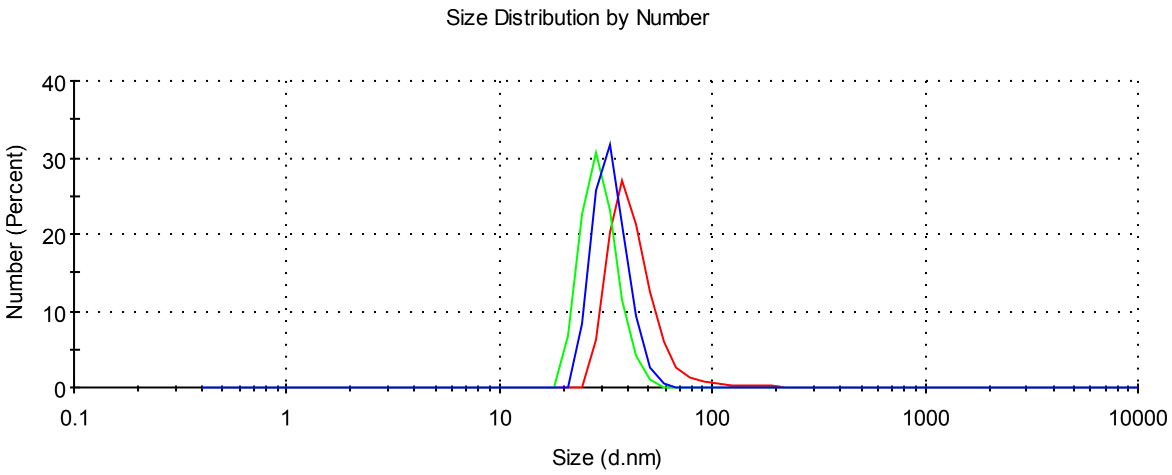

*Figure S39 Size distribution of **4cc** (0.195 mM) by DLS. Mean aggregate size = 36.2 nm*

References

(1) Post, E. A. J.; Fletcher, S. P. Dissipative Self-Assembly, Competition and Inhibition in a Self-Reproducing Protocell Model. *Chem. Sci.* **2020**, *11* (35), 9434–9442. https://doi.org/10.1039/d0sc02768e.

(2) Colomer, I.; Borissov, A.; Fletcher, S. P. Selection from a Pool of Self-Assembling Lipid Replicators. *Nat. Commun.* **2020**, *11* (1), 1–9. https://doi.org/10.1038/s41467-019-13903-x.

(3) Morrow, S. M.; Colomer, I.; Fletcher, S. P. A Chemically Fuelled Self-Replicator. *Nat. Commun.* **2019**, *10* (1), 1011. https://doi.org/10.1038/s41467-019-08885-9.

(4) Polster, J.; Schieberle, P. Structure–Odor Correlations in Homologous Series of Alkanethiols and Attempts To Predict Odor Thresholds by 3D-QSAR Studies. *J. Agric. Food Chem.* **2015**, *63* (5), 1419–1432. https://doi.org/10.1021/jf506135c.

(5) Dunn, P. J.; Levett, P. C. EU Pat. EP0994115, 2000.
